# Surface charge as activity descriptors for electrochemical CO_2_ reduction to multi-carbon products on organic-functionalised Cu

**DOI:** 10.1038/s41467-023-35912-7

**Published:** 2023-01-20

**Authors:** Carina Yi Jing Lim, Meltem Yilmaz, Juan Manuel Arce-Ramos, Albertus D. Handoko, Wei Jie Teh, Yuangang Zheng, Zi Hui Jonathan Khoo, Ming Lin, Mark Isaacs, Teck Lip Dexter Tam, Yang Bai, Chee Koon Ng, Boon Siang Yeo, Gopinathan Sankar, Ivan P. Parkin, Kedar Hippalgaonkar, Michael B. Sullivan, Jia Zhang, Yee-Fun Lim

**Affiliations:** 1 Institute of Materials Research and Engineering, Agency for Science, Technology and Research (A*STAR), 2 Fusionopolis Way, Innovis, Singapore, 138634 Singapore; 2 School of Materials Science and Engineering, Nanyang Technological University, 50 Nanyang Avenue, Singapore, 639798 Singapore; 3 Department of Chemistry, University College London, 20 Gordon Street, London, WC1H 0AJ UK; 4 Institute of High Performance Computing, Agency for Science, Technology and Research (A*STAR), 1 Fusionopolis Way, Connexis, Singapore, 138632 Singapore; 5 Department of Chemistry, National University of Singapore, 3 Science Drive 3, Singapore, 117543 Singapore; 6 Research Complex at Harwell, Rutherford Appleton Laboratory, Harwell Science and Innovation Campus, Didcot, Oxfordshire OX11 0FA UK; 7 Institute of Sustainability for Chemical, Engineering and Environment, Agency of Science, Technology and Research (A*STAR), 1 Pesek Road, Singapore, 627833 Singapore

**Keywords:** Electrocatalysis, Electrocatalysis, Electrochemistry

## Abstract

Intensive research in electrochemical CO_2_ reduction reaction has resulted in the discovery of numerous high-performance catalysts selective to multi-carbon products, with most of these catalysts still being purely transition metal based. Herein, we present high and stable multi-carbon products selectivity of up to 76.6% across a wide potential range of 1 V on histidine-functionalised Cu. In-situ Raman and density functional theory calculations revealed alternative reaction pathways that involve direct interactions between adsorbed histidine and CO_2_ reduction intermediates at more cathodic potentials. Strikingly, we found that the yield of multi-carbon products is closely correlated to the surface charge on the catalyst surface, quantified by a pulsed voltammetry-based technique which proved reliable even at very cathodic potentials. We ascribe the surface charge to the population density of adsorbed species on the catalyst surface, which may be exploited as a powerful tool to explain CO_2_ reduction activity and as a proxy for future catalyst discovery, including organic-inorganic hybrids.

## Abstract (web summary)

Tuning CO_2_ electrocatalysis to achieve multi-carbon products is interesting yet challenging. Here the authors report a histidine-functionalized Cu catalyst for CO_2_ reduction to multi-carbon products and reveal the correlation between catalyst surface charge and catalytic performance.

## Introduction

Synergised efforts in recycling, energy recovery, biomass utilisation, and CO_2_ capture and utilisation (CCU) are required to realise a circular carbon economy with net-zero greenhouse gas emissions^1^. Electrochemical CO_2_ reduction (CO_2_RR) is arguably one of the more attractive CCU approaches because of its ability to tailor the catalytic reaction towards higher-value chemical products^2^, whilst tapping off-peak electricity on demand. However, the feasibility of CO_2_RR is contingent on improvement in the stability, Faradaic and energetic efficiencies of the process^3^.

Decades of intensive research on Cu-based catalysts^4,5^ have revealed intricate and interwoven relationships of intermediate species adsorption^6^, defects^7^, catalyst states^8,9^, and reaction conditions^10,11^ that collectively govern the selectivity and activity of CO_2_RR. However, experimentally observed activities are still frequently lower than theoretical predictions, suggesting the challenge to understand and accurately model macroscopic and dynamic factors that affect electrocatalysis. Attempts to improve the understanding of the dynamic factors in electrocatalysis have prompted the use of various AC/DC voltammetry techniques beyond established techniques like cyclic/linear sweep voltammetry (CV/LSV) and electrochemical impedance spectroscopy (EIS). Recent investigation on oxygen evolution (OER) catalysis revealed the interplay between applied voltage, accumulated surface charge, catalyst oxidation state change, and reaction rate^12^. However, it is unclear if such a relationship applies to CO_2_RR, especially to C_2+_ products (molecules with ≥2 carbons) where multiple electrochemical and non-electrochemical steps are involved^13^. Further, CO_2_RR catalysts (typically transition metals) are not commonly expected to display oxidation state cycling. CO_2_RR activities have also been shown to be less sensitive to electrochemical surface area (ECSA)^14^, and disconnected from the cardinal Tafel values^15^.

Herein, we exploit Cu_2_O-derived Cu with organic functionalisation to unveil the possible role of surface charge in CO_2_RR to C_2+_ products. Serendipitously, organic functional groups also provide an avenue to circumvent scaling relationships observed with transition metal-only catalysts^16^, by providing different bonding configurations to CO_2_RR intermediates^17^. Increased sensitivity between voltage and catalytic activity is also often observed on organic-functionalised catalysts, a phenomenon that points to surface charge accumulation^12,18^. Histidine is selected as the prime organic functionalisation due to its ability to bind strongly to Cu^19^ while containing an imidazole group that can bind and activate CO_2_^17^. Cu_2_O-derived Cu functionalised with histidine (Cu-Hist) displays significantly higher C_2+_ product selectivity than the unfunctionalized sample across a wide voltage range. Faradaic efficiencies (FE) of up to 76.6%, corresponding to TOF estimate up to 4.2 × 10^−1^ s^−1^ for C_2+_ products, were observed at −2.0 V (vs. reversible hydrogen electrode, RHE throughout), and excellent stability over 48 h. Through careful materials characterisation, in-situ Raman spectroscopy, and density functional theory (DFT) calculations, we discover that the enhanced CO_2_RR to C_2+_ products on functionalised Cu-Hist is linked to direct intermediate interaction with adsorbed histidine. Strikingly, we uncovered a strong correlation between surface charge magnitude and catalytic activity, suggesting that the electrocatalytic activity in reductive catalysis like CO_2_RR may also be closely linked to surface charge.

## Results and discussions

### Electrochemical CO_2_ reduction data

**Fig. 1:**
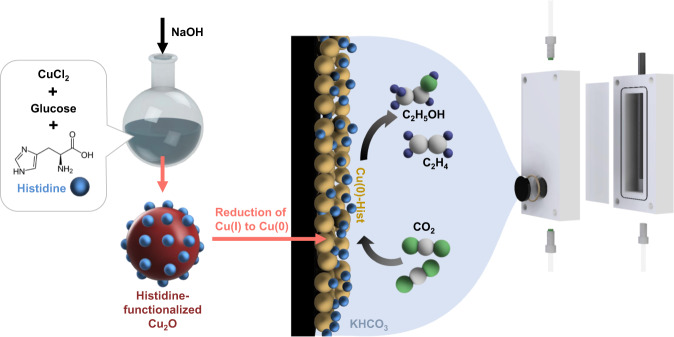
Preparation of histidine functionalised Cu_2_O and its subsequent use in CO_2_RR. The histidine remains on the surface after the in-situ reduction of Cu_2_O to Cu during catalysis, boosting reaction selectivity towards C_2+_ products. Other organic functionalisations (imidazole, 2-methylimidazole, imidazolepropionic acid, arginine, triazole, and glycine) can be introduced by swapping the reagents during synthesis.

**Fig. 2:**
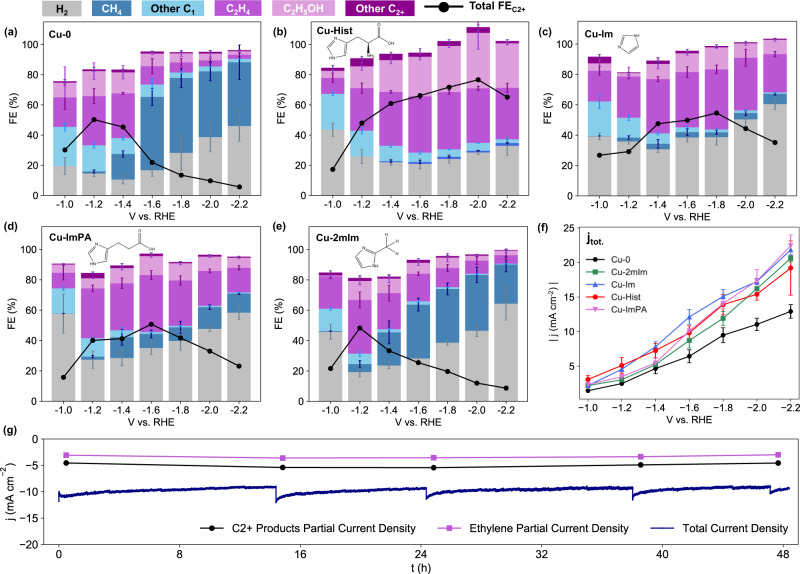
CO_2_RR performance comparison between functionalised and organic-functionalised Cu_2_O catalysts using different metrics. FE comparison of Cu_2_O-derived Cu with and without surface functionalisation group: **a** Cu-0, **b** Cu-Hist, **c** Cu-ImPA, **d** Cu-Im, **e** Cu-2mIm, and their respective, **f** total current density. **g** Stability of Cu-Hist at −1.6 V over 48 h. Dark blue line represents the total current density (*j*_tot_). Products were sampled 5 times at *t* = 0, 15, 24, 38, and 48 h for the stability experiment. Periodic dips seen in the *j*_tot_ plot are due to current re-stabilisation during electrolyte refresh. Error bars represent the standard deviation of three independent measurements.

The CO_2_RR performance of Cu-Hist was evaluated in a customised H-Cell (Fig. 1, see the “Methods” section and SI Sections 3 and 4). The CO_2_RR product distribution of Cu-Hist is very peculiar, with a significant preference towards C_2+_ products that stretches even to very wide cathodic potential up to −2.2 V (Fig. 2b). This is in stark contrast to plain Cu_2_O-derived Cu (Cu-0), where C_2+_ products are quickly overtaken by CH_4_ and H_2_ at cathodic potentials beyond −1.2 V (Fig. 2a), consistent with literature^20^. The production of C_2+_ products (particularly ethanol and ethylene) on Cu-Hist rose significantly with increasing voltage, reaching over 20× partial current density compared to Cu-0 (Fig. S4.5b, Table S3.1). Stable formation of C_2+_ products was also observed for 48 h (Fig. 2g). As there is no new metal component in Cu-Hist, and the ECSA of reduced samples are similar (SI Section 3.3), we posit that the significant enhancement in CO_2_RR selectivity may be caused by a new intermediate stabilisation involving strong chemical interactions with histidine alongside possible contributions from surface-charging effects.

To investigate the possible roles of the different functional groups in histidine, three additional similarly synthesised samples with imidazolium-related functionalisation were added: imidazole (Cu-Im), 2-methylimidazole (Cu-2mIm) and imidazolepropionic acid (Cu-ImPA). The selection of these molecules is based on their relation to histidine (see SI Section 1.3) and the samples’ characterisation is presented in SI section 2.

Intriguingly, distinct C_2+_ product selectivity and turnover trends were obtained from the four functionalised samples, despite their closely related surface functionalisation molecules. Enhanced C_2+_ product selectivity was also observed on Cu-Im and Cu-ImPA, albeit with lower peak FE (Fig. 2c, d) and current density (*j*) (Fig. S4.5b) than Cu-Hist. On the other hand, Cu-2mIm behaved more similarly to plain Cu-0, with peak FE_C2+_ occurring at much less cathodic potential of −1.2 V (Fig. 2e) alongside relatively flat *j*_C2+_ (Fig. S4.5b).

Overall, all functionalised catalysts display > 1.5× higher total current density (*j*_tot_) than Cu-0 (Fig. 2f), despite having similar ECSA (Fig. S3.2). Unlike previous studies on organic functionalised catalysts where enhanced CO_2_RR is obtained through suppressed HER via a significant increase in surface hydrophobicity^21^, a slightly enhanced HER was observed on all our functionalised catalysts at less cathodic potential (−1.0 to −1.2 V, Fig. S4.5a). With more cathodic potentials, HER was maintained at a similar level to uncapped Cu-0, while CO_2_RR activity is boosted more significantly, allowing us to achieve higher current densities. Overall, the *j*_C2+_ at −1.6 V or more cathodic potentials follow the trend of Cu-Hist ≫ Cu-Im > Cu-ImPA > Cu-2mIm ≥ Cu-0. Both FE and *j* trends confirm that functionalisation with imidazole-related molecules indeed enhances CO_2_RR to C_2+_, especially to C_2_H_4_ at more cathodic potentials. Prior literature links enhanced CO_2_RR activity on imidazole-related surface functionalisation to the unblocked no. 2 carbon position (C-2) in the imidazole ring (Fig. 1), as it can facilitate the capture of an extra proton with δ+ charge under cathodic potential^22^. However, the presence of the imidazole group alone cannot explain the >27% higher FE_ethanol_ observed on Cu-Hist compared to Cu-Im and Cu-ImPA, as all three functionalisation have the same imidazole group with unblocked C-2 position.

Thus, we hypothesise that the enhanced activity of the Cu-Hist sample to C_2+_ products, particularly to ethanol, may be due to histidine’s unique structure. A prerequisite for good surface functionalisation is the stability of the catalyst surface throughout the catalysis and reconstruction process^23^. Both Hist and ImPA are predicted to be the most stable among the five functionalisation molecules as both carboxylate oxygens and proxima nitrogen (N–π) can anchor to Cu (Fig. S1.1)^19^. On the other hand, only N–π nitrogen is available for Im and 2mIm, rendering them less stable. The adsorption of CO_2_ (or CO) can then be accommodated on the remaining nitrogen sites. Histidine is advantageous, with two N sites available (amine-N and the imidazolate tele-nitrogen, N–τ), compared to ImPA and Im with only one N–τ site. We posit that the presence of organic surface functionalization on the Cu surface can enhance the retention of these intermediate species, thereby favouring the formation of C_2+_ products while suppressing C_1_ products even at very cathodic potentials. To rationalise the CO_2_RR enhancement on Cu–Hist, we engaged a multi-pronged approach of in-situ Raman spectroscopy, dynamic voltammetry, and theoretical calculation.

### In-situ Raman spectroscopy

**Fig. 3:**
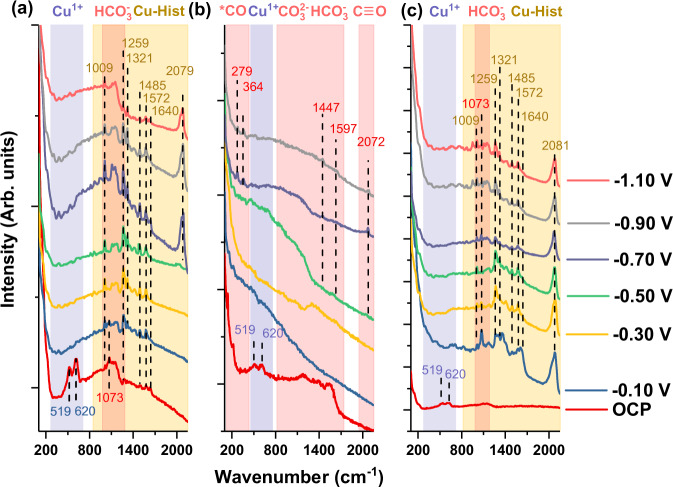
In-situ Raman spectroscopy on bare and histidine functionalised Cu_2_O under CO_2_RR relevant conditions. Comparisons were made on three different conditions to ascertain histidine presence during CO_2_RR and the expected *CO binding configuration on **a** electrodeposited Cu_2_O with added 0.025 M histidine dissolved in the electrolyte. **b** Cu-0 and **c** Cu-Hist. Measurements were stopped at different potentials depending on the vigorousness of the bubbling that disrupts in-situ Raman signal. Electrolyte: CO_2_ purged 0.1 M KHCO_3_, pH ≈6.7. Red shaded area: expected region of adsorbed CO_2_RR intermediate bands. Blue shaded area: expected region of Cu^1+^ bands. Yellow shaded area: expected region of Cu-Histidine complex bands. Dashed lines are a guide to the eye. Raman bands marked at 1009, 1259, 1321, 1485, 1572, and 1640 cm^−1^ can be matched with Raman bands of deprotonated l-histidine adsorbed on Cu in alkaline conditions under applied cathodic bias^24^.

First, we conducted preliminary in-situ Raman to study the adsorption behaviour of histidine under CO_2_RR conditions. For this purpose, a benchmark in-situ Raman experiment was performed on electrodeposited Cu_2_O on Cu substrate in the presence of dissolved 0.025 M histidine in CO_2_-purged 0.1 M KHCO_3_ electrolyte (Fig. 3a, see also SI Section 5.1). At open circuit potential (OCP), bands belonging to Cu_2_O at 519 and 629 cm^−1^ were identified, along with a possible carbonate band near 1073 cm^−1^. A series of weaker bands around 1009, 1155, 1259, 1485, 1572, and 1640 cm^−1^ that are consistent with Raman bands of deprotonated L-histidine adsorbed on Cu in the literature (0.1 M NaOH, −0.6 to −1.0 V vs. Ag/AgCl)^24^ were also observed. As the cathodic potential is applied, the CO_3_^2−^ band at 1073 cm^−1^ disappeared while the histidine-related bands get significantly stronger. Additional bands at 380, 527, 1110, 1321, 1415, and 2079 cm^−1^ appeared, which can also be identified as histidine-related bands (SI Section 5.1). The histidine bands persist up to a highly cathodic potential of −1.1 V. Intriguingly, as the potentials are gradually ramped to more cathodic values, the C ≡ O frustrated rotation and Cu–CO bands (expected around 275 and 356 cm^−1^)^25^ were absent. We identified three bands that are not present in dry histidine samples without electrochemical bias: 1009, 1640, and 2079 cm^−1^ indicating possible new interactions between histidine with either reduced Cu substrate, CO_2_ (or related intermediates), or the electrolyte under applied cathodic biases. The band around 2079 cm^−1^ is particularly interesting, as it is typically assigned to C ≡ O stretching during in-situ Raman on Cu^25^. However, a similarly positioned band was observed on bare Cu and electrodeposited Cu_2_O in the presence of 0.025 M dissolved histidine (Fig. S5.3a, b) and Cu-Hist samples (Fig. S5.3c) in N_2_ purged 0.1 M KHCO_3_. Thus, we ascribe the band around 2079 cm^−1^ to histidine-related interactions with Cu or the electrolyte under cathodic potentials, and not to the typical C ≡ O stretching from adsorbed *CO on Cu (* denotes adsorption site).

The in-situ Raman result on electrodeposited Cu_2_O in presence of dissolved histidine in KHCO_3_ electrolyte is fascinating because it suggests that histidine molecules can adsorb, interact, and persist on catalyst surface during CO_2_RR-relevant potentials. Histidine is a unique molecule that has different forms depending on the degree of protonation. In the bulk electrolyte (CO_2_ saturated 0.1 M KHCO_3_, pH ≈ 6.8), histidine is expected to be in a mixed His^+^/His^±^ state^26^, allowing it to be attracted to the cathode. More importantly, it can react with CO_2_ to form a zwitterion carbamate^27^, allowing the more positively charged imidazole ring to still approach the cathode from the double layer for subsequent interaction. Further explanation of histidine’s attraction towards the cathode and interaction with the Cu surface is described in SI Sections 5.2 and 5.3.

We continued the in-situ Raman investigation on Cu-0 in CO_2_-purged 0.1 M KHCO_3_ electrolyte (Fig. 3b). As expected, Cu-0 behaves just like typical Cu_2_O-derived Cu catalysts^28^, where the Cu_2_O bands disappear almost instantly when −0.10 V cathodic potential was applied. The expected C ≡ O frustrated rotation and Cu-CO bands at 279 and 364 cm^−1^ were also observed clearly once the potential reaches −0.7 V onwards, indicating the suitability of our system to detect the signature of such intermediate species. We note that similar bands are also observed on benchmark measurement on electrodeposited Cu_2_O in CO_2_-purged 0.1 M KHCO_3_ electrolytes (Fig. S5.3d).

We then focused on Cu-Hist as the sample with the best CO_2_RR activity and selectivity in this study. Only Cu_2_O-related bands at 519 and 620 cm^−1^, and broad humps around 1130 cm^−1^ were present on unreduced Cu-Hist at OCP (Fig. 3c). The absence of histidine-related bands at OCP is reasonable, as the initial interaction of histidine on unreduced Cu-Hist is expected to be a mixture of physical and chemical (see Fig. S2.6 and SI Section 5.3 for details). The coverage of chemically bonded histidine on unreduced Cu-Hist was relatively low, with approximately one histidine molecule per 16–26 surface Cu atoms as inferred by XPS (Figs. S2.8 and S2.9). At −0.10 V cathodic voltage, Cu_2_O-related bands immediately disappeared, accompanied by the appearance of CO_3_^2−^ band (1073 cm^−1^) and a series of strong bands that closely matched dissolved histidine experiment and literature values (major: 1009, 1259, 1321, 1485, 1572, 1640, 2081 cm^−1^). These histidine-related bands on Cu-Hist were markedly more intense than the ones observed during dissolved histidine experiments (Fig. 3a), even though the effective histidine concentration in the system should be much lower. The expected Raman bands related to Cu–CO and C ≡ O frustrated rotation (expected around 279 and 364 cm^−1^) were also missing in Cu-Hist, even after ramping the cathodic potential to −1.10 V.

We posit that the initial strong chemical and physical interaction between histidine and Cu_2_O in the unreduced stage through Cu–N bonding (inferred from additional Cu 2*p* peak at higher binding energy and significantly shifted N 1*s* of Cu-Hist sample, Fig. S2.9) may be critical in achieving high surface coverage of histidine during catalysis. We observed superior CO_2_RR on Cu-Hist when compared to physically mixed histidine of similar loading (Fig. S4.6a). The missing Cu–CO and C ≡ O frustrated rotation (expected around 279 and 364 cm^−1^) on Cu-Hist, even at very cathodic potential is intriguing. Given the excellent C_2+_ selectivity on Cu-Hist, the persistent histidine Raman bands and missing M-CO bands at very highly cathodic potentials indicate that strongly adsorbed histidine might have altered the interactions between Cu and *CO (or related intermediates). Previous reports of amino group interactions with CO_2_RR intermediates have been confined only to weak interactions^29^, although altered *CO surface coordination to Ag surfaces in the presence of amino-containing triazole has been reported^30^.

### Theoretical calculations

We then turn to DFT calculations to rationalise the effects of histidine on the selectivity towards CH_4_ and C_2_H_4_ over the Cu-Hist catalyst. A single deprotonated histidine molecule was placed over a 4 × 4 Cu(100) surface slab, based on the observed state and coverage of histidine in our synthesised catalyst from the XPS (SI Section 2.5) and Raman data (Fig. 3c and SI Section 5). Details of the surface model optimisation are described in SI Section 6.2. It is widely accepted that the CO_2_RR on Cu proceeds through a common *CO intermediate^31^. However, our experimental results indicate a possible deviation from the typical *CO–catalyst interaction (absence of Cu–CO and C ≡ O frustrated rotation). Accordingly, we explored an alternative *CO_2_ to *CO conversion with subsequent transformation into CH_4_ and C_2_H_4_ with direct involvement of a histidine molecule. In the following discussion, all intermediates during the reactions are labelled with bold numerals, while each elementary step is represented by A$\to$B, where (A) and (B) are two consecutive intermediates. In the description of reaction intermediates, the “Hist” label refers to the co-adsorbed histidine molecule. We discuss the thermodynamics of the transformation in terms of Gibbs free energies (*G*), and Gibbs free energy change (Δ*G*_A_$\to$_B_).

**Fig. 4:**
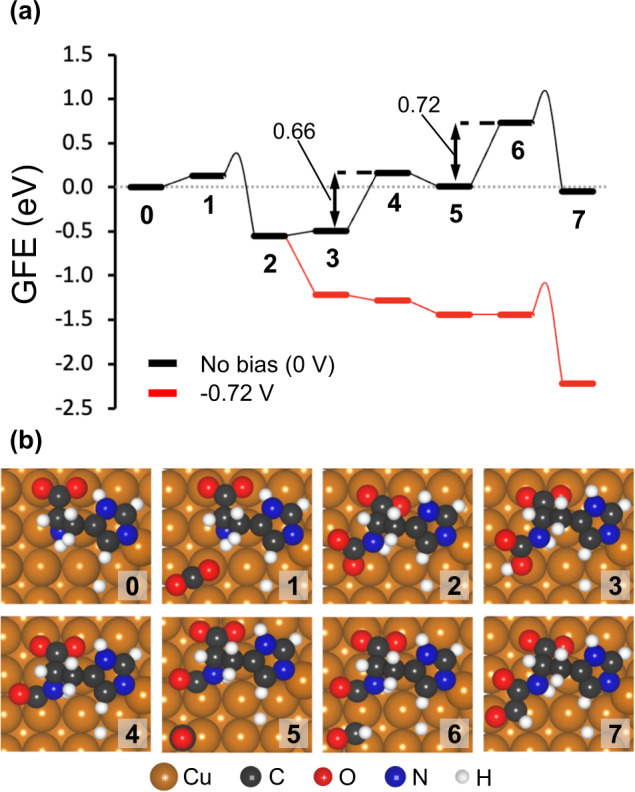
Initial reaction steps during CO_2_RR over histidine-Cu/Cu(100) substrate calculated by DFT. **a** Gibbs free energy (GFE) diagram and the **b** snapshots of the first few surfaces intermediates in the histidine-assisted CO_2_RR mechanism. The GFE diagram was calculated from the reference state (**0**), which consists of a histidine-Cu/Cu(100) shown in Fig. S6.2b, a gas-phase CO_2_ molecule, and an adsorbed *CO. In configurations (1–4), the *CO molecule adsorbed on a bare Cu(100) substrate is omitted for clarity, however, the energy has been added to each system accordingly. This *CO approaches the Hist–CO intermediate (5) and becomes *CHO after a CPET step (5$\to$6), where the thermodynamic barrier Δ*G*_5_$\to$_6_ of 0.72 eV can be overcome with applied bias (red line). The newly formed *CHO species may couple with the co-adsorbed Hist–CO intermediate through a C–C bond to form 7. The intermediates of the surface reactions (1$\to$2 and 6$\to$7) are connected by smooth lines, from which the energy level of the TS may be inferred (the highest point of the smooth lines).

First, a CO_2_ molecule approaches the surface and physisorbs on Cu sites near the deprotonated amine group from histidine (1, Fig. 4). The *CO_2_ adsorbate may bind the N atom in histidine (1$\to$2) by overcoming a barrier of 0.23 eV to form the Hist–CO_2_ complex (2). The C–N coupling is highly exergonic with Δ*G*_**1**→**2**_ of −0.68 eV, indicating a thermodynamically favoured product. The electrochemical conversion of Hist–CO_2_ to Hist–CO involves two coupled proton–electron transfer (CPET) steps. The first CPET forms Hist–COOH (3) in a slightly endergonic process (Δ*G*_2→3_ = 0.05 eV), while a subsequent CPET generates H_2_O and Hist–CO intermediate (4) on the surface (Δ*G*_3→4_ = 0.66 eV). From here, a surface *CO, originally on Cu sites distant from the histidine molecule, approaches to the Hist–CO intermediate (5). The free energy change (Δ*G*_4→5_ = −0.16 eV) suggests that the approach of *CO to sites near Hist–CO may occur spontaneously.

A following CPET would transform the *CO into *CHO (6) with Δ*G*_5→6_ of 0.72 eV, which is the most endergonic step where a modest applied potential should be applied to make the reaction proceed (Fig. 4a, red curve). Once the generated *CHO (thermodynamically favoured over *COH on Cu(100)^32^) is present around the Hist–CO intermediate, the C–C coupling between the histidine-bound *CO and *CHO (6$\to$7, Fig. 4b) is both kinetically (*E*_a_ = 0.33 eV) and thermodynamically (Δ*G*_3→4_ = −0.78 eV) more favourable than the baseline cases (*CO–*CO and *CO–*CHO coupling on Cu(100) surface, SI Section 6.8). In our calculations, the *CO–*CO coupling over Cu(100) is endergonic by 0.96 eV and has a free energy barrier of 1.31 eV (Fig. S6.8), while the coupling between *CO and *CHO is endergonic by 0.10 eV and has a barrier of 0.63 eV.

**Fig. 5:**
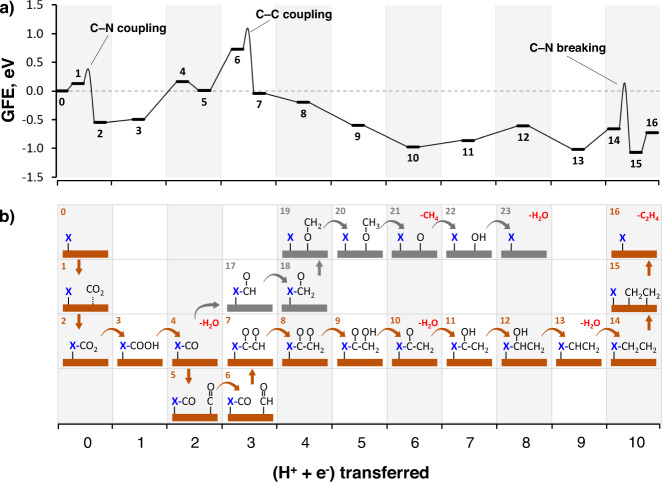
Mechanistic understanding of favourable and unfavourable CO_2_RR pathways on Cu-Hist calculated by DFT. **a** Gibbs free energy diagram for CO_2_ to C_2_H_4_ through the histidine-assisted mechanism. Configuration (0) is a reference configuration consisting of a histidine-Cu/Cu(100) (Fig. S6.2b), a gas-phase CO_2_, and an adsorbed *CO. The intermediates participating in surface reactions (i.e., C–C coupling and C–N breaking steps) are indicated at the same abscissa and are linked by a curve illustrating the energy barrier of the process. The effect of applied bias on the free energy of this transformation is shown in Fig. S6.3. **b** Reaction pathways for the histidine-assisted electroreduction of CO_2_ to CH_4_ and C_2_H_4_ on Cu(100). Histidine molecule is represented by a blue “X”. Note that the reaction may follow two separate pathways from (4), through either (5) towards C_2_H_4_ (marked with orange substrates) or (17) towards CH_4_ (marked with grey substrates). Details on the histidine-assisted formation of CH_4_ are discussed in SI Section 6.7. Desorbed molecules during the chemical process are indicated in red. The number at the top left corner of each box represents the reaction step number as described in the main text.

Apart from the *CO and *CHO adsorbed on the surface, other C–C coupling alternatives from (5) were also explored (e.g., coupling between Hist-CO and *CO or Hist-CHO and *CO). However, the resulting activation energies were found to be considerably higher (1.13 and 1.43 eV, SI Section S6.9) than the (6 → 7) coupling. In addition, once the Hist-CO-CHO (7) is formed, it may be transformed into Hist-COH-CHO, Hist-CHO-CHO, Hist-CO-CHOH, or Hist-CO-CH_2_O during the following CPET (SI Section S6.9). We found that the reaction is most likely to proceed via Hist-CO-CH_2_O (8, Fig. 5a) due to comparatively higher thermodynamic barriers in other reaction channels.

In Fig. 5a, we can see all CPET steps following (7) are not potential limiting, as most of them are downhill steps and the Δ*G* for uphill steps (11$\to$12) and (13$\to$14) are <0.72 eV (Δ*G*_5→6_). After the Hist–CH_2_CH_2_ intermediate (14) is formed, it needs to be decoupled from the histidine fragment to complete the whole reaction. This step requires overcoming an energy barrier of 0.79 eV to break the C–N bond (14$\to$15). However, this C–N bond cleavage (14$\to$15) has a much lower energy level compared to the C–C bond coupling (6$\to$7) (Fig. 5a), thus it should not be rate limiting_._ We found that the Hist–CO and *CHO coupling (6$\to$7) has the highest energy level in the energy profile, which is the key step at 0 V (Fig. 5a). However, the CO_2_ binding with Cu-Hist (1$\to$2) becomes more important when a potential of −0.72 V is applied (Fig. S6.3).

As CH_4_ formation is suppressed on Cu–Hist, a reaction pathway producing methane was also studied from (4) (Fig. 5b, grey shaded substrate and SI section S6.7) to understand the reason behind this. We found this pathway requires a surface reaction (C–N bond-breaking, 18$\to$19) to release CH_2_O from adsorbed histidine for the subsequent C protonation to produce CH_4_. This step has an energy barrier of 0.99 eV, making it the rate-limiting step in the formation of CH_4_ on Cu-Hist, and higher than the barrier of 0.57 eV for the rate-limiting (*CO$\to$*CHO) on bare Cu (100) (Fig. S6.6).

Ergo, the presence of histidine plays opposite roles for C_2_H_4_ and CH_4_ formation. In addition, the limited availability of H^+^ near the surface (due to the expected basic pH in the double layer) and the increased concentration of *CO species near histidine may also account for the more favourable C_2_ product pathway.

This alternative mechanism for CO_2_RR via histidine-assisted transformations may help rationalise the absence of the C ≡ O frustrated rotation in the Raman bands at applied bias during our experiments. On the one hand, *CO_2_ may be transformed into *CO while bound to the histidine molecule through the amine N atom (1$\to$2$\to$3$\to$4), limiting the interaction of *CO with the Cu surface sites. On the other hand, the estimated high surface coverage of histidine (1 molecule per 16–26 surface Cu) may limit the amount of surface *CO bound to Cu sites, considerably affecting the intensity of characteristic Raman bands. In addition, the resulting few Cu–*CO intermediates may transform quickly into *CHO at high negative potentials (<−0.72 V) according to the Boltzmann probability distributions (SI Section 6.5 and Fig. S6.4), reducing further the measurable Cu–*CO indicators.

### Electrochemical surface charge investigations

From in-situ Raman and DFT investigations, we learnt that histidine remains specifically adsorbed on Cu-Hist surface during CO_2_RR and can provide alternative pathways towards C_2+_ via the formation of Hist-CO intermediate. The accumulation of such surface-adsorbed species can change the local electric field and provide additional electrostatic interaction near the catalyst surface, akin to the cation effect reported previously^33–35^. Therefore, we enlist transient electrochemical techniques in an attempt to quantify the changes in the surface charge. At the same time, we posit that the metrics obtained by transient electrochemical techniques may also be exploited as “activity descriptors” to predict CO_2_RR activity over a wide range of catalyst systems.

**Fig. 6:**
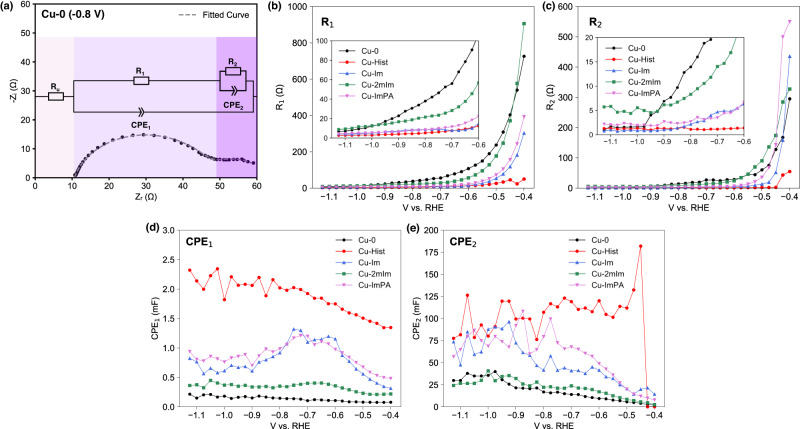
Evaluation of organic functionalised Cu catalysts using EIS measurement at catalytically relevant DC potentials. **a** The equivalent circuit used for EIS spectra fitting. Numerical data fitting results of EIS spectra measured on five samples across different DC potentials: **b** *R*_1_, **c** *R*_2_, **d** CPE_1_, and **e** CPE_2_.

We first turn to EIS as the established technique capable of probing time-dependent reaction mechanisms^36^, from which *R*_CT_ and capacitance values can be extracted and linked to catalytic activity. However, EIS measurements in literature are often performed at potentials irrelevant to the catalysis process, assuming that the reaction progression is governed by electron transfer kinetics^37^. Therefore, we performed EIS across a wide cathodic potential range from −0.400 to −1.125 V under CO_2_RR conditions (CO_2_ purged 1 M KHCO_3_). A modified Randle’s circuit model that accounts for two possible electrochemical interfaces with different timescales was used (Fig. 6a), in line with the observation of a second semicircle at lower frequencies and more cathodic potentials (SI Section 7)^37,38^. The extracted *R*_CT_ values at different time domains (labelled *R*_1_ and *R*_2_) were then compared across a wide applied cathodic voltage. Here, *R*_1_ corresponds to the high-frequency component (>50 Hz, Fig. 6b) and *R*_2_ the low-frequency (<50 Hz, Fig. 6c). *R*_1_ is frequently attributed to the double layer component, while *R*_2_ is assigned to the slower Faradaic or pseudocapacitive element due to the more relevant timescale^38,39^. Both *R*_1_ and *R*_2_ change inversely to applied cathodic voltage and converge to similar values on all samples, indicating that electron transfer pathways are generally facile during CO_2_RR. Functionalised samples do show lower *R* values compared to Cu-0, but the differences are generally very small (<9 Ω for *R*_1_ and <5 Ω for *R*_2_) once the applied potential reaches CO_2_RR relevant potentials (<−1.0 V).

The capacitive terms were also assessed, as they can be closely related to accumulated species at or near the cathode surface^36^. This term is represented by a constant-phase element (CPE) reflecting the frequency dispersion behaviour of the solid-electrolyte interface^39^. Like the R terms, the CPE component consists of CPE_1_ and CPE_2_ representing the higher and lower frequency regions respectively. A clearer divergence between the CPE values of functionalised and unfunctionalized surfaces was prevalent—the most active Cu-Hist displaying ≈13.9× and ≈5.5× higher average CPE_1_ and CPE_2_, respectively, than Cu-0 (*V* < −1.0 V), an indication of higher population of charged species in the double layer or near catalyst surface at the onset of CO_2_RR that may be related to the adsorbed histidine and intermediates.

At first glance, the general trend of *R*_1_, CPE_1_, and CPE_2_ values at CO_2_RR relevant potentials of −1.000 to −1.125 V appears to be in line with the observed CO_2_RR trend (Fig. S9.1). However, large fluctuations at more cathodic potentials may originate from the momentary changes in capacitance values arising from vigorous gas evolution (Fig. 6d, e), hindering data collection for potentials more cathodic than −1.125 V due to vigorous bubbling (Fig. S7.3). Hence, we opine that EIS-derived metrics may not be suitable to describe CO_2_RR activity due to large errors at catalytically relevant potentials.

In search of a better way to measure surface charge, we turn to a modified pulsed voltammetry (mPV) technique (see SI Section 8 for details). The mPV excitation pulse was constructed with a fixed upper bound near the OCP (typically around +0.2 to +0.3 V) and gradually more cathodic lower bound voltage between around −0.5 to −1.6 V which is relevant to the CO_2_RR operating voltage of our catalysts. The applied mPV would trigger a repeating cycle of charged species accumulation (adsorption) during cathodic lower bound voltage and decumulation (desorption) at OCP. Like EIS and other electrochemical techniques^36,40–42^, we recognise that mPV alone cannot ascertain the identity of adsorbed intermediates nor their adsorption behaviour. However, it serves to provide an estimate of the relative quantity and desorption kinetics of such species when the applied cathodic potential is removed.

**Fig. 7:**
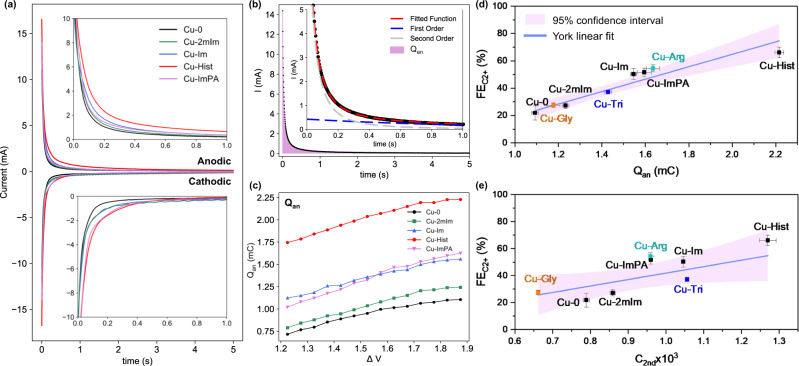
Evaluation of organic functionalised Cu catalysts using mPV measurement at catalysis-relevant DC potentials. **a** The transient current responses of Cu-0 and four organic functionalised samples at ∆*V* = 1.8 V, showing transient anodic and cathodic current responses immediately after the applied voltage pulse. **b** Detailed view of transient anodic pulse for Cu-0 at ∆*V* = 1.8. Shaded area represents the integrated transient anodic current decay (*Q*_an_). Broken blue and grey lines represent the 1st and 2nd order decay equation fitting respectively, and the composite function is represented by broken red lines. Insets in **a** and **b** show enlarged portion of the current decays up to the first second. **c** integrated *Q*_an_ value of Cu-0, Cu-2mIm, Cu-Im, Cu-Hist, and Cu-ImPA with increasing ∆*V*. **d**–**f** Correlation plot between average **d** Q_an_, **e** C_2nd_ and the corresponding C_2+_ product selectivity (FE_C2+_) at −1.6 V. Orange, blue and turquoise coloured data points are additional validation points of Cu-Gly, Cu-Tri, and Cu–Arg. Error bars represent the standard deviation of three independent measurements. Solid purple lines and shaded areas in **d** and **e** represent York linear fit (considers both *x*- and *y*-errors), and the 95% confidence interval band, respectively.

Overall, the transient anodic and cathodic responses appear to grow with more cathodic lower bound on all samples (Fig. S8.1), indicative of higher surface charge density at more cathodic potentials as expected. The magnitudes of anodic and cathodic pulse decays of all samples at ∆*V* = 1.8 V (approx. −1.5 to −1.6 V) are roughly balanced after subtracting the steady-state catalytic current (Fig. 7a), suggesting the reversibility of the charges. Due to the convolution of the catalytic current with the cathodic decay profile, the anodic decay profile was instead selected for further analysis.

We start by integrating the anodic transient response for 5 s (Fig. 7b). The integrated anodic transient charge (*Q*_an_) grows almost monotonously with more cathodic lower voltage bound on all samples (Fig. 7c). This suggests a gradual build-up of charged species concentration on the catalyst surface with increasingly cathodic potentials. The rate of *Q*_an_ growth with respect to ∆*V* was similar among the different samples, and the *Q*_an_ trend of Cu-Hist > Cu-Im ≈ Cu-ImPA > Cu-2mIm > Cu-0 was practically maintained throughout the entire ∆*V* range. Further mathematical fitting and analyses (SI Section 8.1) found that the anodic transient current decay cannot be fitted to just one decay model commonly observed in electrochemical systems^43^. Instead, the best fit can be obtained with a combination of second-order and first-order decay kinetic functions (Fig. 7b), suggesting a convolution of at least two surface processes:

$$I={k}_{2{{\rm {nd}}}}{\left(\frac{1}{{C}_{2{{\rm {nd}}}}}+{k}_{2{{\rm {nd}}}}t\right)}^{-2}+{C}_{1{{\rm {st}}}}{k}_{1{{\rm {st}}}}{{\rm {e}}}^{-{k}_{1{{\rm {st}}}}t}$$

The second-order decay kinetic dominates early up to *t* ≈ 0.4–0.5 s, which is ascribed to the cumulative desorption of species on the catalyst surface. The first-order decay term dominates at the longer timescale of *t* > 0.5 s, representing slow background processes occurring near OCP, such as Cu re-oxidation (SI Section 8.2). We focus on the second-order term and extract the *C*_2nd_ and *k*_2nd_ coefficients representing the initial surface charge accumulation and desorption rate respectively.

The extracted *C*_2nd_ values trend follows: Cu-Hist > Cu-Im > Cu-ImPA > Cu-2mIm > Cu-0, which is in the same order as the CO_2_RR activity to C_2+_ product. Meanwhile, *k*_2nd_ trend is inversed (Cu-0 > Cu-2mIm ≈ Cu-ImPA > Cu-Im ≥ Cu-Hist). Thus, higher CO_2_RR catalytic activity is linked to higher initial surface charge accumulation (high *C*_2nd_) and slower desorption (low *k*_2nd_), which leads us to hypothesise that superior CO_2_RR performance to C_2+_ product is related to better intermediate stabilisation aided by the organic functionalisations. We found that the trend of mPV derived parameters (*Q*_an_, *C*_2nd_, and *k*_2nd_) are still in line with the C_2+_ product selectivity trend, even with the inclusion of other organic molecules where the imidazole group are absent, namely arginine, glycine, and 1,2,3-triazole (Cu-Arg, Cu-Gly, Cu-Tri, Figs. S4.7, S7.4, and S8.6). Ergo, this correlation suggests that mPV may be used to predict C_2+_ selectivity on wider types of catalyst surfaces.

Overall, both EIS and mPV measurements reflect elevated surface charge on organic-functionalised surfaces in the same order of CO_2_RR to C_2+_ activity compared to bare Cu-0. Among all parameters derived from EIS and mPV, *Q*_an_ stands out as a simple yet elegant choice as a catalytic activity descriptor, as a more accurate integration of *Q*_an_ can be obtained across different samples at CO2RR-relevant potentials. The correlation between *Q*_an_ and FE_C2+_ (Fig. 7d, also *j*_C2+_ in Fig. S8.2b) predicts that catalysts with a higher population of charged species on the surface potentially produce more C_2+_ products, which may be related to the effectiveness of the surface functionalisation in stabilising CO_2_RR intermediates. We speculate that the concentration of charged species on the catalyst surface during CO_2_RR may be more important than the stability of these species at OCP, as inferred from the better correlation of *C*_2nd_ with FE_C2+_ compared to *k*_2nd_ (Fig. 7e, also *j*_C2+_ in Fig. S8.2d).

Parallels can be drawn between our results that show clear CO_2_RR activity modulation in presence of histidine to the cation effect on CO_2_RR selectivity^33–35^. A major difference between the cation effect and our Cu-Hist is that histidine can be specifically adsorbed on Cu_2_O-derived Cu surface under cathodic bias, as shown by the in-situ Raman data (Figs. 3 and S5.3). Further, the hydration shell of histidine (and possibly other amino acids) is much larger than alkali cations, but softer^44^, thus enabling higher surface charge accumulation than Cs^+^ cation while allowing for specific adsorption to reduced Cu surface. In addition, much stronger interaction with intermediate is afforded on Cu-Hist through the amine group, unlike cations where only electrostatic effects are available^35^. To increase the surface charge, we speculate that the amino acids could be modified by placing electron-donating groups on the amino nitrogen to increase basicity, whilst balancing the interaction with *CO or *CHO intermediate coupling. One could also combine the effect of cation together by swapping K^+^ with Cs^+^ which may allow further stabilisation of the intermediate through additional electrostatic effect.

Our work highlights the effectiveness of organic surface functionalisation in enhancing the CO_2_RR activity and tuning the selectivity of transition metal catalysts. Particularly, histidine’s unique combination of functional groups allows for the stabilisation of CO_2_RR intermediates and robust anchoring to the catalyst surface. When combined with Cu_2_O-derived Cu, Cu-Hist sample displays significantly higher C_2+_ across a very wide voltage range of >1 V, with up to 76.6% FE_C2+_, with its overall performance and stability outperforming recent C_2+_-selective catalysts (SI Section 10). In-situ Raman underlines strong interaction between histidine and Cu that persists at CO_2_RR relevant potentials, which may stem from the close interaction between Cu_2_O and histidine in the catalyst precursor before reduction. The stabilised histidine on the Cu surface appears to allow alternative CO_2_RR pathways through direct interaction of *CO_2_ with histidine, as shown by DFT calculation. Under negative potentials, the formation of C_2+_ products is preferred over methane on the Cu-Hist catalyst due to lower energy barriers in the key reaction steps.

More interestingly, we discover that the stabilised organic functional groups increased the surface charge near the catalyst surface, as determined by EIS and mPV measurements. While the surface charge modulation effects may be similar to the cation effect, the organic functional groups employed in our case are likely to be specifically adsorbed, providing a more stable and potent means to boost CO_2_RR selectivity by opening new pathways for intermediates stabilisation.

We also found that the CO_2_RR enhancement of the organic-functionalised catalyst is highly correlated to the catalyst surface charge measured using the mPV method. Additional measurements on seven other molecules demonstrate that such correlations can be generalised beyond histidine and imidazole-containing functionalisation. Our results highlight the potential of surface charge measurements as a powerful addition to the characterisation arsenal for electrocatalytic activity^45^. With further validation works on other catalytic systems and creating more accurate regression models, we expect surface charge measurements to be a very useful proxy for future catalyst discovery, including organic–inorganic hybrids.

## Methods

Functionalised Cu_2_O was synthesised using a simple and scalable wet synthesis method. In brief, stoichiometric amounts of d-glucose (5 mmol, 0.90 g), anhydrous copper chloride (10 mmol, 1.34 g), and 1.5 mol.% (0.15 mmol) of the molecule of interest were dissolved in 50 mL of deionised water. The solution was heated to 75 °C in a water bath and hydroxide ions were introduced into the reaction mixture in excess via the dropwise addition and 20 mL of 2 M sodium hydroxide solution. The reaction mixture was left to stir for 1 h and the mixture was subsequently centrifuged at 6000 rpm (4830×*g*) for 4 min. The precipitate was washed and dried overnight at 60 °C in a vacuum oven. More details on materials and synthesis are described in SI Section 1.

XPS data were acquired at HarwellXPS. A Kratos Axis SUPRA was used, employing monochromatized Al kα (1486.69 eV) X-rays at 15 mA emission and 12 kV HT (180 W) and a spot size/analysis area of 700 × 300 µm. Gas cluster ion source is employed to perform the depth profile of the Cu-Hist. Details on XPS measurements are presented in SI Section 2.5. Additional materials characterisations are presented in SI Section 2.

CO_2_RR experiments were performed in a custom electrochemical H-cell made of PEEK and PTFE. The electrolyte compartments were separated by an anion exchange membrane and each electrolyte compartment was filled with 8 mL of electrolyte. Gas products were quantified using gas chromatography and liquid products were quantified using ^1^H NMR. Additional H-cell measurements, including baseline, control experiments with physically mixed histidine–Cu_2_O or glassy carbon, and calibration data are presented in SI Section 3.

An open cell without a membrane was used for mPV and EIS measurements. Electrochemical impedance spectroscopy (EIS) data were collected from pre-reduced catalysts with 1 M KHCO_3_, with higher electrolyte concentration used to minimise the *R*_u_ as well as create a more stable EIS environment. The spectrums were then collected across 30 different potentials between the range of −0.400 to −1.125 V vs. RHE at 0.025 V increments. The spectra were collected from 0.5 Hz to 30 kHz at 10 data points per decade on a Gamry 600+ potentiostat. The obtained Nyquist plots were individually fitted using the Simplex method on Gamry Echem Analyst (v7.9.0). Detailed experimental data of EIS experiments are presented in SI Section 4.

Raman spectroscopy was performed with a confocal Raman microscope (LabRAM HR Evolution, Horiba Jobin Yvon) in an epi-illumination mode (top-down) with He–Ne laser (633 nm, Pacific Lasertech) excitation source. A water immersion objective lens (LOMO APO water phase, ×70, numerical aperture: 1.23) protected by a 0.013 mm thin Teflon film was used to collect spectra. A custom-made PTFE open cell filled with CO_2_ (or N_2_) purged 0.1 M KHCO_3_ was used to hold 10 mm electrode and perform the in-situ electrochemistry, controlled by a Gamry 600 potentiostat. Gas purging is maintained during measurement. More details on the in-situ Raman and additional measurements are presented in SI Section 5.

The DFT calculations were carried out with the projector augmented wave method (PAW)^46^ and the PBE functional^47^ with D3 dispersion corrections^48^ as implemented in the VASP software^49–51^. An energy cut-off of 400 eV was used for the plane waves and the occupancies were treated with a Gaussian smearing technique with a width of 0.05 eV, but eventually extrapolated to zero width. We consider that a self-consistent field (SCF) cycle was converged when the change in energy was lower than 1 × 10^−5^ eV, while the atomic optimisations were stopped when all forces were below 0.02 eV Å^−1^. All surface calculations were done with a 4 × 4 Cu(100) surface slab cell where the Brillouin zone was sampled with a 4 × 4 × 1 Monkhorst–Pack mesh^52^. The NEB method^53^ (and its climbing-image modification^54^) was used to locate the transition states and the finite differences method was used to approximate the harmonic vibrational frequencies. The Gibbs free energy for each intermediate and transition state was corrected with the corresponding terms (zero-point energy, thermal and entropy effects), including solvation. An extended description of the computational details, the surface model used in this work, energy corrections, and alternative pathways comparisons are presented in SI Section 6.

mPV experiments were conducted in 0.1 M KHCO_3_, and the catalysts were pre-reduced at −1.125 V for 2000 s. The anodic potential was set around OCP, typically around +0.3 V, where Faradaic processes were minimal. The cathodic potential is varied at 0.05 V intervals, with Δ*V* values (Δ*V* = *V*_anodic_–*V*_cathodic_) ranging from 0.775 to 1.875 V. Both anodic and cathodic pulses were applied for 20 s and the sampling time of the current was 0.004 s. The anodic current decays were integrated from *t* = 0 s to *t* = 5 s using scipy.integrate.simps while mathematical fitting of the pulse from *t* = 0 s to *t* = 2 s was conducted using the scipy.optimise.curve_fit function with positive bounds. Detailed experimental data of EIS, mPV, and all parameter correlations are presented in SI Section 7–9.

CO_2_RR activity, selectivity and stability performance benchmarking with literature is presented in SI Section 10.

## Supplementary information

Supplementary information

Peer review file

## Data Availability

The authors declare that all data supporting the results of this study are available within the paper and its supplementary information files. Product quantification, EIS, modified pulse voltammetry and variable rate cyclic voltammetry data is available from Figshare database under accession code (10.6084/m9.figshare.21787079).

## Code availability

Modified Pulse Voltammetry data treatment code is available at Figshare database under accession code (10.6084/m9.figshare.21787079).
