# Author Correction: Surface charge as activity descriptors for electrochemical CO_2_ reduction to multi-carbon products on organic-functionalised Cu

**DOI:** 10.1038/s41467-023-39852-0

**Published:** 2023-07-10

**Authors:** Carina Yi Jing Lim, Meltem Yilmaz, Juan Manuel Arce-Ramos, Albertus D. Handoko, Wei Jie Teh, Yuangang Zheng, Zi Hui Jonathan Khoo, Ming Lin, Mark Isaacs, Teck Lip Dexter Tam, Yang Bai, Chee Koon Ng, Boon Siang Yeo, Gopinathan Sankar, Ivan P. Parkin, Kedar Hippalgaonkar, Michael B. Sullivan, Jia Zhang, Yee-Fun Lim

**Affiliations:** 1 Institute of Materials Research and Engineering, Agency for Science, Technology and Research (A*STAR), 2 Fusionopolis Way, Innovis, Singapore, 138634 Singapore; 2 School of Materials Science and Engineering, Nanyang Technological University, 50 Nanyang Avenue, Singapore, 639798 Singapore; 3 Department of Chemistry, University College London, 20 Gordon Street, London, WC1H 0AJ UK; 4 Institute of High Performance Computing, Agency for Science, Technology and Research (A*STAR), 1 Fusionopolis Way, Connexis, Singapore, 138632 Singapore; 5 Department of Chemistry, National University of Singapore, 3 Science Drive 3, Singapore, 117543 Singapore; 6 Research Complex at Harwell, Rutherford Appleton Laboratory, Harwell Science and Innovation Campus, Didcot, Oxfordshire OX11 0FA UK; 7 Institute of Sustainability for Chemical, Engineering and Environment, Agency of Science, Technology and Research (A*STAR), 1 Pesek Road, Singapore, 627833 Singapore

**Keywords:** Electrocatalysis, Electrocatalysis, Electrochemistry

Correction to: *Nature Communications* 10.1038/s41467-023-35912-7, published online 20 January 2023

The original version of this Article contained an error in Fig. 6e, in which Fig. 6e was mistakenly given as a duplicate of Fig. 6d. This has been corrected in both the PDF and HTML versions of the Article.

