# Supplementary material for: Surface charge as activity descriptors for electrochemical CO2 reduction to multi-carbon products on organic-functionalised Cu
Source: Nat Commun. 2023 Jan 20;14:335. doi: 10.1038/s41467-023-35912-7 (PMC9860078; doi:10.1038/s41467-023-35912-7)
Supplement: Supplementary file 1 — Supplementary information [file 41467_2023_35912_MOESM1_ESM.pdf]

Supporting Information

Surface Charge as Activity Descriptors for Electrochemical CO_2 Reduction to Multi-Carbon Products on Organic-Functionalised Cu

Carina Yi Jing Lim [a,b,†], Meltem Yilmaz [a,c,†], Juan Manuel Arce-Ramos[d,†], Albertus D. Handoko[a,*,†], Wei Jie Teh[e], Yuangang Zheng[a], Zi Hui Jonathan Khoo[a], Ming Lin[a], Mark Isaac[f], Teck Lip Dexter Tam[g], Yang Bai[a], Chee Koon Ng[a], Boon Siang Yeo[e], Gopinathan Sankar[c], Ivan P. Parkin[c], Kedar Hippalgaonkar [a,b], Michael B. Sullivan[d], Jia Zhang[d,*], Yee-Fun Lim [a,g,*]

[a]Institute of Materials Research and Engineering, Agency for Science, Technology and Research (A*STAR), 2 Fusionopolis Way, Innovis, Singapore 138634, Singapore

[b]School of Materials Science and Engineering, Nanyang Technological University, 50 Nanyang Avenue, Singapore 639798, Singapore

[c]Department of Chemistry, University College London, 20 Gordon Street, London, WC1H 0AJ, United Kingdom

[d]Institute of High Performance Computing, Agency for Science, Technology and Research (A*STAR), 1 Fusionopolis Way, Connexis, Singapore 138632, Singapore

[e]Department of Chemistry, National University of Singapore, 3 Science Drive 3, Singapore 117543, Singapore

[f]Research Complex at Harwell, Rutherford Appleton Laboratory, Harwell Science and Innovation Campus, Didcot, Oxfordshire OX11 0FA, United Kingdom

[g]Institute of Sustainability for Chemical, Engineering and Environment, Agency of Science, Technology and Research (A*STAR), 1 Pesek Road, Singapore 627833, Singapore.

[†]These authors contributed equally to this work.

[*]Email: adhandoko@imre.a-star.edu.sg, zhangj@ihpc.a-star.edu.sg, limyf@imre.a-star.edu.sg

Table of Contents

1. Materials and Synthesis 4
   1.1. Materials 4
   1.2. Synthesis of Cu_2O and Functionalised Cu_2O 4
   1.3. Molecules of Interest 4
2. Materials Characterization 6
   2.1. X-ray Diffraction 6
   2.2. Scanning Electron Microscopy 7
   2.3. Transmission Electron Microscopy 8
   2.4. Ex-situ Raman Spectroscopy and Fourier Transform Infrared Spectroscopy (FTIR) 10
   2.5. *Ex-situ* X-ray Photoelectron Spectroscopy 12
   2.6. Additional CHNS Elemental Analysis 14
3. Electrochemical Methods 15
   3.1. Electrode Preparation 15
   3.2. Electrochemical CO_2 Reduction Experiments 15
   3.3. Turnover Frequency Calculation 16
   3.4. Electrochemically Active Surface Area Measurement 18
4. H-cell measurements, Product Quantification, Control Measurements, and Calibration 19
   4.1. Optimization of Functionalisation Loading 19
   4.2. Baseline Experiments Under Argon Flow 20
   4.3. Calibration of Gas Chromatograph 21
   4.4. Calibration of NMR 22
   4.5. Partial Current Densities of H_2 and C_{2+} Products 22
   4.6. Additional H-Cell Control Experiments with Glassy Carbon Substrate and Cu_2O with Physically Mixed 2% Histidine 23
   4.7. Comparison of Catalytic Performance at -1.6 V between Cu_2O with Additional Functional Groups24
5. In-situ Raman Spectroscopy 25
   5.1. Supplementary In-situ Raman Measurements 27
   5.2. Explanation on the attraction of histidine towards cathode upon electrochemical bias 28
   5.3. Explanation on histidine-Cu interaction during CO_2RR 28
6. Density Functional Theory Calculations 30
   6.1. Details of the computational methods 30
   6.2. Surface model 31
   6.3. Correction to CO gas-phase molecule 33

6.4. Effect of applied bias on CO_2RR 34
6.5. Boltzmann probability distributions of *CHO and *CO surface species 34
6.6. Comparison of other solvation methods 35
6.7. Other pathways explored 37
6.8. C-C coupling alternatives 39
6.9. Alternative pathways during the histidine-assisted hydrogenation of CO_2 40
7. Electrochemical Impedance Spectroscopy 41
8. Modified Pulse Voltammetry (mPV) 44
8.1. Representative Plots of Fitted Anodic Current Decay with Various Functions 45
8.2. Plots of All Fitted Anodic Current Decays with Combined Function 48
9. Parameter Correlations 51
10. Performance Benchmarking 53
11. References 54

1. Materials and Synthesis

1.1. Materials

Copper chloride (99.995%), D-glucose (99.5%), L-histidine (≥99%), imidazole (99%), 2-methylimidazole (≥98.5 %), imidazolepropionic acid (≥98%), and sodium hydroxide (97.5%) was purchased from Sigma Aldrich. Emsure Potassium Hydrogen Carbonate, 99.7 to 100.5% Assay, ACS Grade was used for the electrolyte. Purelab Option-Q, 18.2 M cm deionized water and absolute grade ethanol from VWR Chemicals were used. Perfluorinated resin solution containing Nafion of 5 wt.% Nafion in lower aliphatic alcohols and water purchased from Sigma Aldrich was used in the catalyst ink. Vitreous carbon discs (15 mm diameter, 1 mm thick) purchased from Goodfellow Cambridge Ltd. AMVN anionic exchange membrane was purchased from AGC Engineering Co. Ltd.

1.2. Synthesis of Cu_2O and Functionalised Cu_2O

Stoichiometric amounts of D-glucose (5 mmol, 0.90 g), anhydrous copper chloride (10 mmol, 1.34 g) and 1.5 mol.% (0.15 mmol) of the molecule of interest (Hist, Im, 2-mIm, ImPA, Tri, Arg) were dissolved in 50 mL of deionised water. The solution was heated to 75 °C in a water bath and hydroxide ions were introduced into the reaction mixture in excess via the dropwise addition and 20 mL of 2 M sodium hydroxide solution. The reaction mixture was left to stir for 1 h and the mixture was subsequently centrifuged at 6000 rpm (4830 ×g) for 4 mins. The precipitate was washed thrice with deionized water and ethanol with centrifugation, before drying overnight at 60 °C in a vacuum oven.

1.3. Molecules of Interest

Imidazole (Im) and the related 2-methylimidazole (2-mIm) are selected as model functional group because their structural similarity and presence of the imidazole ring that has been shown to promote CO_2RR.[1,2] The difference between Im and 2-mIm lies in the availability of the C-2 carbon, which is available on Im but blocked on 2-mIm. Imidazole propionic acid (ImPA) is added as a further comparison molecule, as it has very similar structure with histidine, including the carboxylic end group, but without the amino nitrogen. Some of these molecules have been used in the literature for enhancing CO_2RR. The most common reasonings used to explain the enhancement is the stabilisation of $*CO_2$ on the catalyst surface,[1,3] or co-catalytic function of the imidazole molecule related to bound CO_2 to C-2 of the imidazole (parallel to the observation for imidazolium compounds[3]). These C-2 possibly facilitates the formation of adsorbed $*CO_2^-$ anion that allows a more efficient sequential proton-electron transfer (SPET).[4]

In main text we show how Cu_2O-derived Cu (Cu-0) itself without surface functionalisation display very similar CO_2RR selectivity with literature.[5] However, it is clear that by simple inclusion of imidazole-related surface functionalisation is able to boost the C_2 product (ethylene and ethanol) in the expense of methane. C_2 products can still be detected across very wide potential window that outperforms unfunctionalized Cu_2O-derived Cu, up to 2.2 V RHE.

Arginine (Arg), Glycine (Gly), were selected as validation molecules as these molecules do not contain imidazole/-ium functionality, but still contain carboxylic and N group that can bind to Cu. Triazole (Tri) was added to check the importance of carboxylic anchor group and C-2 carbon. The schematics of these molecules of interest are shown in **Figure S1.1**.

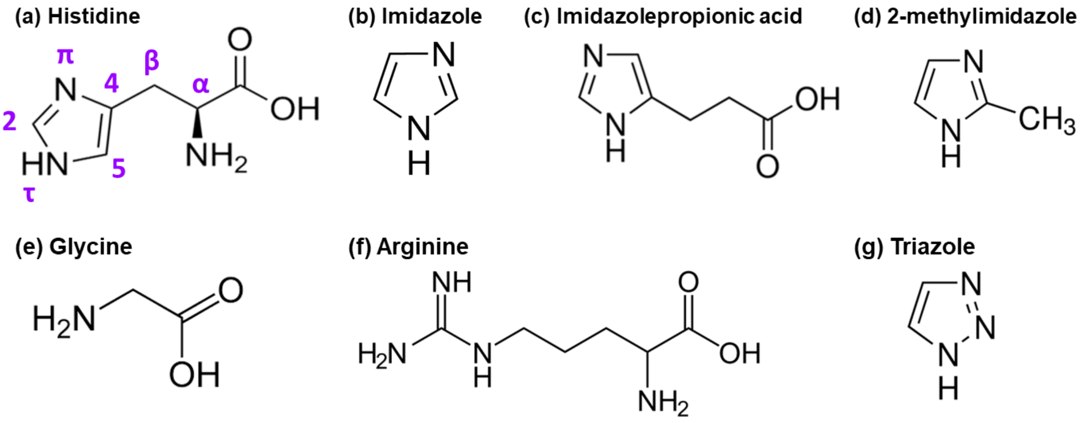

Figure S1.1: The schematics of molecules of interest used in this work as organic functionalisation to Cu. (a) Histidine. The carbon numberings as per IUPAC convention is indicated. (b) Imidazole. (c) Imidazolepropionic acid. (d) 2-methylimidazole. (e) Glycine. (f) Arginine. (g) Triazole.

2. Materials Characterization

2.1. X-ray Diffraction

X-ray diffraction (XRD) was performed on a Bruker Advance D8 diffractometer (Cu Kα λ = 1.541058 Å) equipped with Ni filter and LynxEye XE energy dispersive 1-D detector over a range of 10 – 85° 2θ with a step size of 0.02° and collection time of 0.5 s per step. A variable divergence slit programmed at 10 mm irradiated sample length was used to enhance the diffracted reflections at the higher 2θ range.

Approximately 0.1 g of powders were filled into 10 mm diameter cavity of an off-cut zero background single crystal Si XRD holders (MTI Crystals Ltd.) to minimize background contribution. The sample holder was spun at 2 rpm to minimise texturing effect.

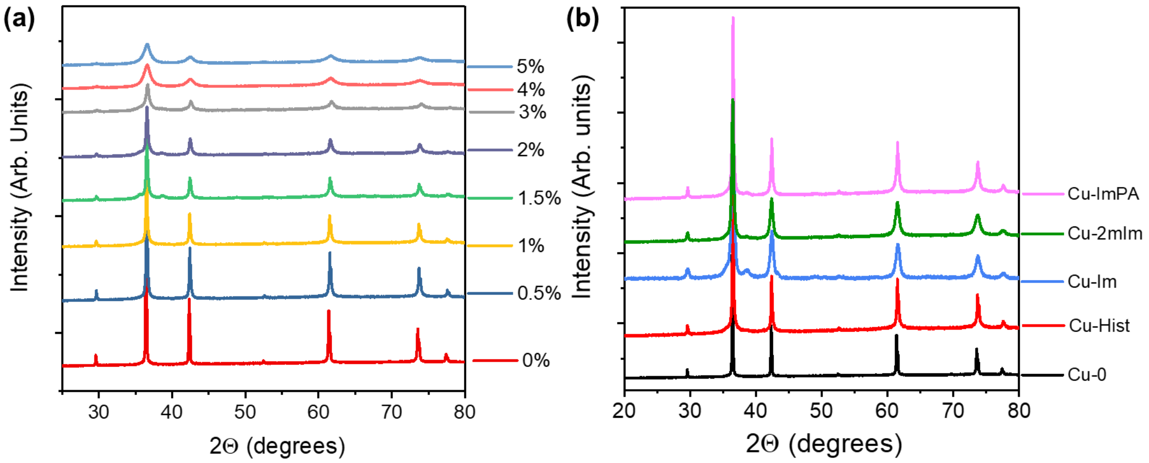

Figure S2.1: Powder X-ray diffraction spectra of samples. As-synthesized (a) Histidine-functionalized Cu_2O at different hist loading from 0 to 10 mol% and (b) Cu_2O with 2-methylimidazole, imidazolepropionic acid, imidazole and histidine surface functionalisation at 1.5 mol% loading compared to bare Cu-0.

2.2. Scanning Electron Microscopy

Scanning electron microscopy images were obtained on a JEOL JSM-IT100 with samples mounted on conductive carbon tape.

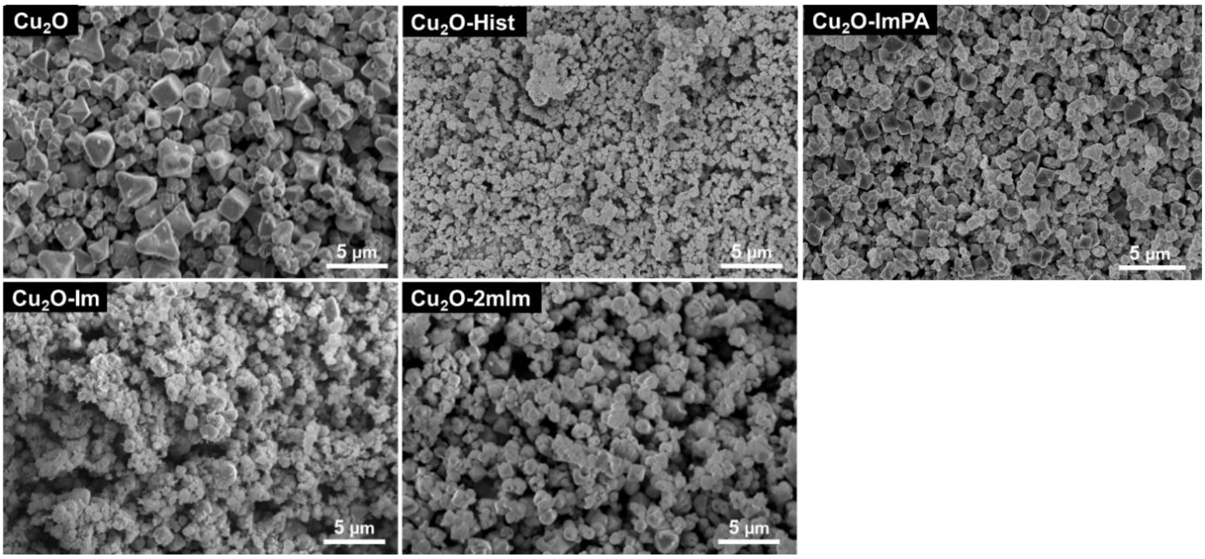

Figure S2.2: Electron micrographs of Cu_2O functionalised with various organic functional groups.

2.3. Transmission Electron Microscopy

The microstructural analysis was performed on FEI Titan 80-300 transmission electron microscope operated at 200 kV. As-grown samples can be identified as Cu_2O from the lattice spacings (**Figure S2.3**). Post-catalysis TEM indicate that the bulk of the samples have reduced to Cu (**Figure S2.4**), while some Cu_2O can be observed on the particle edges, possibly due to re-oxidation.

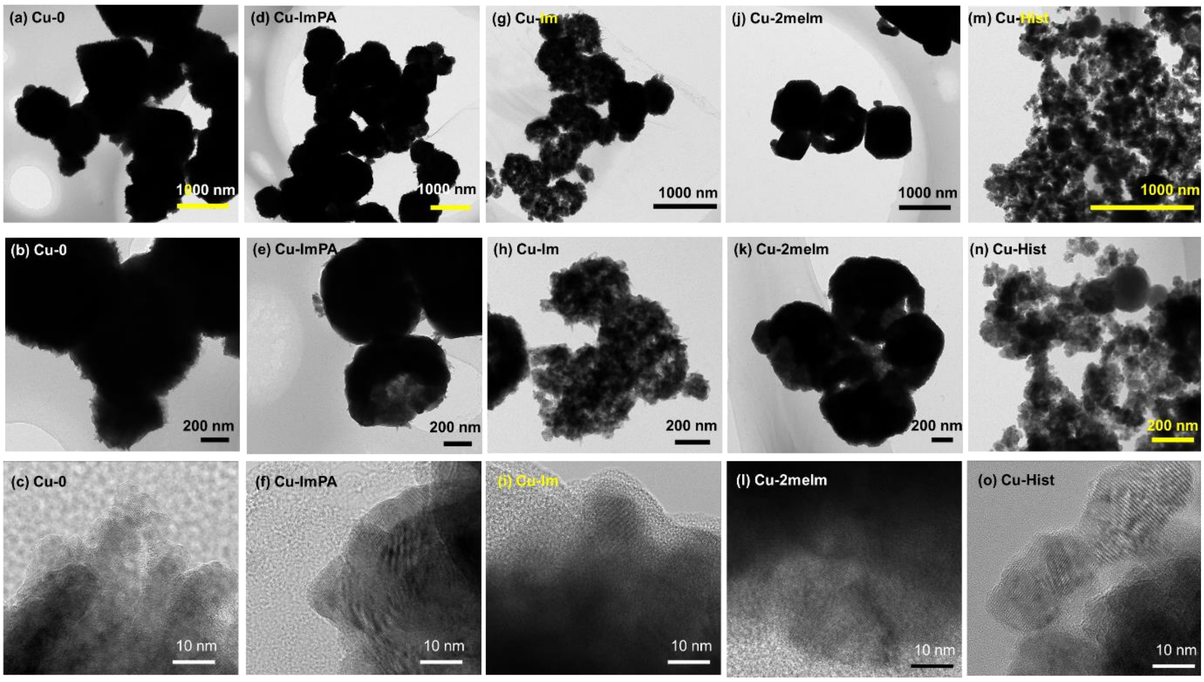

Figure S2.3: TEM of as-grown (a-c) unfunctionalized Cu_2O and Cu_2O with different functional group: (d-f) imidazole propionic acid, (g-i) imidazole, (j-l) 2-methyl imidazole (m-o) histidine.

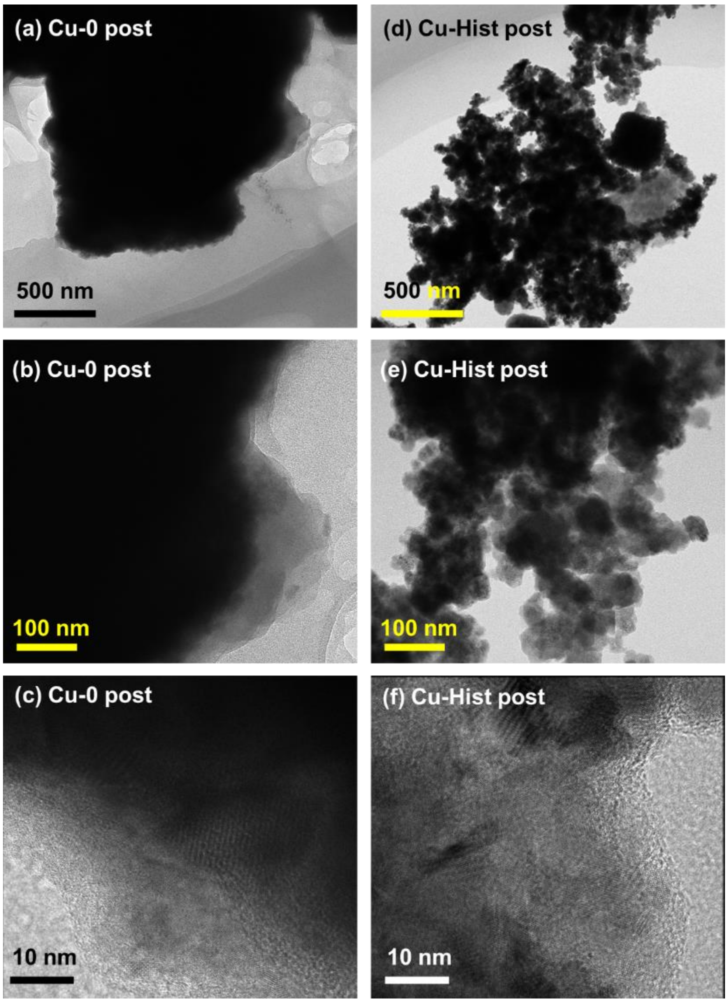

Figure S2.4: TEM post CO_2RR for (a-c) Cu-0 and (d-f) Cu-Hist.

2.4. Ex-situ Raman Spectroscopy and Fourier Transform Infrared Spectroscopy (FTIR)

Raman spectroscopy was performed with a confocal Raman microscope (LabRAM HR Evolution, Horiba Jobin Yvon) in an epi-illumination mode (top-down). A He-Ne laser (633 nm, Pacific Lasertech) was used as the excitation source. The back-scattered light was filtered through an edge filter, before being directed into a spectrograph / charge-coupled device detector (Synapse CCD). For *ex situ* Raman spectroscopy, an air objective (Olympus MPlan N, 100×, numerical aperture = 0.9) was used to collect the incident and scattered laser light. For *operando* Raman spectroscopy, a water immersion objective lens (LOMO APO water phase, 70×, numerical aperture: 1.23) protected by a 0.013 mm thin Teflon film (American Durafilm), was used. The electrochemical Raman cell was a custom-made round Teflon dish with the electrode mounted in the middle.

Ex-situ Raman spectra of pure functional group is presented in **Figure S2.5a**, while as-synthesised Cu_2O with the corresponding functional group is presented in **Figure S2.5b**.

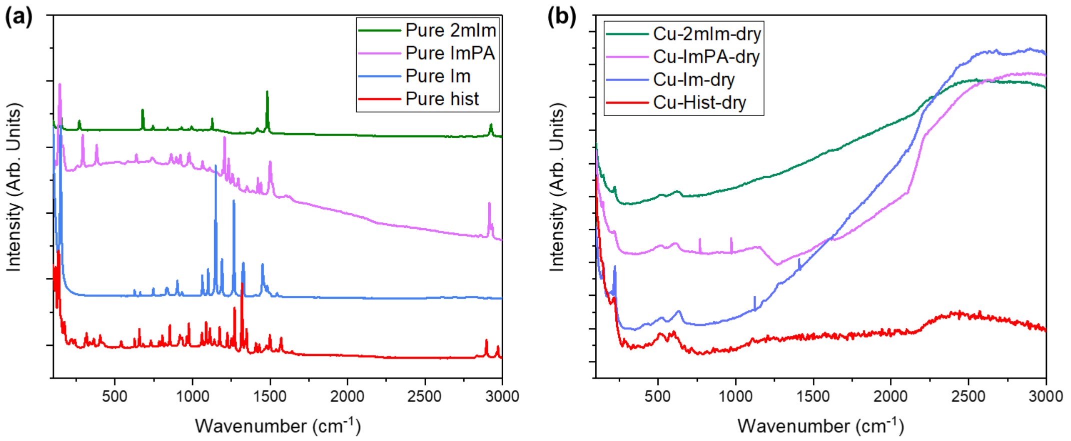

Figure S2.5: Raman spectra of (a) pure functional groups used in the synthesis, and (b) as synthesised Cu_2O with different functional groups.

FTIR was conducted using a PerkinElmer Spectrum 2000 FTIR spectrophotometer, using 16 scans and a resolution of 4 cm^{-1}. Spectra were recorded in the region 4000-400 cm^{-1}. ~1 mm sample pellets were prepared by cold pressing ~2 mg samples with potassium bromide (KBr).

In addition to the chemical binding between Cu_2O and histidine as suggested by XPS (**SI Section 2.5**), physical binding is also expected on samples synthesised with histidine (Cu-Hist) and physically mixed sample. To evaluate this, ex-situ Raman and Fourier transform infrared spectroscopy (FTIR) measurement (**Figure S2.6**) was performed. Here, we observed that Cu-Hist at a higher 10% loading show stronger and broader bands compared to 1.5% loading, indicative of a physical agglomeration of histidine forming a shell outside Cu_2O.

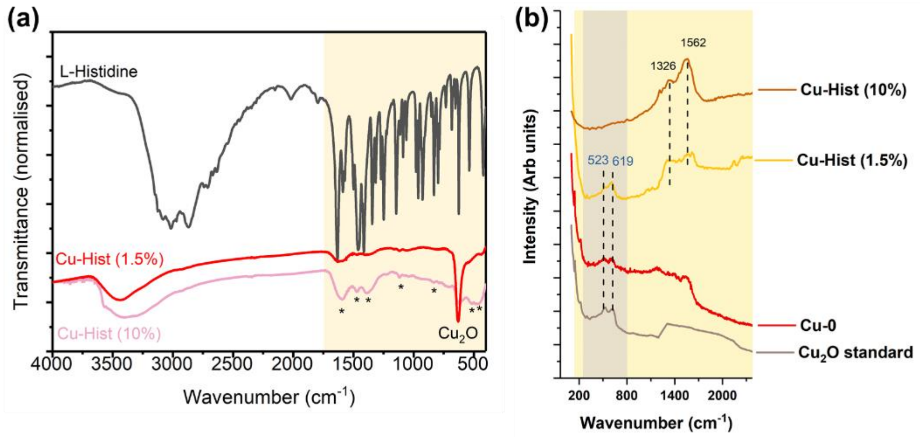

Figure S2.6: Ex-situ FTIR on pure L-histidine (black trace), Cu-Hist (1.5%, red trace), and Cu-Hist (10%, pink trace). Yellow shaded area marks the expected strongest peak position of histidine. Blue shaded area marks the expected Cu^{1+} peak (representing Cu_2O).

2.5. *Ex-situ* X-ray Photoelectron Spectroscopy

XPS data was acquired at HarwellXPS. A Kratos Axis SUPRA was used, employing monochromated Al kα (1486.69 eV) X-rays at 15 mA emission and 12 kV HT (180 W) and a spot size/analysis area of 700 × 300 µm. Source resolution for monochromatic Al Kα X-rays is ~0.3 eV. Survey spectra were obtained using a pass energy of 160 eV. High resolution spectra were obtained using a pass energy of 20 eV, step size of 0.1 eV and sweep time of 60 s. Spectra have been charge-corrected to the main line of the C 1s spectrum (adventitious carbon) set to 284.8 eV. All data was recorded at a base pressure of below 9 x 10^{-9} Torr and a room temperature of 294 K. Samples were etched using a Kratos gas cluster ion source (GCIS) minibeam ion gun with an accelerating voltage of 5 kV and a cluster size of 3000 Ar^+. GCIS etching interval was 30 s for etch step 1, followed by 300 s for etch step 2 and raster crater was 1 mm^2. Data was analysed using CasaXPS (V2.3.19PR1.0). Peaks were fit with a Shirley background prior to component analysis.

Ex-situ XPS was performed on Cu-0, Cu-Hist and pure histidine to ascertain the presence of histidine and whether it forms chemical bonds with Cu_2O. Three different depth profile based on the Ar_n^+ gas cluster ion source (GCIS) etching time were taken: surface (no etching), sub surface (30 s GCIS etching) and bulk (300 s GCIS etching).

High resolution Cu *2p* scans on Cu-0 (**Figure S2.7**) displays the expected mixture of Cu species consisting of Cu(I) and Cu(II) likely related to Cu-O bonds judging from the binding energy positions. More Cu(II) was observed on the surface of Cu-0 compared to the deeper part, corroborating the possible surface oxidation of Cu_2O over time.

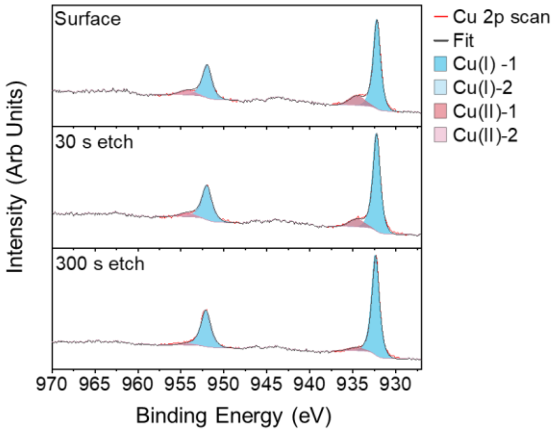

Figure S2.7: Cu2p XPS spectra of Cu-0 at different GCIS etching depth (surface/no etching, 30 s etching and 300 s etching).

We also expected a mixture of Cu species to exist on Cu-Hist. Strikingly, a third Cu species was observed at higher binding energy, matching the expected energy of Cu(II)-N bond (**Figure S2.8a**), suggesting strong chemical interaction between Cu_2O and histidine through Cu-N bond. Persistently high Cu(II)-O bond at deeper etching also suggest stronger Cu-O interaction.

Baseline N *1s* measurements on Cu-Hist and L-histidine (**Figure S2.8b**) also shows very significant difference in the N atom state. L-histidine displays clear binding energy separation for the different N atoms in the imidazole ring and the amino group (labelled "a", "b" and "c" in **Figure S2.8c**). Based on the peak area, we posit that the N *1s* peak seen on our Cu-Hist sample at 398.5 eV represents merged peak "a" and "b" on pure histidine. The merged peak is an evidence of a strong interaction of the N

with Cu species,[6] most likely with the Cu(II) at higher binding energy as corroborated from the Cu2p spectra.

while "c" nitrogen shifts to lower binding energy. The relative intensities of peak "c" on Cu-Hist is significantly smaller in all sputtering depths compared with the reference histidine, implying a decrease in the degree of protonation of the amino group.

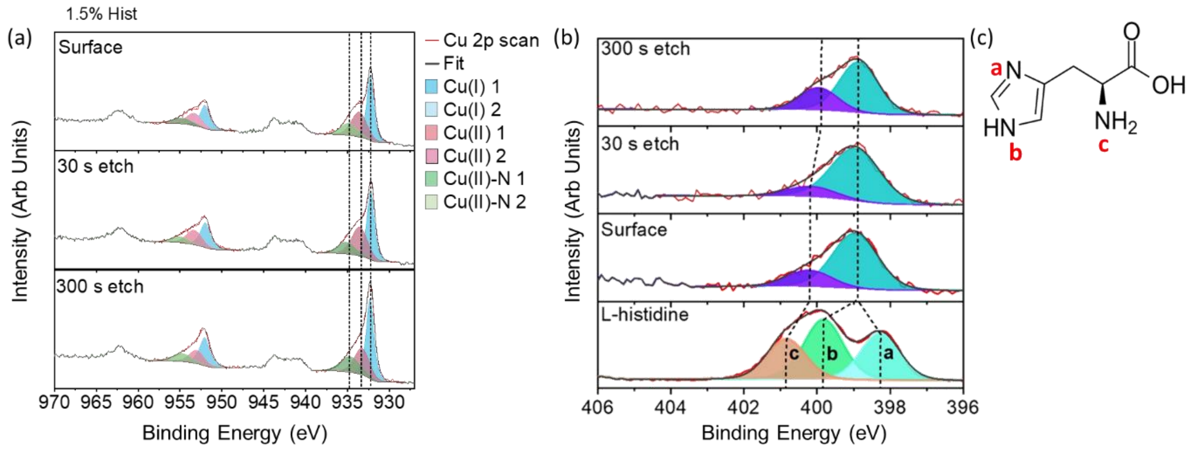

Figure S2.8: (a) Cu *2p* and (b) N *1s* XPS for Cu-Hist sample. Three depth profile scans based on gas cluster ion etching time were performed: surface (0 s), 30 s and 300 s etching. Dotted lines are guide to the eye. "a", "b" and "c" label indicates the expected N1s binding energy of pure histidine as indicated in (c).

Based on the average N at% at different GCIS etching depth of between 1.5 to 2.6 at%, and Cu/N ratio of 5.5 to 8.8 (**Figure S2.9**), we proposed that the coverage of histidine on Cu-Hist is one histidine molecule per 16.5 to 26.4 Cu atoms (assuming 3 N atoms per histidine and no significant stacking after GCIS etching)

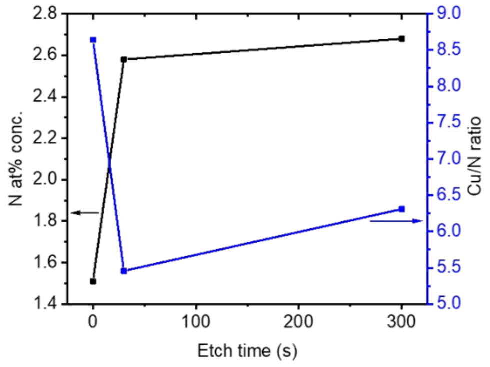

Figure S2.9: N% atomic concentration and Cu/N atomic ratio for Cu-Hist obtained from XPS measurements at different depth of 0, 30 and 300 seconds of GCIS etching.

2.6. Additional CHNS Elemental Analysis

CHNS was used to ascertain that all functionalised Cu_2O-derived samples still contain the surface functionalisation after synthesis. This is represented in the presence of clear N band (marked by broken grey lines in **Figure S2.10**).

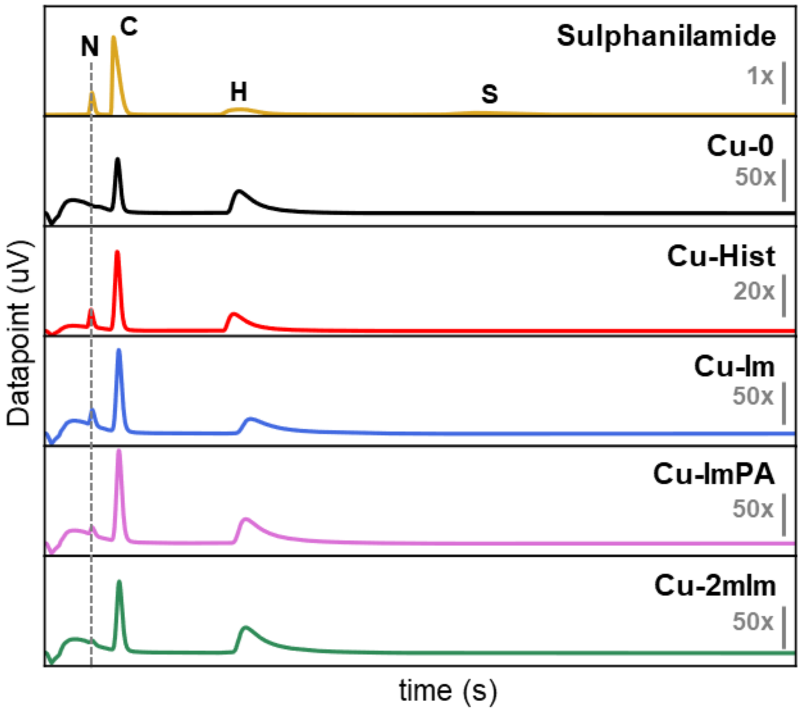

Figure S2.10: (a) CHNS data of different samples. Presence of N is only seen on functionalised samples, ascertaining their presence.

3. Electrochemical Methods

3.1. Electrode Preparation

15 mm diameter × 1 mm vitreous carbon discs polished to mirror finish with 0.3 µm alumina slurry were used as the working electrode. 5 mg of the catalyst was dispersed in 0.4 mL ethanol, 0.6 mL DI water and 40 µL Nafion solution via ultrasonication in an ice bath for 20 mins. 88 µL of the catalyst ink was deposited onto the polished glassy carbon to achieve a 0.24 mg cm^{-2} catalyst loading. The catalyst ink was then dried under a 100 W infrared lamp.

3.2. Electrochemical CO_2 Reduction Experiments

CO_2RR were performed in a custom electrochemical H-cell (**Figure S3.1**). The electrolyte compartments were separated by an anion exchange membrane and each electrolyte compartment was filled with 8 mL of electrolyte. The exposed geometric area of the working electrode is 1.13 cm^2. CO_2 (99.999% CO_2 gas from Linde Gas) was bubbled into the electrolyte using a calibrated mass flow controller (Alicat Scientific MC) at 20 sccm. The electrolyte (0.1 M $KHCO_3$) was purified using pre-electrolysis overnight before use (fixed current of -0.2 mA with carbon electrodes). 1 M $KHCO_3$ was used for CV and EIS measurement to minimise solution resistance and noise.

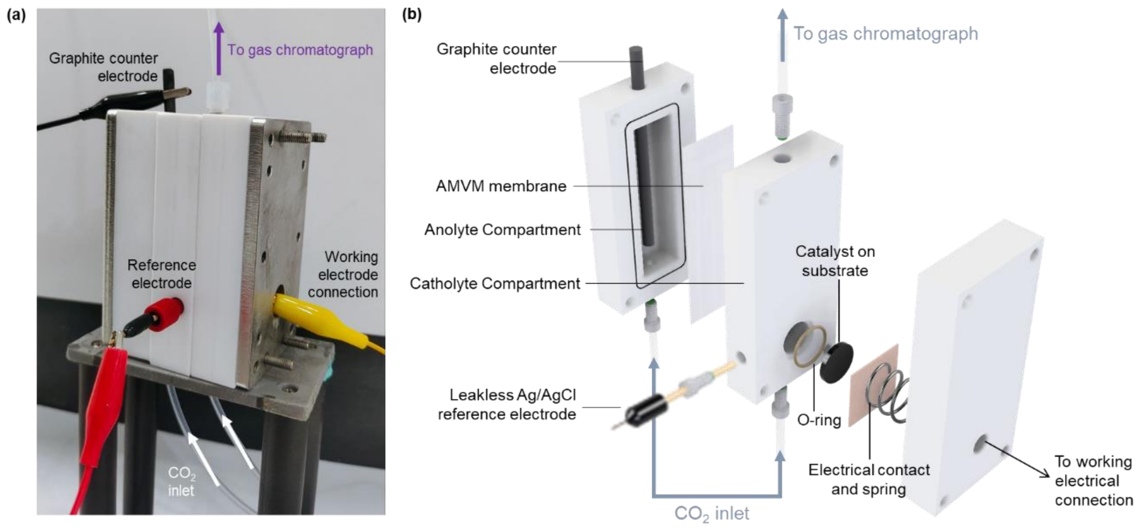

Figure S3.1: (a) Photograph and (b) schematic diagram of custom electrochemical H-Cell used in this work connected to in-line GC.

All electrochemical measurements were done using a calibrated potentiostat (Gamry 600+ and Gamry 3000). A leakless miniature Ag/AgCl reference electrode (eDAQ) was used, which was calibrated with a Hg/$HgSO_4$ electrode (Radiometer Analytical) and RHE electrode (Hydroflex, Gaskatel GmBH) in 0.5 M H_2SO_4. For CO_2RR measurements, electrolyte resistance was measured each time using high frequency electrochemical impedance spectroscopy and compensated using positive feedback correction at 85% level. No compensation for electrolyte resistance was used for cyclic/linear sweep voltammetry and pulse voltammetry measurements.

An open cell without membrane was used for pulsed voltammetry and EIS measurements. Pulsed voltammetry was conducted in 0.1 M $KHCO_3$ and the catalysts were pre-reduced at -1.125 V for 2000 s. The anodic potential was set around 0.2 to 0.3 V, where Faradaic processes were minimal. The cathodic potential is varied at 0.05 V intervals, with ΔV values ($\Delta V = V_{anodic} - V_{cathodic}$) ranging from 0.775 V to 1.875 V. Both anodic and cathodic pulses were applied for 20 s and the sampling time of the

current was 0.004 s. The anodic current decays were integrated using scipy.integrate.simps from t = 0 s to t = 5 s while mathematical fitting of the pulse from t = 0 s to t = 2 s was conducted using the scipy.optimize.curve_fit function with positive bounds.

Electrochemical impedance spectroscopy (EIS) data were collected from pre-reduced catalysts with 1 M $KHCO_3$, with higher electrolyte concentration used to minimize the R_u as well as create a more stable EIS environment. The spectrums were then collected across 30 different potentials between the range of -0.400 to -1.125 V *vs.* RHE in 0.025 V increments. The spectra were collected from 0.5 Hz to 30 kHz at 10 points per decade. The obtained Nyquist plots were individually fitted using the Simplex method on Gamry Echem Analyst (v7.9.0).

3.3. Turnover Frequency Calculation

The turnover frequency (TOF) is a measure of amount of product formed per unit time for a given amount of catalyst. Here, the TOF is calculated from the compiled averaged partial current density data according to:[7]

$$TOF = \frac{i \times N_A}{F \times n \times M}$$

Where i, N_A, F, n, and M represent averaged current, Avogadro's constant, Faraday's constant, number of electrons transferred to generate one molecule of product, and total number of participating catalyst atoms. Here we calculated both conservative estimate and the upper estimate. The latter is calculated asssuming all Cu atoms (after completely reduced from Cu_2O) is participating in the reaction. The upper estimate is calculated by assuming only histidine and one other Cu atomes next to it is participating. The result is tabulated in **Table S3.1**. Here, at the best condition of -2.0 V, Cu-Hist is able to produce C_{2+} products at between 2.90×10^{-3} and 4.19×10^{-1} s^{-1} TOF. Projected to 1 A cm^{-2} total current density achievable in high surface area gas diffusion electrode (GDE) flow setup, it is predicted to produce C2+ products between 1.88×10^{-1} and 2.72×10^{1} s^{-1} TOF.

Table S3.1: Turnover calculation from Cu-Hist data

Cu-Hist Partial Current Density Averages

V vs. RHE	iH_2 (mA)	iCH_4 (mA)	iC_2H_4 (mA)	iC_2H_5OH (mA)	iC_{2+} (mA)
-1	2.36	0.00	0.58	0.25	0.83
-1.2	2.33	0.00	2.54	1.30	3.84
-1.4	2.84	0.08	4.59	2.69	7.28
-1.6	3.64	0.21	6.46	4.54	11.00
-1.8	5.95	0.35	9.32	7.52	16.84
-2	7.76	0.30	9.85	10.01	19.86
-2.2	11.17	0.62	11.57	9.98	21.56

No of products formed (molecules)

V vs. RHE	H_2	CH_4	C_2H_4	C_2H_5OH	C_{2+}
-1	7.37E+15	0.00E+00	2.99E+14	1.31E+14	4.31E+14
-1.2	7.28E+15	0.00E+00	1.32E+15	6.76E+14	2.00E+15
-1.4	8.85E+15	6.01E+13	2.38E+15	1.40E+15	3.78E+15
-1.6	1.13E+16	1.65E+14	3.36E+15	2.36E+15	5.72E+15
-1.8	1.86E+16	2.76E+14	4.85E+15	3.91E+15	8.76E+15
-2	2.42E+16	2.38E+14	5.12E+15	5.21E+15	1.03E+16

-2.2	3.48E+16	4.84E+14	6.02E+15	5.19E+15	1.12E+16

Conservative TOF (s^{-1})

V vs. RHE	H_2	CH_4	C_2H_4	C_2H_5OH	C_{2+}
-1	2.07E-03	0.00E+00	8.40E-05	3.69E-05	1.21E-04
-1.2	2.05E-03	0.00E+00	3.71E-04	1.90E-04	5.61E-04
-1.4	2.48E-03	1.69E-05	6.70E-04	3.93E-04	1.06E-03
-1.6	3.19E-03	4.63E-05	9.43E-04	6.63E-04	1.61E-03
-1.8	5.21E-03	7.75E-05	1.36E-03	1.10E-03	2.46E-03
-2	6.80E-03	6.68E-05	1.44E-03	1.46E-03	2.90E-03
-2.2	9.79E-03	1.36E-04	1.69E-03	1.46E-03	3.15E-03

Upper bound TOF (s^{-1})

V vs. RHE	H_2	CH_4	C_2H_4	C_2H_5OH	C_{2+}
-1	2.99E-01	0.00E+00	1.21E-02	5.34E-03	1.75E-02
-1.2	2.96E-01	0.00E+00	5.36E-02	2.74E-02	8.11E-02
-1.4	3.59E-01	2.44E-03	9.68E-02	5.68E-02	1.54E-01
-1.6	4.61E-01	6.70E-03	1.36E-01	9.59E-02	2.32E-01
-1.8	7.53E-01	1.12E-02	1.97E-01	1.59E-01	3.56E-01
-2	9.83E-01	9.66E-03	2.08E-01	2.11E-01	4.19E-01
-2.2	1.41E+00	1.97E-02	2.44E-01	2.11E-01	4.55E-01

Table S3.2: Projected Turnover at 1 A cm-2 total current density

Projected Conservative TOF at 1 A cm^{-2} total current density

V vs. RHE	H_2	CH_4	C_2H_4	C_2H_5OH	C_{2+}
-1	6.75E-01	0.00E+00	2.74E-02	1.20E-02	3.94E-02
-1.2	4.01E-01	0.00E+00	7.28E-02	3.72E-02	1.10E-01
-1.4	3.42E-01	2.32E-03	9.22E-02	5.41E-02	1.46E-01
-1.6	3.25E-01	4.72E-03	9.61E-02	6.76E-02	1.64E-01
-1.8	3.75E-01	5.58E-03	9.79E-02	7.91E-02	1.77E-01
-2	4.41E-01	4.34E-03	9.33E-02	9.49E-02	1.88E-01
-2.2	5.10E-01	7.08E-03	8.80E-02	7.59E-02	1.64E-01

Projected Upper bound TOF at 1 A cm^{-2} total current density

V vs. RHE	H_2	CH_4	C_2H_4	C_2H_5OH	C_{2+}
-1	9.75E+01	0.00E+00	3.96E+00	1.74E+00	5.69E+00
-1.2	5.80E+01	0.00E+00	1.05E+01	5.38E+00	1.59E+01
-1.4	4.95E+01	3.36E-01	1.33E+01	7.83E+00	2.12E+01
-1.6	4.69E+01	6.83E-01	1.39E+01	9.77E+00	2.37E+01
-1.8	5.42E+01	8.06E-01	1.42E+01	1.14E+01	2.56E+01
-2	6.38E+01	6.27E-01	1.35E+01	1.37E+01	2.72E+01
-2.2	7.37E+01	1.02E+00	1.27E+01	1.10E+01	2.37E+01

3.4. Electrochemically Active Surface Area Measurement

Variable scan rate cyclic voltammetry was conducted for a 0.05 V potential window in the capacitive regions from 20 to 260 mV s^{-1} on reduced Cu samples. The pre-reduction was done at -1.125 V for 2000 s. The electrochemically active surface area of the catalysts is inferred from their relative surface roughness factors, which is directly proportional to the gradient from the linear fit of current density against scan rate.

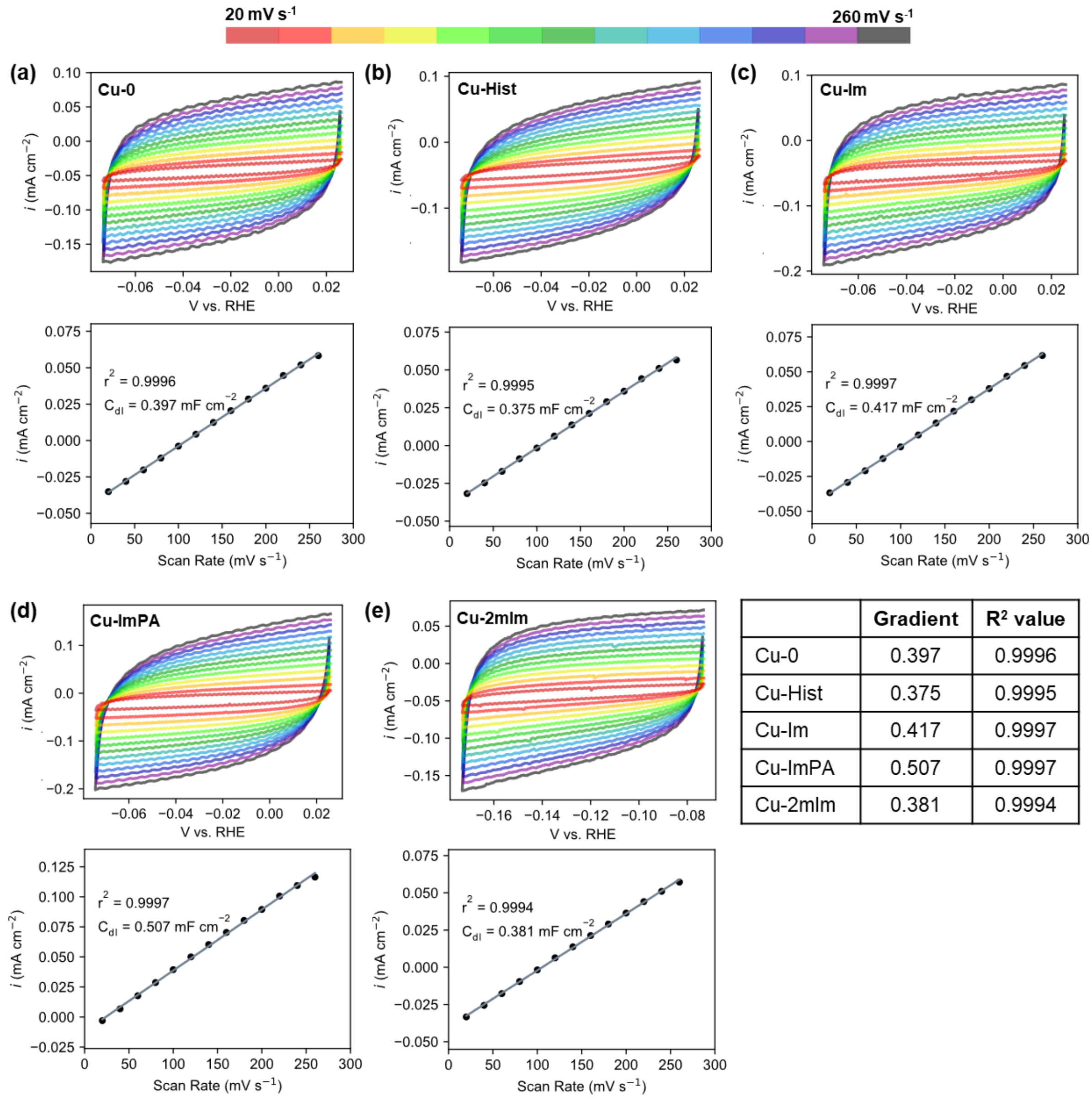

	Gradient	R^2 value
Cu-0	0.397	0.9996
Cu-Hist	0.375	0.9995
Cu-Im	0.417	0.9997
Cu-ImPA	0.507	0.9997
Cu-2mIm	0.381	0.9994

Figure S3.2: Variable scan rate cyclic voltammograms and plots of current density against scan rate for (a) Cu-0, (b) Cu-Hist, (c) Cu-Im, (d) Cu-ImPA, (e) Cu-2mIm. Double layer capacitance values were tabulated in the table (gradient unit = mF cm^{-2})

4. H-cell measurements, Product Quantification, Control Measurements, and Calibration

For gas product detection, automated valve gas chromatograph based on Agilent 7890B mainframe (Wasson ECE Instrumentation) was used. A thermal conductivity detector (N_2 carrier gas, 99.9995%, Leeden National Oxygen) was used for detecting H_2 while a flame ionization detector (He carrier gas, 99.9995%, Leeden National Oxygen) equipped with a methaniser was used for detecting C_1 to C_3 hydrocarbons and CO. Chromatograms were processed using Openlab CDS (Chemstation edition, rev. C.01.08, Agilent Technologies). Gaseous products were calibrated with 6 standard gas mixtures in CO_2 balance gas. (AG Gases Ltd., mixtures traceable to standards at the National Physical Laboratory, UK). Calibration of gas chromatograph is presented in **SI section 4.3**.

Liquid products were analysed using 500 MHz nuclear magnetic resonance (NMR, JEOL JNM-ECA 500 II) operated with DANTE selective excitation sequence[8] (2 μs pulse, 0.1 ms interval, 40 dB RF output) to suppress high concentration water signals. Each spectrum is an average of 32 consecutive scans. The samples for NMR analysis were prepared by mixing 0.5 mL of the electrolyte with 100 μL of deuterium oxide (D_2O) and 25 μL of an internal standard of 5 mM dimethyl sulfoxide and 25 mM phenol. Calibration of NMR is presented in **SI section 4.4**.

For the product quantification of CO_2RR experiments, the samples were subjected to 90 mins of chronoamperometric (constant voltage) measurement with periodic sampling of the gaseous products every 10 mins via an inline custom valve gas chromatograph and averaged. The Faradaic efficiency was calculated by comparing the number of electrons required for the amounts of detected products and recorded number of electrons measured by the potentiostat. All CO_2RR Faradaic efficiencies and current densities are averaged over three independent measurements and the error is taken to be the standard deviation of the three values obtained.

Error representation for summed products is calculated based on the following formula:

$$\Delta z = \sqrt{\Delta x^2 + \Delta y^2 + \cdots}$$

$$\Delta\ \mathrm{Others} = \sqrt{(\Delta\ \mathrm{Formate})^2 + (\Delta\ Carbon\ Monoxide)^2 + (\Delta\ Acetate)^2 + \cdots}$$

4.1. Optimization of Functionalisation Loading

The optimum functionalisation loading was determined by synthesising seven samples with 0 to 5% Hist loading. We observe critical loading amount of 1%, below which the CO_2RR product distribution still resembles unfunctionalised Cu-0 (**Figure S4.1**). Higher Hist loading tend to increase HER. Cu-Hist with 1.5% loading was selected as the optimum based on the amount of C_{2+} products.

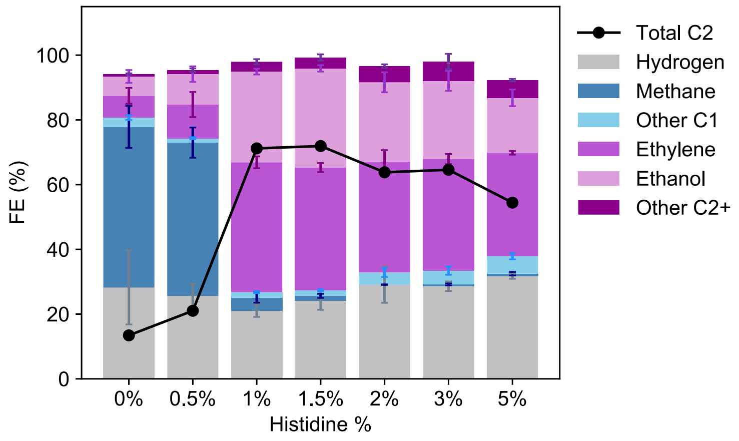

Figure S4.1: Comparison of CO_2RR products for Cu_2O synthesized with varying molar ratios of histidine at -1.8 V *vs.* RHE.

4.2. Baseline Experiments Under Argon Flow

We conducted control experiments for Cu-Hist catalyst by swapping CO_2 to Ar flow in 0.1 $KHCO_3$ electrolyte to exclude the possibility that the histidine functionalities may contribute as a carbon source for CO_2RR (**Figure S4.2**). Average FE of 92.84% for H_2 were observed for a total FE of 93.27%, with the remaining 0.429% arising from impurities in the argon gas. No peaks corresponding to CO_2RR products were observed for either the NMR spectra or the FID detector of the gas chromatograph, confirming that the hydrocarbon products products reported in this study are the sole result of CO_2RR catalysts.

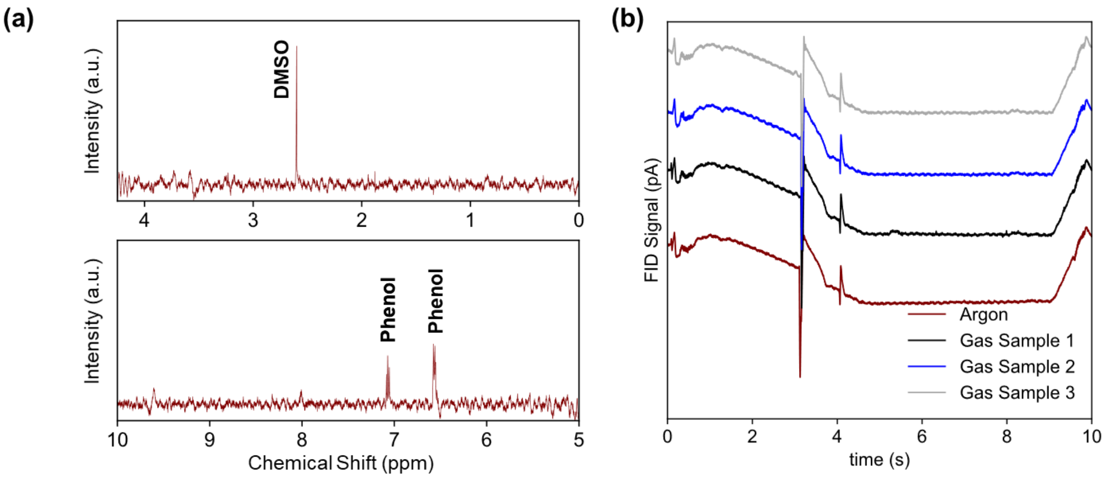

Figure S4.2: Baseline measurement with Ar-purged H-Cell showing (a) Post-catalysis NMR spectra of electrolyte and (b) gas chromatograph from in-line sampling of gaseous products.

4.3. Calibration of Gas Chromatograph

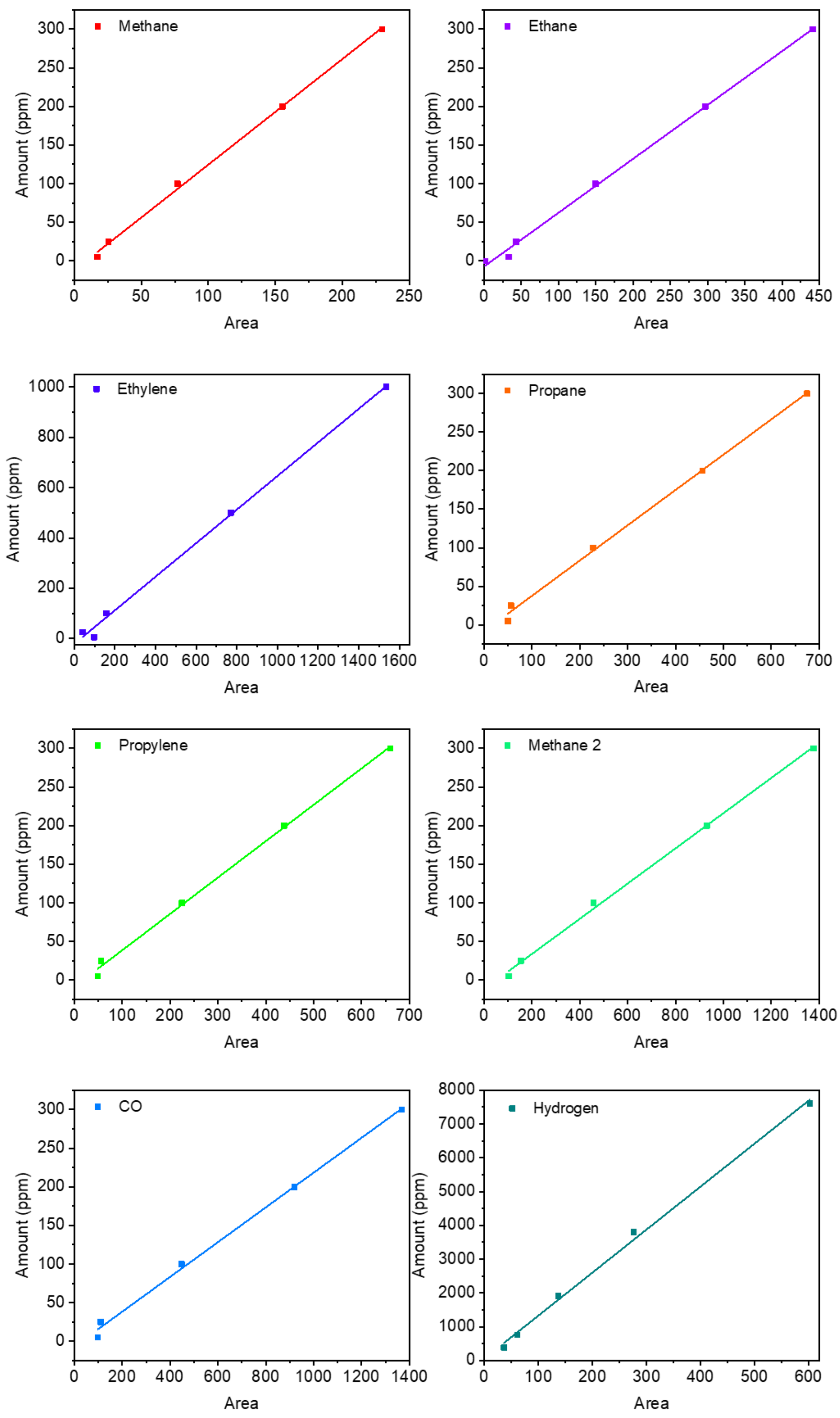

Figure S4.3: Calibration curve for gas products quantification using gas chromatograph.

4.4. Calibration of NMR

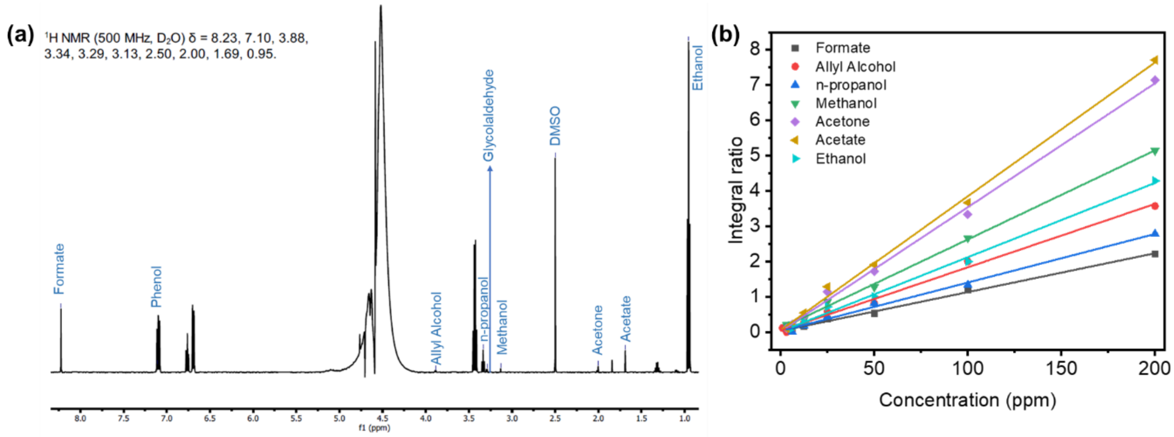

Figure S4.4: (a) Representative for NMR calibration profile and (b) calibration curve for NMR for liquid product quantification.

4.5. Partial Current Densities of H_2 and C_{2+} Products

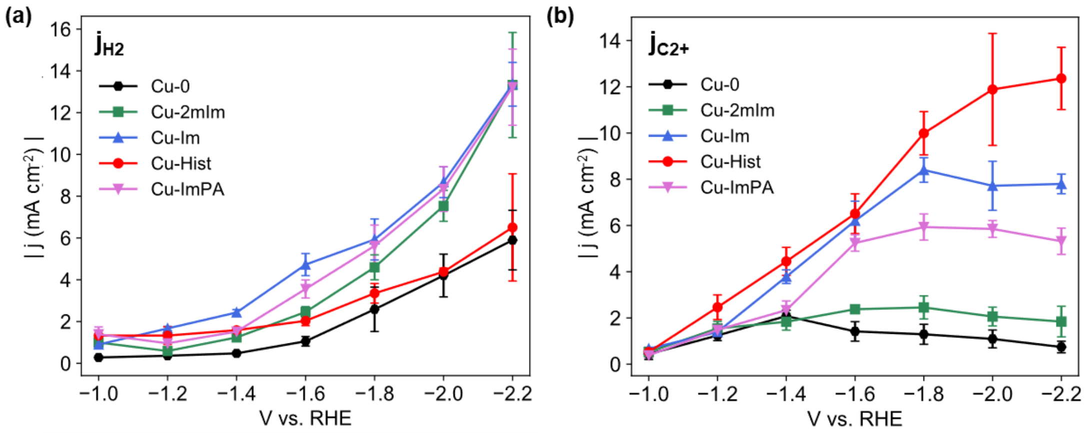

Figure S4.5: Partial current density of (a) H_2 and (b) C_{2+} products of Cu_2O-derived Cu with and without surface functionalisation group. Error bars represent standard deviation from 3 independent measurements.

4.6. Additional H-Cell Control Experiments with Glassy Carbon Substrate and Cu_2O with Physically Mixed 2% Histidine

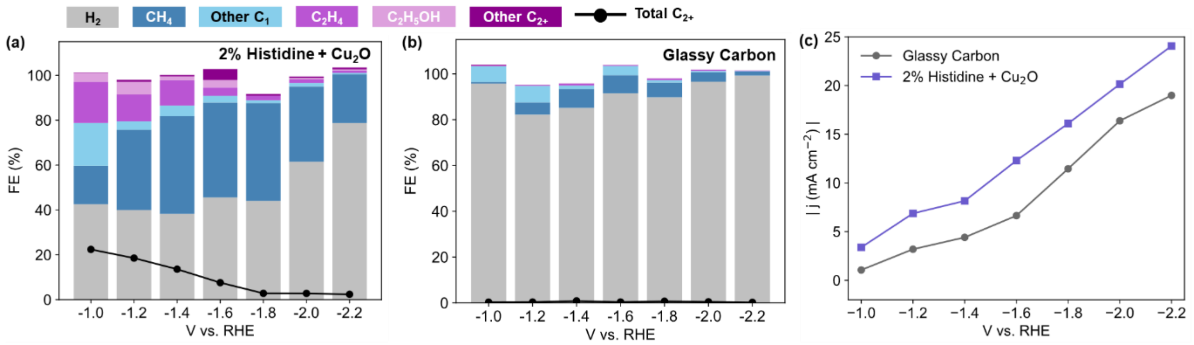

Figure S4.6: Faradaic efficiencies of control samples of (a) Cu-0 physically mixed with 2% histidine, (b) blank glassy carbon, and (c) the corresponding current density.

4.7. Comparison of Catalytic Performance at -1.6 V between Cu_2O with Additional Functional Groups

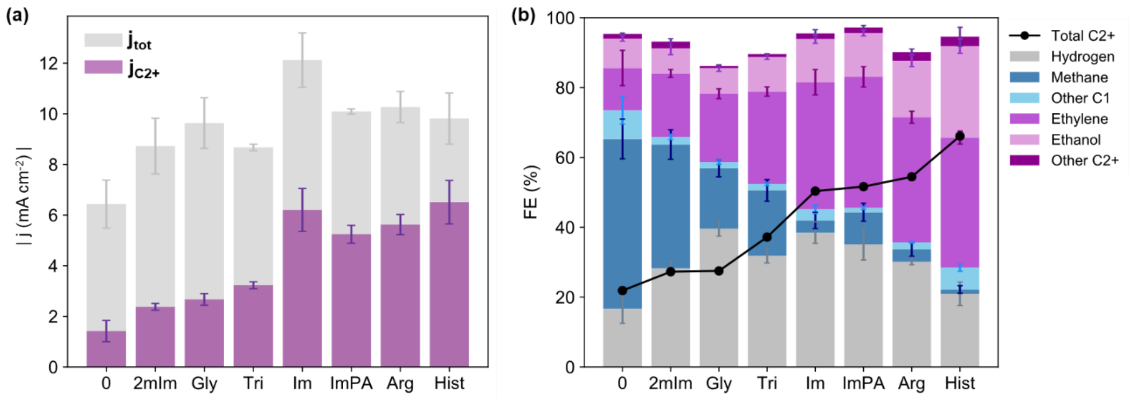

Figure S4.7: FE and j_{C2+} comparison of bare Cu-0 and functionalised Cu-2mIm, Cu-Im, Cu-ImPA, and Cu-Hist. Three additional functionalisation (Cu-Gly, Cu-Tri and Cu-Arg) was added to help validate the use of R_1, R_2, CPE_1, CPE_2, Q_{an}, k_{2nd}, and C_{2nd} as activity descriptor in functionalised Cu_2O-derived Cu. Error bars represent standard deviation from 3 independent measurements.

5. In-situ Raman Spectroscopy

During in-situ Raman measurement, the custom-made PTFE open cell is filled with CO_2 (or N_2) purged 0.1 M $KHCO_3$ was used to hold 10 mm electrode and perform the operando electrochemistry **(Figure S5.1**). Gas purging is maintained throughout the in-situ measurement.

An additional Cu_2O electrode was synthesised using electrodeposition on Cu disc as reported previously.[9] This sample is used as a baseline comparison to the solution grown Cu-0. Briefly, the Cu_2O film was galvanostatically deposited onto the copper substrate from a mixture of 0.3 M $CuSO_4{\cdot}5H_2O$ (98.5−101%, GCE), 3.2 M NaOH (99−100%, Chemicon) and 2.3 M lactic acid (85%, Sigma−Aldrich) at -1.4 mA cm^{-2}, 60 °C for 150 s.

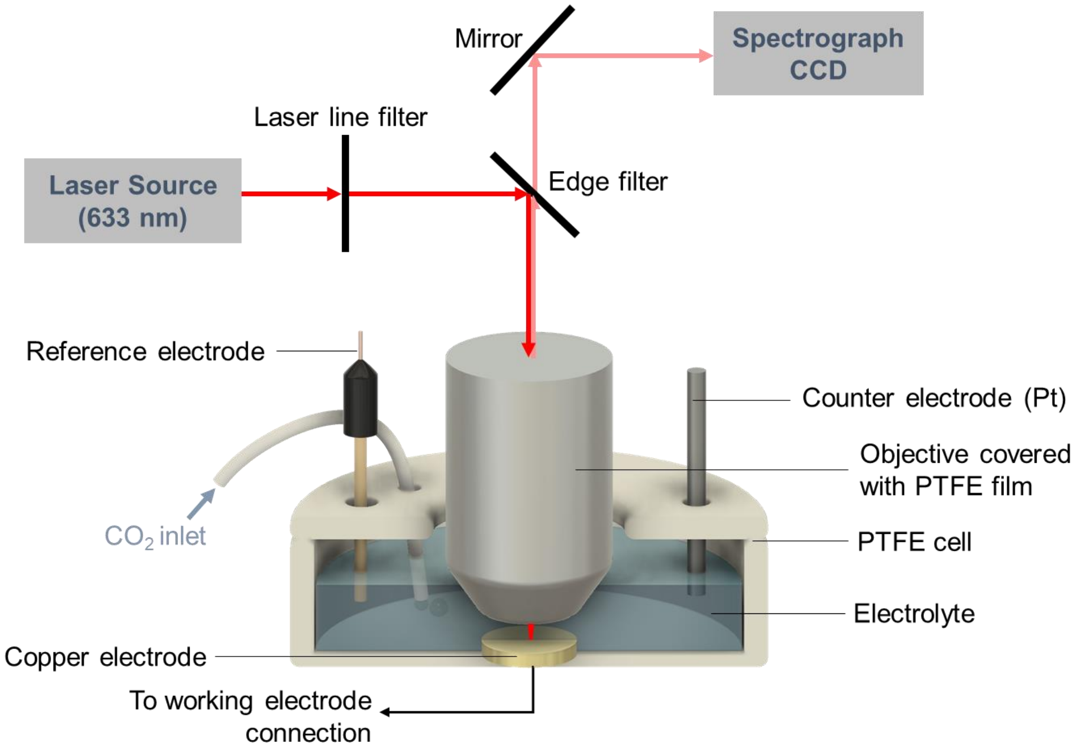

Figure S5.1: Schematics of the in-situ Raman cell setup

A Gamry 600 potentiostat was used to control the potential during operando Raman spectroscopy measurements. The chronoamperometric current profiles at selected applied voltages are presented in **Figure S5.2**.

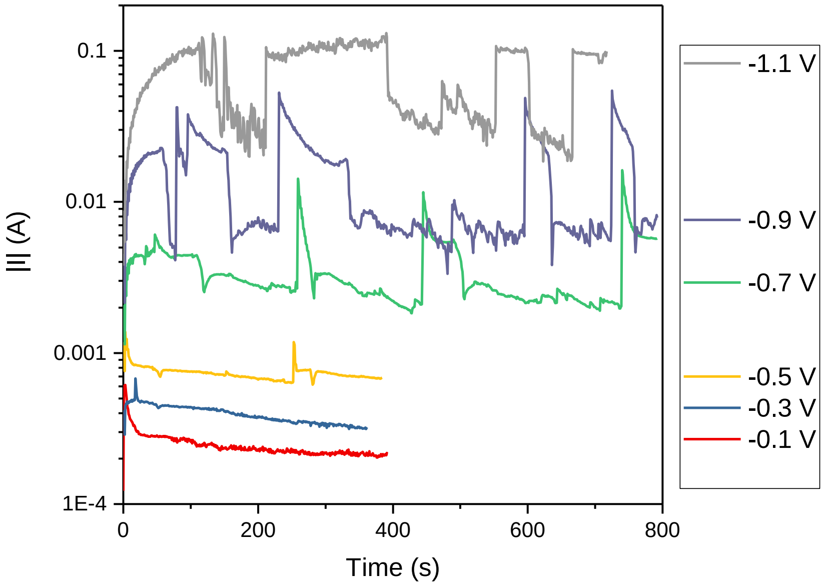

Figure S5.2: Chronamperometry current profile during in-situ Raman at respective cathodic potentials. The fluctuation is due to microscope being moved up and down. Each potential was kept for about 400 to 800 s to allow sufficient time to collect Raman spectra.

5.1. Supplementary In-situ Raman Measurements

In addition to the conditions described in the main text, we also conducted baseline operando Raman measurements on blank Cu disc, freshly electrodeposited Cu_2O and Cu-Hist at different conditions (**Figure S5.3**) to identify Raman signals of histidine and expected Cu-*CO interactions on Cu_2O-derived Cu.

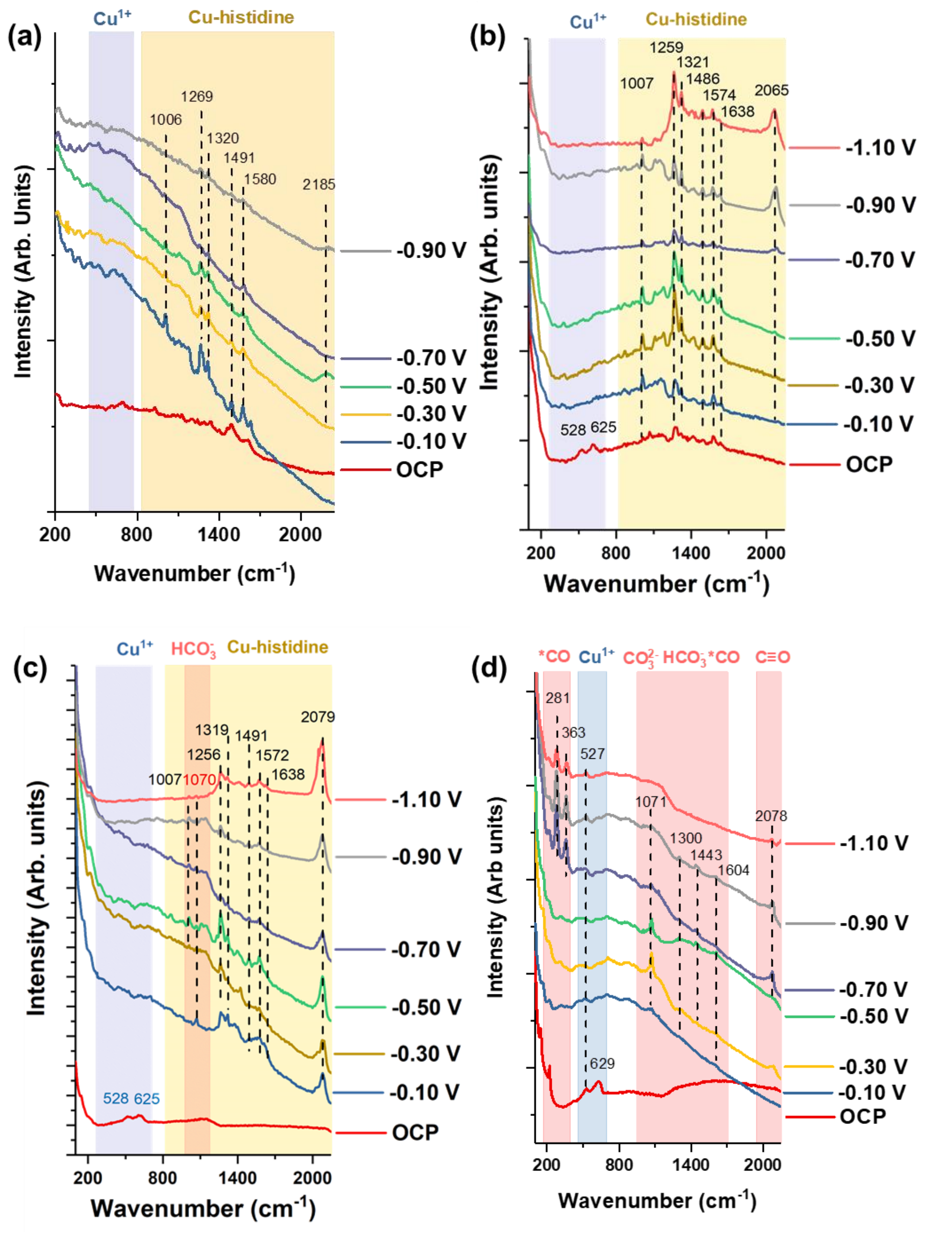

Figure S5.3: In-situ Raman in N_2 Purged 0.1 M $KHCO_3$ with dissolved 0.025 M Histidine on (a) polished bare Cu disc and (b) freshly electrodeposited Cu_2O on Cu disc. (c) In-situ Raman on Cu-Hist sample in N_2 purged 0.1 M $KHCO_3$ electrolyte. (d) In-situ Raman on freshly electrodeposited Cu_2O on Cu disc in CO_2 purged 0.1 M $KHCO_3$ electrolyte

5.2. Explanation on the attraction of histidine towards cathode upon electrochemical bias

Histidine is likely to be partially deprotonated on the carboxylic group at the electrolyte pH used in the in-situ Raman studies. At pH ≈6.8, histidine is expected to exist as a mixture of His+ and His± form,[10] and this form still allows some attraction to the cathode from electrolyte bulk. Histidine is also known to interact with CO_2, forming zwitterion carbamate even at alkaline condition,[11] allowing the more positively charged imidazole ring to be attracted to the cathode surface.

Moreover, the attraction of histidine from electrolyte bulk to the double layer can also be promoted by the cations accumulation upon application of negative bias. The concentration of alkali cations is known to build up upon cathodic bias application, and are stabilised by repulsive charge among the positively charged cations and the solvent.[12] Such layer would ameliorate the repulsion effect and help the transport of histidine across the double layer.

We note that histidine adsorption on Cu has been reported earlier, also under cathodic bias.[13] At increasingly higher pH, histidine adsorption can still be observed under Raman under electrochemical cathodic bias, albeit with some change on how the histidine binds to the surface.

5.3. Explanation on histidine-Cu interaction during CO_2RR

The main sample (marked as Cu-Hist) is Cu_2O synthesised in presence of 1.5 mol% histidine in the precursor solution. We expect two kinds of interactions between histidine and Cu_2O precursor, physical and chemical. We expect the histidine to encapsulate the Cu_2O crystals physically. On the surface, our data shows histidine is bound to Cu_2O surface chemically.

The physical interaction between Cu_2O and histidine can be seen in the ex-situ FTIR spectra (**Figure S2.6**). Weak bands that can be attributed to L-histidine was clearly observed on Cu-Hist at 1.5% loading. Stronger and clearer bands were observed on 10%, indicating thicker encapsulation and stronger physical interaction.

The chemical interaction between Cu_2O and histidine in Cu-Hist can be seen in the ex-situ XPS (**Figure S2.8**). Here, clear additional Cu *2p* peaks that can be attributed to Cu-N bonds, and merged a and b N1s peaks indicating strong interaction between Cu and both proxima- and tele-N in the imidazole ring.

From the evidence we gather in in-situ Raman experiments (Main **Figure 3** and **Figure S5.3**), we strongly believe that histidine is retained on reduced Cu_2O-derived Cu surface after reduction. Such retention is possible due to the following proposed explanations:

(1) Proximity effect due to physical encapsulation. We have established earlier that Cu_2O precursor is surrounded by histidine. In this regard, histidine does not need to be attracted from the electrolyte bulk during the reduction process.

(2) Histidine is also known to form complex with Cu.[14] We posit that such Cu-histidine complex can be easily formed during Cu_2O reduction, which could subsequently latch on to the reduced surface and form a new active surface (as Cu-histidine complex).

(3) Electrochemical attraction and interaction with CO_2/HCO_3^- system. We see new bands forming that are not previously seen on dry histidine/Cu-histidine without applied potential.

To support this argument, we performed additional control experiments in **Figure S5.4**, comparing dry histidine powder, wet histidine on Cu surface, drop casted Cu-hist on open circuit, and with -0.1 V vs

RHE under N_2 and CO_2 purging. Here, we see clear shift and/or broadening of the peaks when compared to dry histidine, suggesting new interaction when in contact with Cu upon cathodic bias application. The most interesting observation is the appearance of new Raman band near 1625 and 2080 cm^{-1} upon application of electrochemical bias. Bands near this position usually attributed to *v*-C=O and *v*-C≡O modes on metal surfaces.[15] However, the fact that this band is also present in N_2 purged experiment, we posit that it is a clear indication of Cu-histidine interaction upon application of cathodic bias.

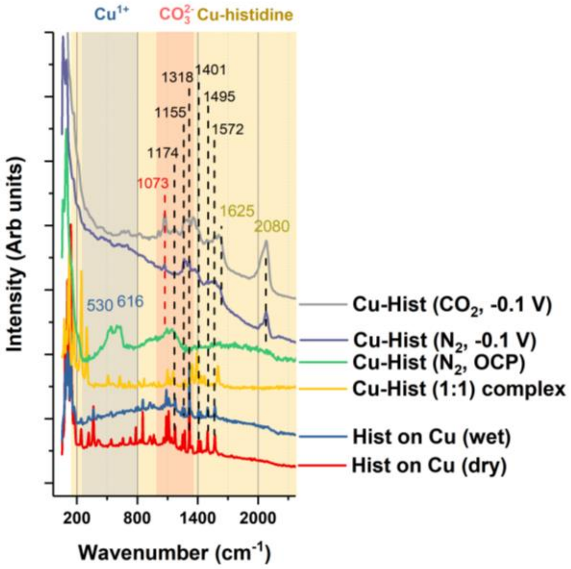

Figure S5.4: Comparison between dry histidine, wet histidine (with 0.1 M $KHCO_3$), Cu-Hist at OCP, and Cu-Hist at -0.1 V with N_2 and CO_2 purging. Band broadening was observed between wet/dry histidine and Cu-Hist sample. More importantly, there are some bands that are present only with applied cathodic voltage (1626 and 2080 cm^{-1}).

As further proof that the histidine can be retained on the surface with voltage application, we performed flushing experiment where we flush electrolyte with fresh $KHCO_3$ whilst the applied voltage is still turned on at -0.7 V (**Figure S5.5**). The result is that, albeit with reduced intensity, we can still observe bands belonging to histidine-Cu interaction at cathodic voltage.

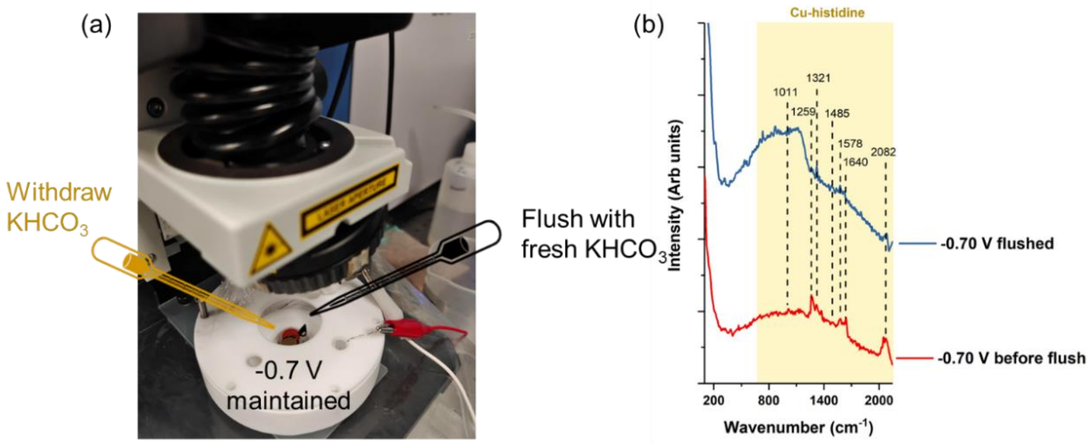

Figure S5.5: (a) Electrolyte purging experiment: Raman measured before and after flushing with 50 mL fresh $KHCO_3$ under continuous CO_2 stream and -0.7 V applied. Raman cell volume is approx. 30 mL. (b) Raman spectra before and after purging, showing persistent histidine bands after flushing.

6. Density Functional Theory Calculations

6.1. Details of the computational methods

All presented DFT calculations are spin-polarised. A (4×4) Cu(100) surface slab built from the optimised bulk structure (lattice parameter a = 3.634 Å) was used for the calculations with and without adsorbed histidine. This surface slab is sufficiently large to produce a surface concentration of histidine in agreement with the Cu/N ratio obtained during XPS experiments (**SI Section 2.5**). A vacuum layer of 16 Å separated periodic images in the z direction. A dipole correction was applied perpendicular to the surface. The energy of small gas-phase molecules (e.g., CO, H_2, CH_4, C_2H_4) was calculated by placing the molecule in a box of size 15×15×15 $Å^3$, while gas-phase histidine was placed in a box with dimensions 20×20×20 $Å^3$.

We computed Gibbs free energies to compare the stability of the various intermediate states. The Gibbs free energies (G) were calculated with the following equation (1):

$$G = E_{elec} + E_{ZPE} + \int_0^T C_P dT - ST + E_{solv} \qquad (1)$$

Where the contributions to the free energy are respectively the total energy of the system at 0 K, the zero-point energy, the enthalpic temperature correction, the entropy correction, and the solvation energy of the adsorbate. The term E_{ZPE} is calculated from the harmonic vibrational frequencies as $\sum \frac{1}{2} h\nu_i$, where h is Plank's constant and ν_i is vibrational frequency i. The term with the integral is the energy extrapolation from 0 K to the working temperature, T (298 K for our purposes). The entropy (S) is calculated using the harmonic vibrational energies, assuming no real translational and rotational degrees of freedom for adsorbates. We assume that any change in the vibrational frequencies of the solid is insignificant. The solvation correction (E_{solv}) was determined using the simple, although widely implemented, approach from Peterson et al.[16] A discussion on the selection of the solvation correction scheme is provided in **SI Section 6.6.**

We model the coupled proton-electron transfer (CPET) reactions using the computational hydrogen electrode (CHE) of Norskov.[17] In this approach, the voltage of reference (zero) is defined as for the reversible hydrogen electrode (RHE), where gas-phase hydrogen is converted into protons and electrons (reversibly) at zero volts ($H^+ + e^- \rightarrow ½ H_2$). Because the previous reaction is in equilibrium, one can make the approximation that the chemical potential of the proton-electron pair, $\mu(H^+) + \mu(e^-)$, equals half of the chemical potential of gas-phase H_2, $\frac{1}{2}\mu(H_2)$. This last quantity can be trivially approximated via DFT calculations. Finally, the chemical potential of the proton-electron pair can be adjusted based on the applied potential (U) with the equation (2):

$$\mu(H^+) + \mu(e^-) \rightarrow \frac{1}{2}\mu(H_2) - eU \qquad (2)$$

where e is the elementary positive charge. It is assumed that both proton and electron transfer occur in concert during an electrochemical step. For all of the electrochemical steps, we took the free energy change between intermediates to indicate the feasibility of the electrochemical process, *i.e.*, no energy barriers were calculated. As previously estimated,[16] we expect that the proton transfer from the solution to the adsorbates has low barriers, especially under negative potential.

To determine the free energies of intermediates involved in forming a C_2 product, we simplified the calculations by assuming that one CO molecules adsorb first at the bare Cu(100) surface. Therefore, the total energy of the reference configuration **0** (**Figure 4** of main text) is the sum of the energies of the histidine-Cu/Cu(100) slab (**Figure S6.2b**), a gas-phase CO_2 molecule, and an adsorbed *CO ($E_{CO/Cu(100)}$-

$E_{Cu(100)}$).on bare Cu(100). The *CO specie is assumed to be mobile on the surface, *i.e.* entailing low energy diffusion barriers. After configuration **4** is formed (**Hist**–CO complex, **Figure 4b** of main text), the *CO adsorbed on bare Cu(100) approaches the **Hist**–CO complex leading to configuration **5**. At this point, the energy of bare Cu(100) (without adsorbates) substitutes the energy of *CO on bare Cu(100) in the calculation of the total energy of the system.

6.2. Surface model

Histidine adsorbate. There are two tautomeric forms of neutral histidine in the gas phase. The main difference between the two is the position of the hydrogen atom in the heteroaromatic nitrogen atoms in the imidazole ring, N^{δ} and N^{ε}. These histidine forms are commonly referred to as δ and ε tautomers. According to DFT calculations of histidine conformers in the gas phase, the proportions of ε histidine tautomers are higher than the δ tautomer.[18] However, protonated and deprotonated versions of histidine may be favoured when solvated at different pH. At the relevant pH of this study, the histidine molecule is expected to be in the δ form[18] and deprotonated at the carboxylic group,[14] leaving a carboxylate group (R-COO$^-$). We considered then that solvated histidine would exist in the deprotonated form with the N^{δ} hydrogenated. Moreover, the histidine molecule can spontaneously form complexes with Cu(II) ions.[19] Our tests showed that the Cu atom prefers to bind the dehydrogenated N atom from the imidazole ring (**Figure S6.1**). It was determined in a systematic study exploring the stability of Cu(I) and Cu(II) complexes with biological ligands containing N, O, and S that both cations prefer to bind N over the other atoms.[20]

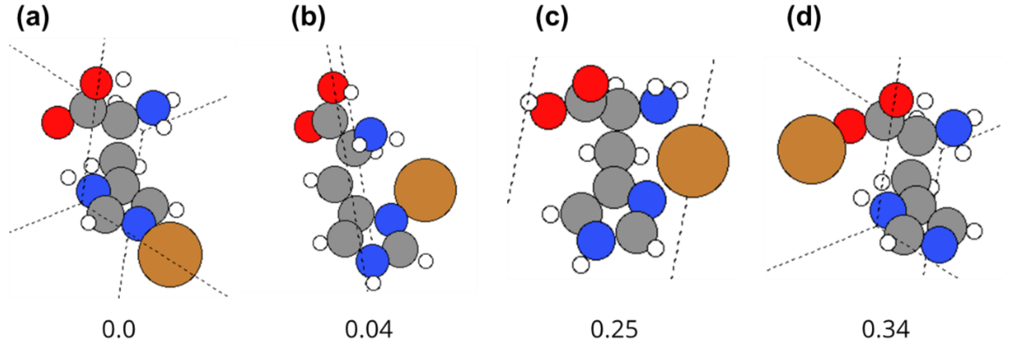

Figure S6.1: Various configurations for the interaction of histidine with a Cu atom. The relative energies for each configuration are shown underneath each configurations in eV.

After investigating the histidine tautomers and the possibility of histidine-Cu complex formation above, we arrived at the Cu-histidine surface model presented in **Figure S6.2a**. The adsorption of histidine with an additional Cu adatom (green) reflects the spontaneous formation of the histidine-Cu complex during the severe surface reconstruction of Cu_2O at CO_2RR potentials that is likely to involve Cu dissolution and reprecipitation.[21] A further modification of the adsorbed histidine-Cu system was implemented to account for the experimentally observed high-resolution N1s peak analyses shift (**SI section 2.5**), interpreted as the amino group of histidine being deprotonated. **Figure S6.2b** shows the surface model used as a reference structure for the calculations discussed in the main text. We refer to this reference structure as **histidine-Cu/Cu(100)**.

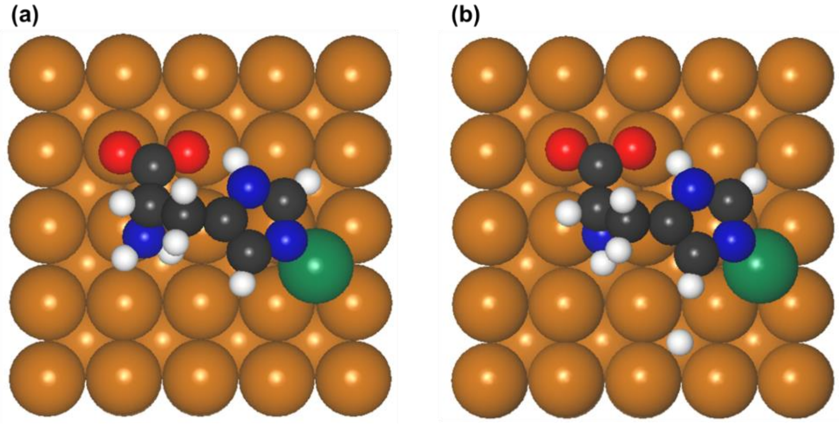

Figure S6.2: (a) Deprotonated histidine-Cu complex adsorbed on a Cu(100) 4×4 supercell slab. The deprotonated form of histidine was obtained by removing the proton from the carboxylic group, leaving a carboxylate group (R-COO^-). (b) One hydrogen from the histidine's amino group detaches and binds the Cu surface. This last modification simulates the deprotonation of the amino group as observed in our experiments via high-resolution N1s peak analyses (see main text). The green atom denotes the Cu adatom from the histidine-Cu complex representing both the spontaneous formation of the complex and the expected roughness of the Cu surface after the reduction of the Cu_2O electrode.

6.3. Correction to CO gas-phase molecule

We explored if the PBE functional can appropriately describe the thermodynamics of the reactions of interest. For these purposes, we calculated the gas-phase molecules involved in the reactions shown in **Table S6.1**. The reaction enthalpies forming CH_4, HCOOH, CH_3OH, C_2H_5OH, and C_2H_4, were calculated from both CO_2 and CO as reactants. We obtained a mean absolute error (MAE) of 0.26 eV while comparing our DFT calculations with the standard enthalpy of reaction reported experimentally (estimated at 25 °C and 1 atm). However, it is evident that only the reactions in which CO is a participant have the most significant errors (|Error| >0.30 eV), while the energies of CO_2 and the main products are described adequately. Therefore, a simple correction can be added to the energy of gas-phase CO to appropriately describe the thermodynamics of the reactions involved during the CO_2/CO electroreduction. A correction of -0.44 eV minimises the MAE to a value of 0.05 eV. This procedure was applied in a previous work using the RPBE functional, where the energy of the CO_2 and HCOOH molecules required a correction instead of CO.[16] In their work, they compared the results obtained from both RPBE and PBE functionals arriving at the same conclusions.

Table S6.1: Experimental reaction enthalpies (ΔH) of representative reactions of the CO_2/CO electroreduction. The calculated reaction energies using the PBE functional (ΔE) are also shown with the error compared with the experimental values.

#	Reaction	ΔH°(Exp)	ΔE(PBE)	Error
1	$CO_2 + H_2 \rightarrow CO + H_2O$	0.43	0.87	0.45
2	$CO_2 + 4H_2 \rightarrow CH_4 + 2H_2O$	-1.71	-1.64	0.07
3	$CO + 3H_2 \rightarrow CH_4 + H_2O$	-2.13	-2.51	-0.37
4	$CO_2 + H_2 \rightarrow HCOOH$	0.15	0.07	-0.09
5	$CO + H_2O \rightarrow HCOOH$	-0.27	-0.80	-0.53
6	$CO_2 + 3H_2 \rightarrow CH_3OH + H_2O$	-0.55	-0.53	0.02
7	$CO + 2H_2 \rightarrow CH_3OH$	-0.98	-1.41	-0.43
8	$CO_2 + 3H_2 \rightarrow 1/2C_2H_5OH + 3/2H_2O$	-0.89	-0.89	0.00
9	$CO + 2H_2 \rightarrow 1/2C_2H_5OH + 1/2H_2O$	-1.32	-1.77	-0.45
10	$CO_2 + 3H_2 \rightarrow 1/2C_2H_4 + 2H_2O$	-0.66	-0.56	0.10
11	$CO + 2H_2 \rightarrow 1/2C_2H_4 + H_2O$	-1.09	-1.43	-0.34

6.4. Effect of applied bias on CO_2RR

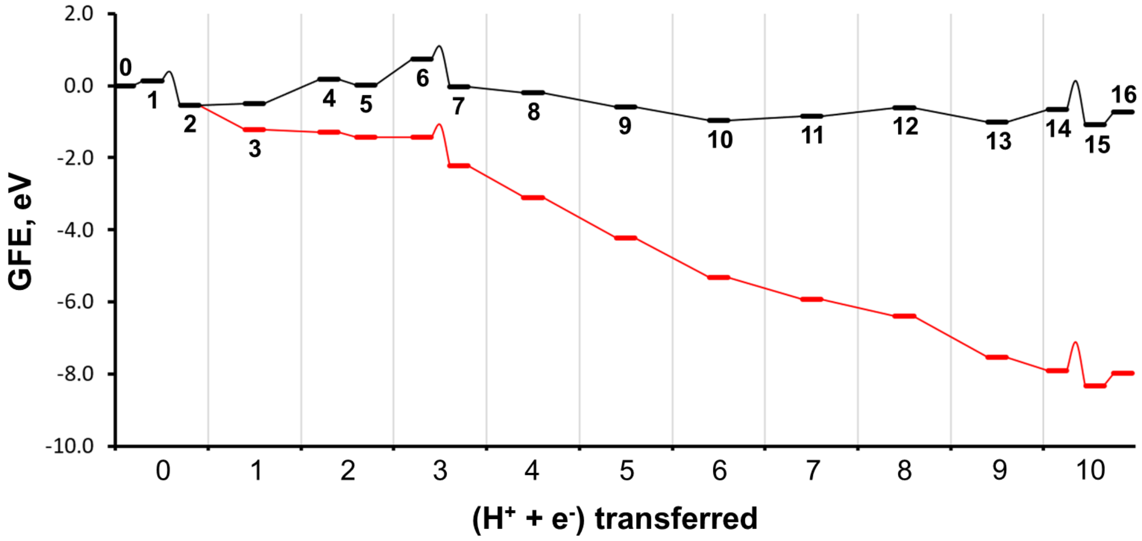

Figure S6.3: Gibbs free energy diagram for the CO_2RR to C_2H_4 through the histidine-assisted mechanism under no applied bias (black). The thermodynamic barriers of CPET reactions are overcome by applying a potential of -0.72 V (red). A progression on the abscisa indicate that a CPET reaction takes place, while intermediates of surface reactions are indicated in the same abscisa connected by a curved line (illustrating a TS). Bold numbers indicate the configuration identifier as described in the main text and illustrated in **Figure 5b**.

6.5. Boltzmann probability distributions of *CHO and *CO surface species

For a discrete collection of M configurations at a certain thermodynamic temperature, $\beta = 1/k_BT$ (in this case T = 298 K), the probability of a certain state (p_i) is proportional to its energy (E_i) in the following way:

$$p_i \propto e^{-\beta E_i}$$

The comparison of the probability of two states can be easily made using the Boltzmann factor, which is computed as the ratio of probabilities of the two states and depends only on their energy difference:

$$\frac{p_i}{p_j} = e^{\beta(E_j - E_i)}$$

We wish to determine the Boltzmann factor of two systems, namely configurations **5** and **6** (**Figure 4** of the main text), to deduce their relative population with respect to the applied potential. In these two configurations, **Hist**–CO is co-adsorbed with either *CO (**5**) or *CHO (**6**). The stoichiometry is maintained by adding (H^++e^-) to **5**, with a contribution to the energy approximated to that of ½H_2 (gas-phase), according to the computational hydrogen electrode (**SI Section 6.1**).

Figure S6.4 shows how the relative Gibbs free energy of configuration **6** (*CHO) changes with respect to that of **5** at various applied potentials. The two lines cross at the -0.72 V, indicating the potential above which *CHO population is expected to be higher than *CO, assuming no kinetic and diffusion limitations. This rough estimate indicates that after the applied potential is increased to 0.84 V, the *CHO population outnumbers that of *CO by a factor of 100.

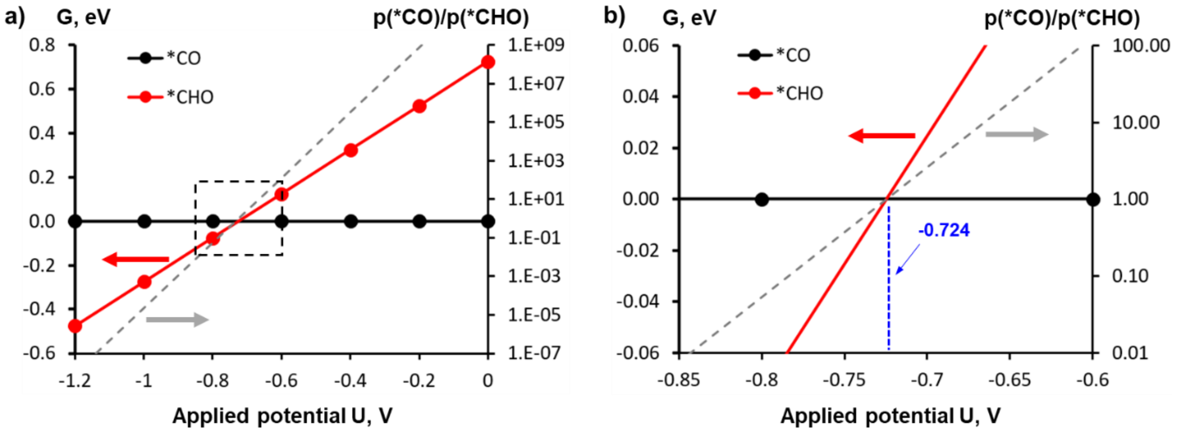

Figure S6.4: (a) Relative Gibbs free energy of configurations **5** and **6** at various applied potentials (black and red lines), with reference to **5**. The calculated Boltzmann factor between these two configurations, p(*CO)/p(*CHO), is also shown with a grey discontinuous line. (b) A closer look at the crossing point between the Gibbs free energy lines of *CO and *CHO. At potentials more negative than -0.724 V, *CHO species are expected to outnumber *CO.

6.6. Comparison of other solvation methods

To determine the effect of solvation and the solvation scheme used in the stabilization of surface species, we calculated the energy of various intermediates using three different solvation schemes (**Figure S6.5**). Two of the solvation schemes are semiempirical approaches (SE1 and SE2), where the solvation correction is based on the explicit solvation models. In SE1, solvation corrections of -0.50 eV, -0.25 eV, and -0.10 eV are added for *OH (surface hydroxyl), R-OH, and R-CO , respectively. This approach was implemented by Peterson et al. and exploited previous findings that *OH and R-OH are stabilized differently by liquid water. They also calculated the stabilization of *CO using an explicit water solvation model. Albeit simple, this approach has been used widely in the literature to model solvated intermediates.[22-26] SE2 is another semiempirical approach by Peng et al.,[27] where surface models with explicit water molecules were used to determine the extent of stabilization due to solvation for a specific intermediate (e.g., *CO, *COH, *OCCO, *OCCOH, etc.). Analogously to SE1, the estimated solvation correction is added to the energy of the corresponding intermediate. The third solvation correction scheme involves implicit solvation (IS). We used VASPsol implementation,[28,29] which accounts for the solvent as a continuous medium. The relative permittivity of the solvent (water) was fixed to 80 and we used a width of dielectric cavity of 0.6.

As shown in **Figure S6.5**, we explored how typical intermediates are stabilized by various solvation correction schemes. The intermediates are stabilized by -0.19 eV (SE1), -0.17 eV (SE2), and -0.22 eV (IS) in average with respect to the calculations in vacuum with a consistent standard deviation of 0.09 eV. The three approaches show same varying trends and similar results for the solvent effect. Therefore, we used the SE1 approach for all calculated intermediates in this work.

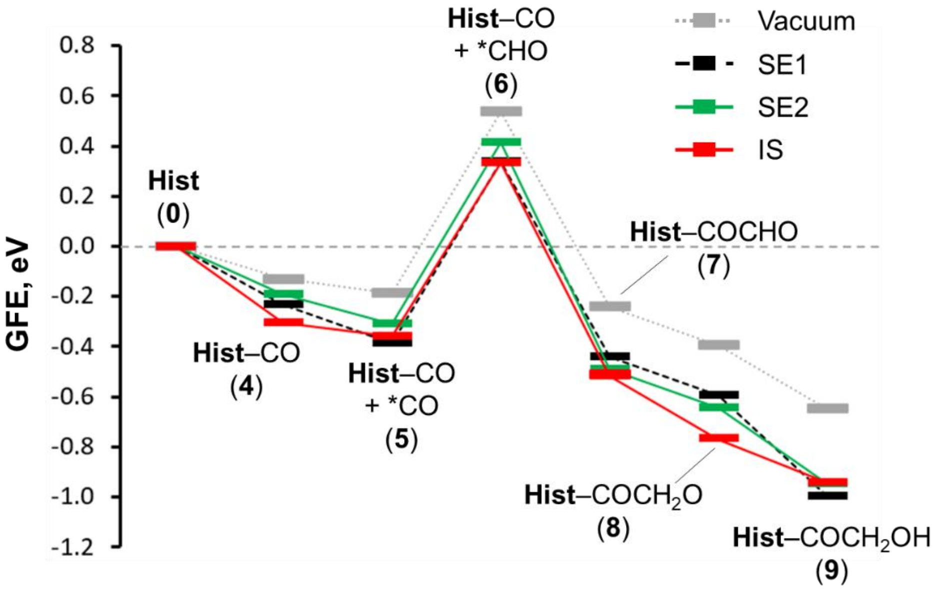

Figure S6.5: Gibbs free energy diagram of selected intermediates during CO_2RR to C_2H_4 through the histidine-assisted mechanism (**Figure 5** of main text) calculated with three solvation schemes. SE1 and SE2 are two semiempirical approaches, while IS values are calculated through the implicit solvation method as implemented in VASPsol. The baseline in grey represents the Gibbs free energy without considering solvation correction (vacuum). The configuration identifiers are indicated with bold numbers and follows the sequence of **Figure 5** on main text.

6.7. Other pathways explored

Paths to C1 products on Cu(100). Two reaction paths for the formation of CH_4 and one path for CH_3OH were explored over the Cu(100) for reference. **Figure S6.6** shows the relative Gibbs free energies of the calculated intermediates in such reaction pathways. The three pathways start with *CO, the recognised common intermediate during CO_2RR. The formation of methane can occur through the *CHO (blue line) or *COH (black line) intermediates, where these steps represent the rate-limiting steps in each pathway. The *CO→*CHO transformation is endergonic by 0.57 eV, more favourable than forming the COH* intermediate by 0.08 eV in qualitative agreement with other DFT calculations.[30] The following coupled proton-electron transfers are downhill in the formation of CH_4 through *CHO (blue line), except for the last protonation of *OH to H_2O, which is slightly less endergonic than the *CO→*CHO transformation (by 0.04 eV). Therefore, the reaction channel to produce CH_4 through the CHO* intermediate opens at 0.57 V of applied potential.

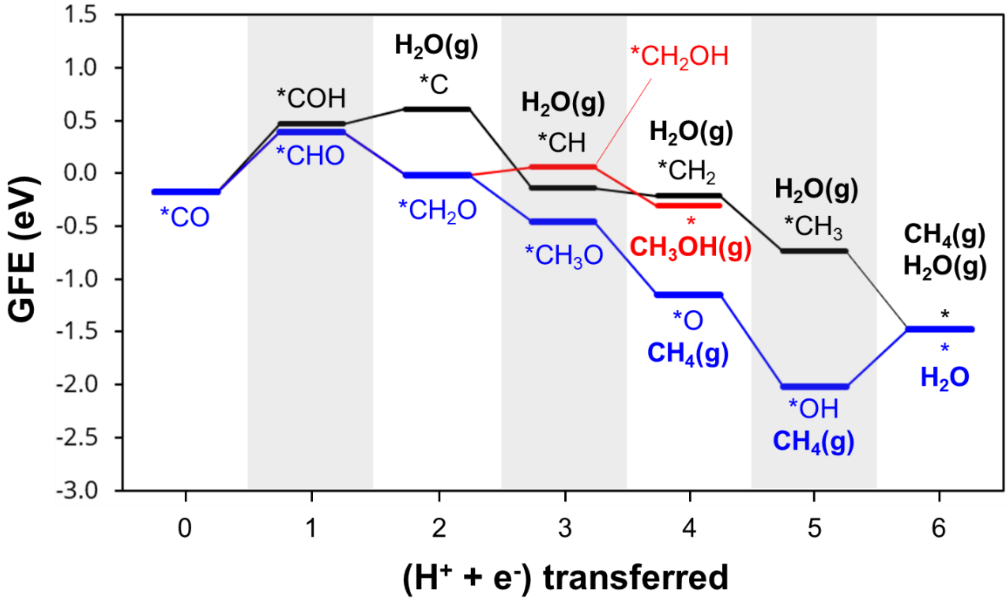

Figure S6.6: Multiple pathways to methane on Cu(100). Evolved products/by-products are marked in bold.

CH_4 formation in a histidine-assisted mechanism. We explored the possibility of the CH_4 formation with the assistance of histidine of (**Figure S6.7**). We will follow the convention discussed in the main text, in which reaction intermediates are indicated by bold numbers and reactions are discussed in the **A**→ **B** form (**A** and **B** being two consecutive intermediates). The histidine-Cu complex (**Figure S6.2b**) is denoted by the "**Hist**" label in the discussion that follows.

Analogous to the C_2H_4 formation pathway aided by histidine (**Figure 4** of the main text), the **Hist**-CO intermediate is formed after a series of surface and CPET reactions, **Figure 4** of main text. Subsequently, the progressive protonation of the **Hist**-CO leads to **Hist**-CH_2O (**Figure S6.7**, $\Delta G_{\mathbf{4\rightarrow17}}$ = -0.52 eV and $\Delta G_{\mathbf{17\rightarrow18}}$ = 0.28 eV). The following C–N bond breaking releases *CH_2O from the histidine fragment ($\Delta G_{\mathbf{18\rightarrow19}}$ = 0.25 eV and activation barrier of 0.99 eV), which has the highest barrier during the histidine-assisted CH_4 formation. Finally, the generated *CH_2O intermediate undergoes sequential protonation-deoxygenation-pronation to yield CH_4 (**19**→**20**→**21**).

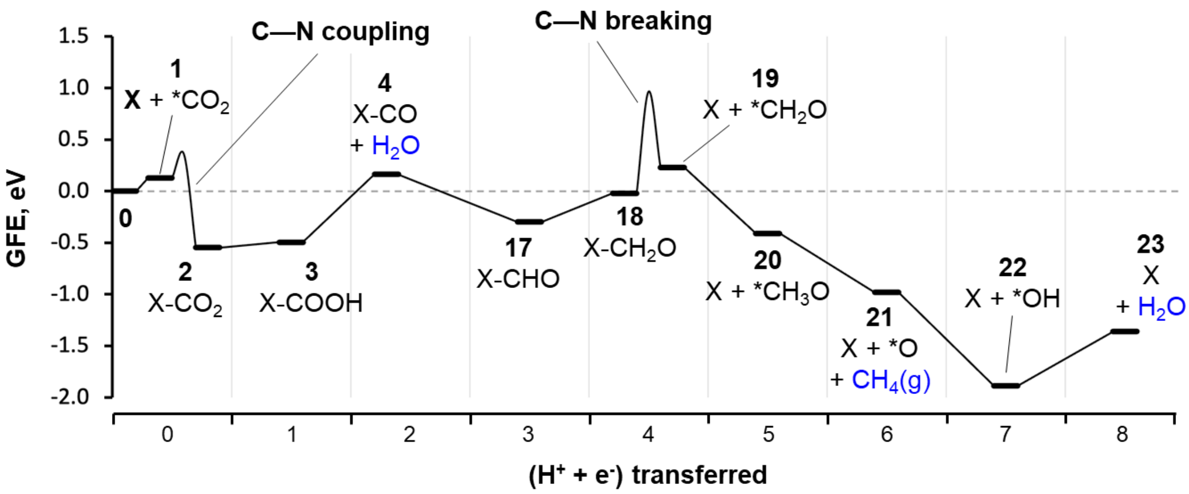

Figure S6.7: Gibbs free energy diagram for the histidine-assisted transformation of CO to CH_4. Histidine molecule is represented by a blue "**X**". The intermediates appearing in the same abscissa are linked through surface reactions, with no electrochemical step involved. Bold numbers are intermediate's identifiers, as displayed in **Figure 5b** of the main text. Products/byproducts generated during the reaction are shown in bold blue labels.

6.8. C-C coupling alternatives

On bare Cu(100). We tested various alternatives of the C–C coupling reaction that leads to the formation of C_2 products. In a reference calculation, the free energy of reaction for coupling two adsorbed *CO on the bare Cu(100) is endergonic by 0.96 eV alone, indicating a difficult process (**Figure S6.8a**). The free energy barrier of the process is 1.31 eV. This agrees with Montoya et al.[31] where the CO dimerisation is endothermic by 0.90 eV when no solvent and electric field are applied. Upon adding a charged water layer (with a solvated cation), the process becomes slightly exergonic, also reducing the CO* dimerisation barrier.

The *CO–*CHO occurring on the bare Cu(100) is endergonic by 0.10 eV, and the activation free energy is 0.63 eV (**Figure S6.8b**). Although it may represent a faster process compared to the direct CO dimerisation on bare Cu(100) described in the paragraph above, the C–C coupling of *CO and *CHO on bare Cu(100) is still less favourable than the one when the histidine molecule assists in the reaction. As described in the main text, the C–C coupling in the histidine-assisted mechanism from *CO and *CHO is exergonic by 0.78 eV and has a barrier of only 0.34 eV.

Comparatively, the C–C coupling of two *CO on bare Cu(100) was previously calculated to be endergonic by 0.96 eV and with a kinetic barrier of 1.18 eV.[32] The C–C coupling between co-adsorbed *CO and *CHO species had been studied computationally before over Cu(100)[33] and the energy barrier was found to be slightly higher than for the *CO dimerization at 0 V.

We also tested if there is an effect from the Cu adatom used to simulate the roughness of the Cu_2O-derived metallic Cu surface. For this purpose, we added a Cu atom on the most favourable 4-fold position, leading to a surface model analogous to the histidine/Cu(100) model but without adsorbed histidine. Before the C–C coupling between two *CO, one *CO binds the undercoordinated Cu adatom through the C atom and the other *CO sits on a bridge site (**Figure S6.8c**). As a result, the C–C coupling is endergonic by 0.97 eV. The free energy barrier was not calculated in this case, since the free energy change for the reaction already indicates an unfavourable process in comparison with other alternatives.

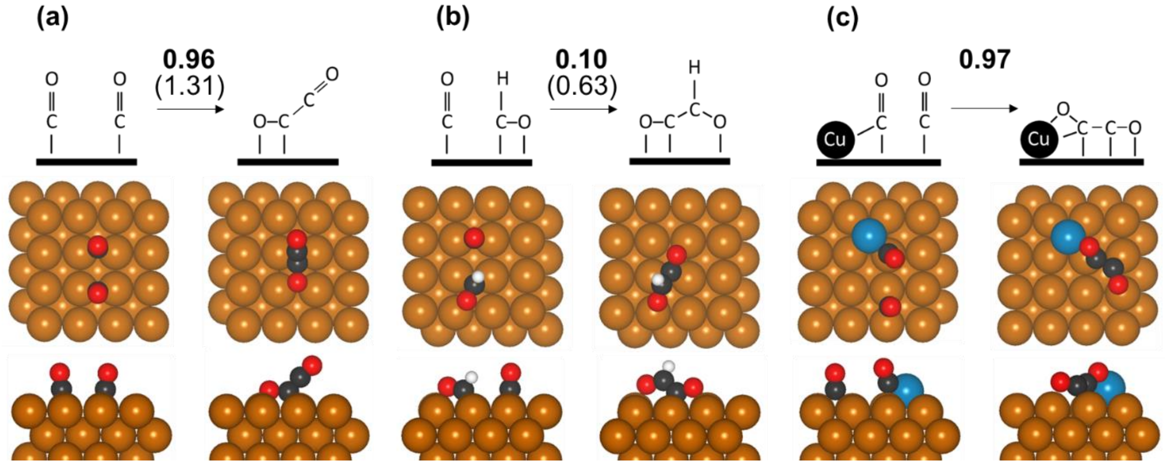

Figure S6.8: Schematic drawing of the explored possibilities for the C-C coupling on Cu(100) surface. (a) *CO–*CO coupling. (b) *CO–*CHO coupling. (c) *CO–*CO coupling on modified Cu(100) surface with one additional Cu adatom on 4-fold position. Only initial and final states are represented. The corresponding structures are also shown at the bottom (top and side view). Bold numbers indicate the Gibbs free energy change for the reaction, while the free energy barriers are shown in parenthesis.

6.9. Alternative pathways during the histidine-assisted hydrogenation of CO_2

We studied alternative pathways after the formation of the Hist-CO intermediate, X-CO intermediate in **Figure 6.9**. We favoured pathways with exothermic reactions. On the most favourable pathway forming C_2H_4 (orange substrate, **Figure 6.9**), the only endothermic reactions are the C—C coupling reaction (ΔE = 0.38 eV) and the desorption of C_2H_4 (ΔE = 0.77 eV). Other pathways lead to less stable intermediates. Similarly, we sampled the stability of several alternatives during the formation of CH_4 (grey substrate, **Figure 6.9**). We identified that the most favourable pathway proceeds via the X–CO → X–CHO → X–CH_2O sequence, followed by the detachment of *CH_2O from the histidine complex.

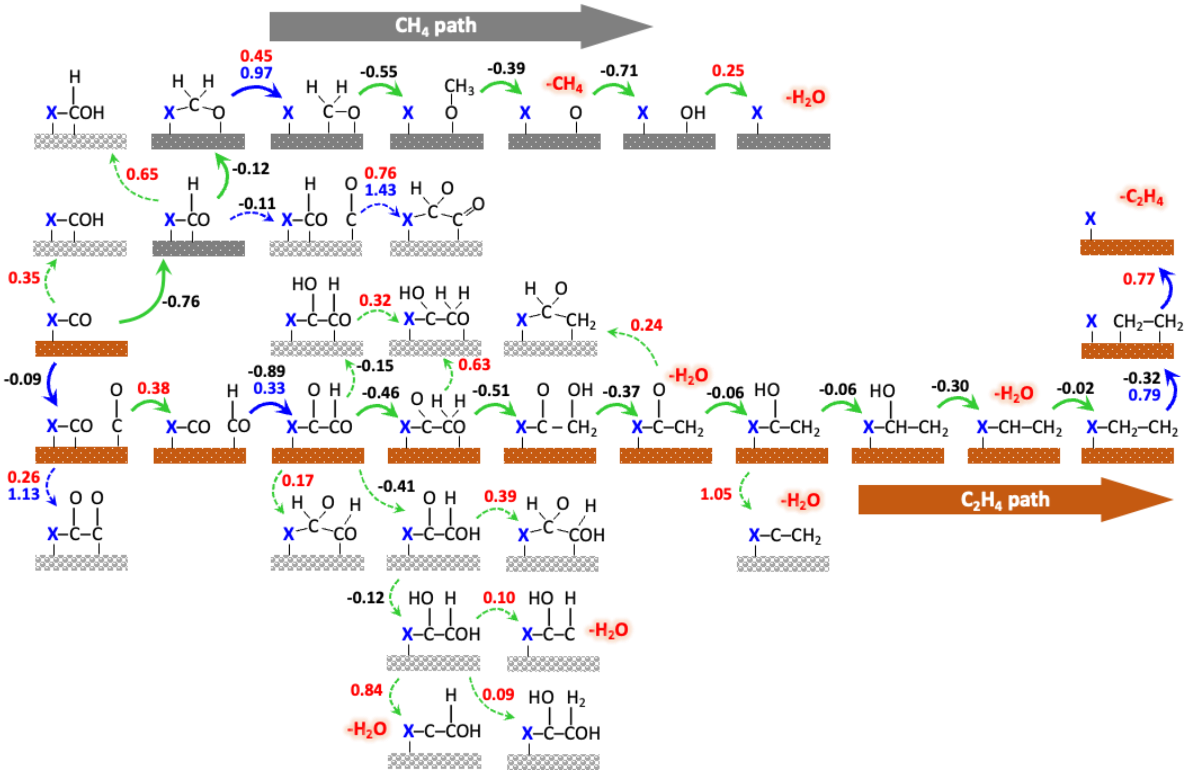

Figure 6.9. Studied intermediates for the transformation of the Hist-CO complex (a blue X denotes the histidine fragment) to either CH_4 (dark grey) or C_2H_4 (orange). The intermediates connected via reactions involved in the two main pathways are indicated with solid arrows, color-coded as blue for surface reactions and green for coupled proton-electron transfer reactions. Alternative reactions are also displayed with dotted arrows and their corresponding configurations. The reaction energies are indicated in black (exothermic) and red (endothermic) numbers, while the activation energies for surface reactions are shown in blue numbers below the reaction energies. Desorbing species during a reaction are indicated with labels in red font.

7. Electrochemical Impedance Spectroscopy

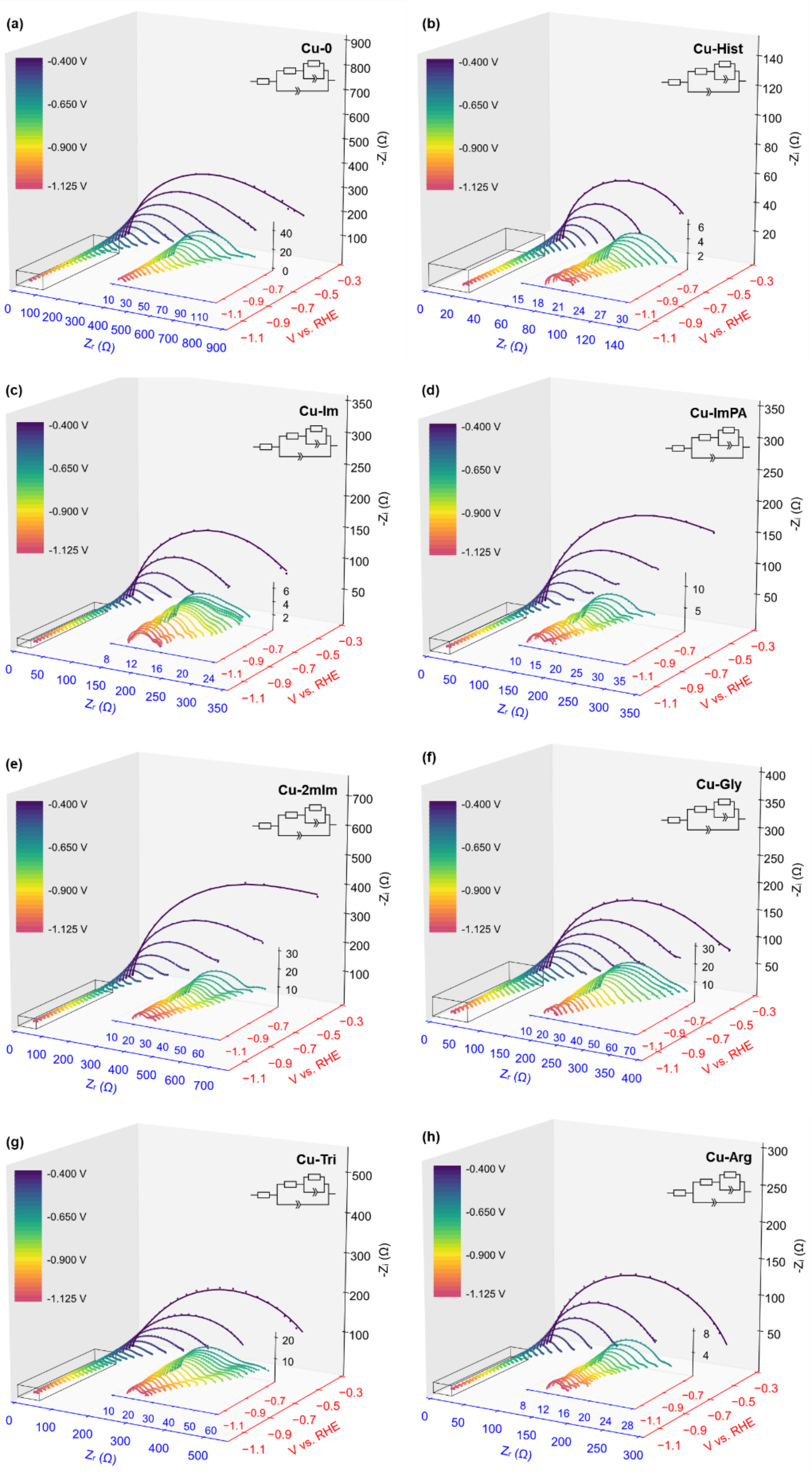

Figure S7.1: Nyquist plots from -0.400 to -1.125 V for (a) Cu-0, (b) Cu-Hist, (c) Cu-Im, (d) Cu-ImPA, (e) Cu-2mIm, (f) Cu-Gly, (g) Cu-Tri, (h) Cu-Arg. Inset graph is a magnification of the Nyquist plot at more cathodic DC potentials (marked with wireframe)

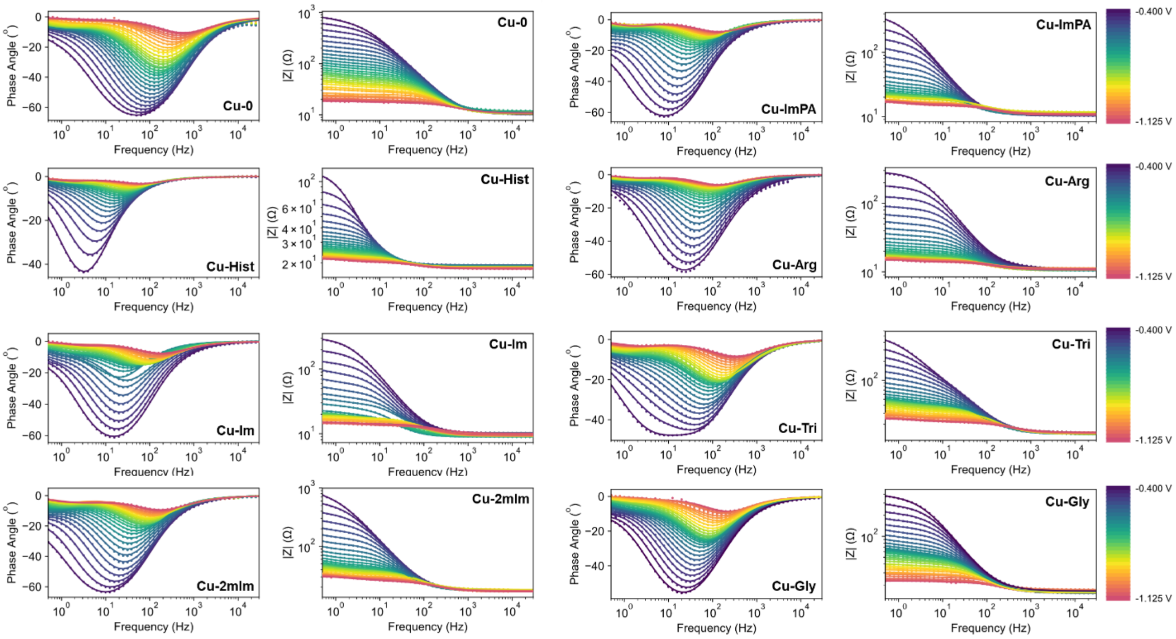

Figure S7.2: Bode plots for all samples from EIS measurement performed at -0.400 to -1.125 V DC voltages.

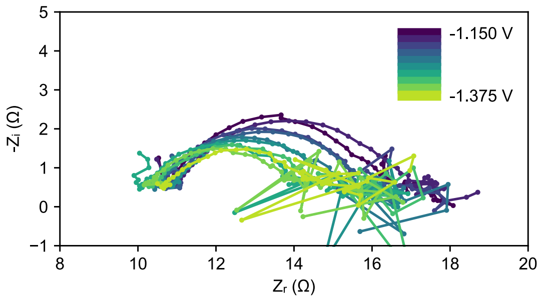

Figure S7.3: EIS measurement attempts at very cathodic DC potentials between -1.150 to -1.375 V

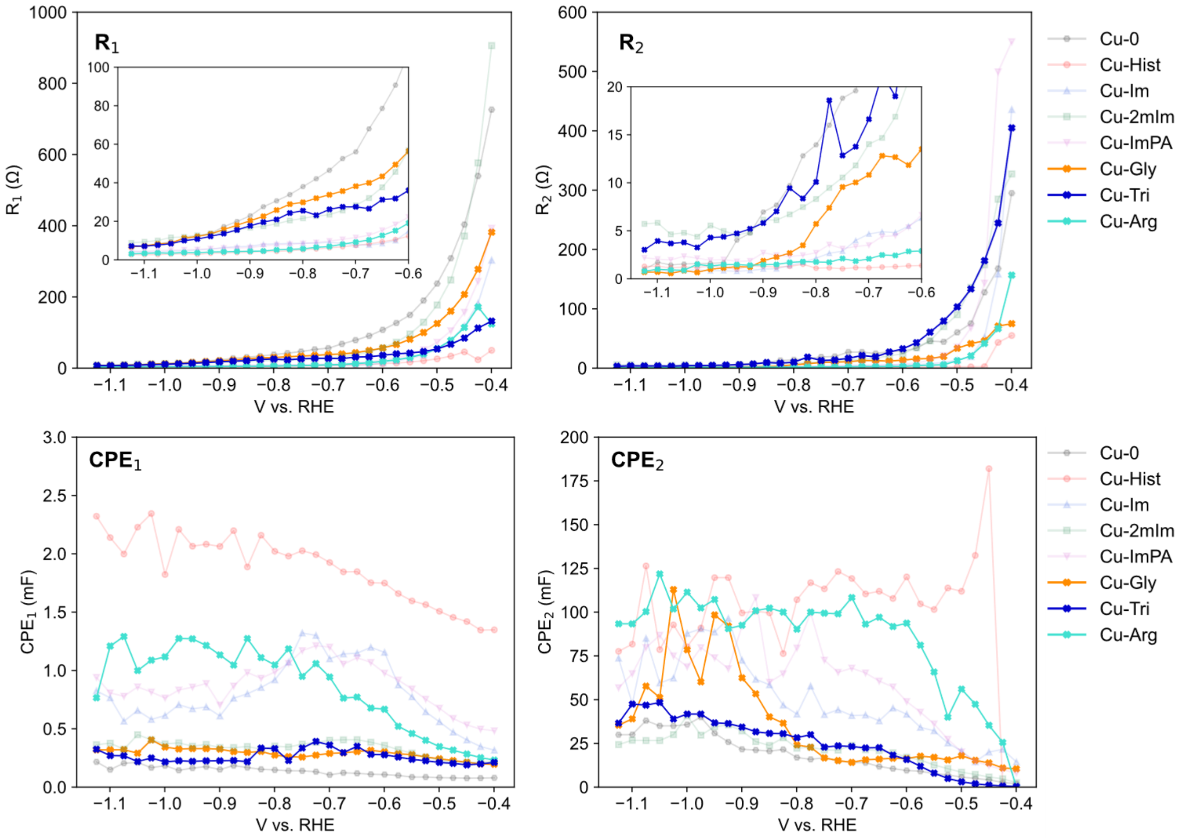

Figure S7.4: EIS derived parameters of additional molecules (Cu-Arg, Cu-Gly, and Cu-Tri). The rest of the data was dimmed for clarity.

8. Modified Pulse Voltammetry (mPV)

The transient current response originating from surface charge was recorded for the entire duration of the pulse (t >4 ms), at varying DC voltages between -0.5 to -1.6 V which is relevant to the CO_2RR operating voltage of our catalysts. We note that good current profile without significant noise can be collected even at very cathodic potential despite gas evolution bubbles, as we provide long hold time in between pulses (20 s) to allow for sufficient charge discharge/relaxation and current data averaging (**SI Figure S8.1, S8.4, S8.5, S8.6)**

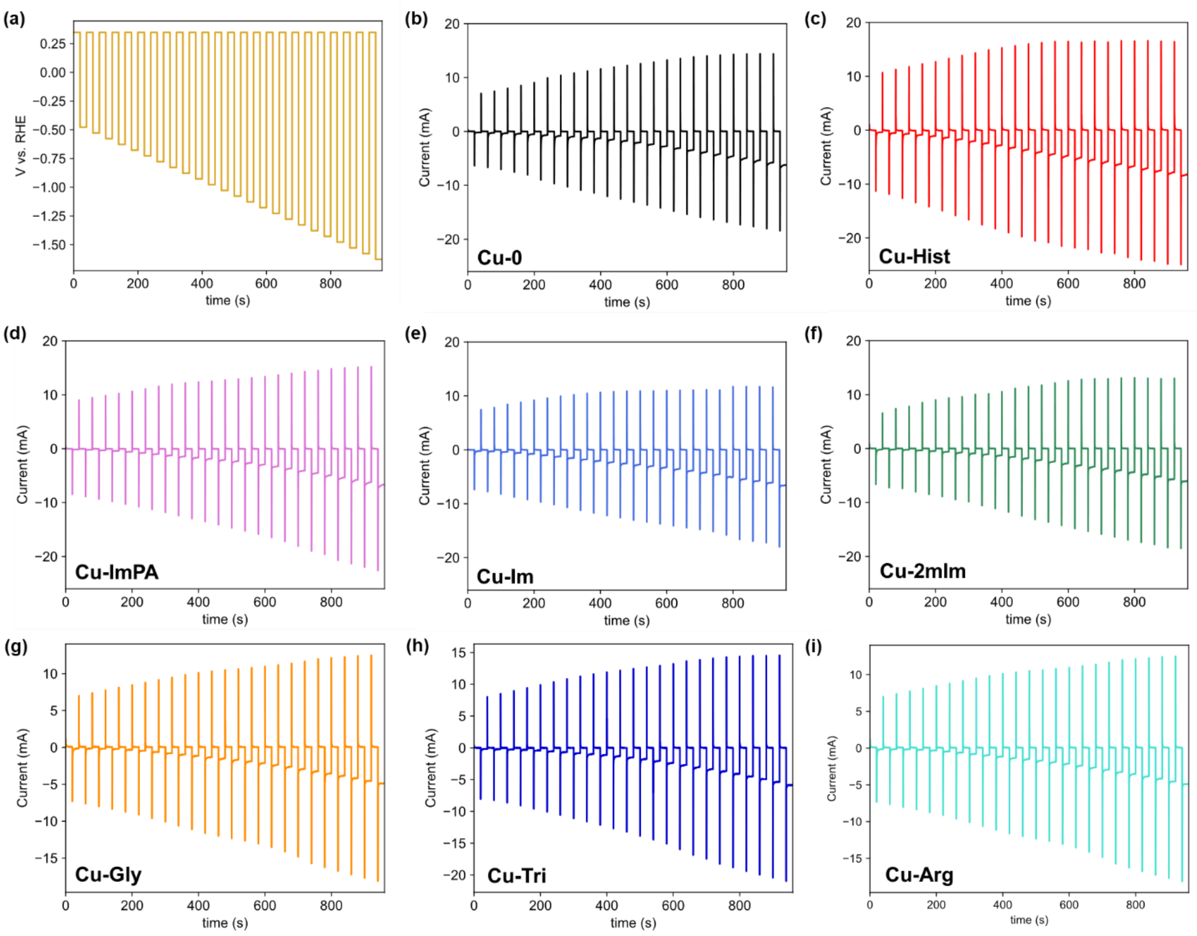

Figure S8.1: (a) Representative stepwise potential profile applied to the working electrode for mPV experiments. (b-i) Pulse responses of Cu samples with various surface functionalisation.

8.1. Representative Plots of Fitted Anodic Current Decay with Various Functions

The anodic decay profile was used as opposed to the cathodic response to reduce the convolution with the catalytic current, and to minimize possible diffusion-limitations arising from adsorption of intermediates or desorption of gaseous product bubbles.

Five different functions that are commonly used to estimate transient current decay in electrochemical systems were considered.[34] We found that the anodic charge decay cannot be fit to just one function, suggesting a convolution of at least two processes occurring on the catalyst surface.

A good fit can be obtained by combining a 2nd order rate kinetic function up to ~0.4-0.5 s, and either a diffusion limited function (**Figure S8.2c-d**) or first order rate kinetic function (**Figure S8.2f)**. The 2nd order – 1st order combination fits better than Cottrell, thus the combination was used for the fitting of all transient anodic decay in this study. We attribute the 2nd order rate kinetic to desorption process, and the 1st order is background process, which may include hydration or oxidation. Brief description of the functions tested are discussed below.

<u>Zero order kinetics (linear)</u> **<u>(Figure S8.2a)</u>**

$$C = k_{0th}t$$

$$I = \frac{dQ}{dt} \propto \frac{dC}{dt} = k_{0th}$$

where k is the rate constant.

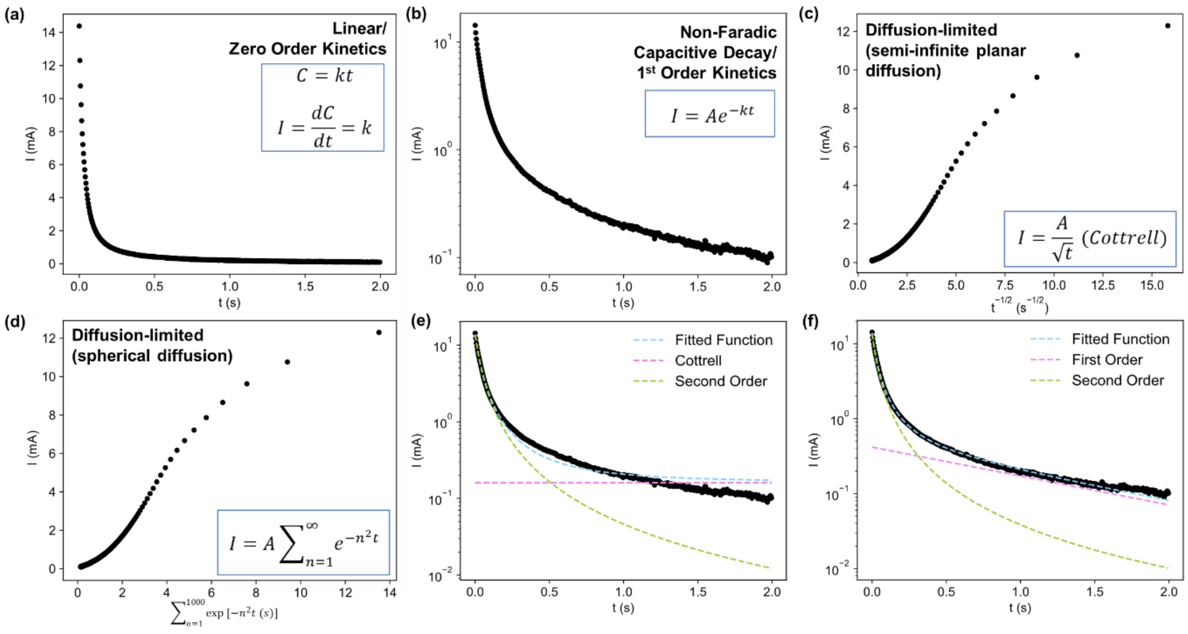

Figure S8.2: Linearization of transient anodic current response of mPV measurements with respect to various commonly used equations. (a) Linear x/y-scale, (b) Logarithmic y-scale, (c) plot of current against square-root of time, in line with diffusion-limited reactions as described by the Cottrell equation, (d) plot of linearization of diffusion-limited reaction for spherical diffusion. (e-f) Example of fitting of the transient anodic current response using a combination of (e) second order kinetics and Cottrell equation and (f) second order and first order kinetics.

Double layer capacitance decay or first order kinetics (**Figure S8.2b**)

The non-Faradaic double layer capacitance typically follows a discharge profile described by the equation (3) below.

$$I = \frac{\Delta E}{R_s} \exp\left(-\frac{t}{R_s C_{dl}}\right) \quad (3)$$

where R_s is the series resistance, C_{dl} is the double layer capacitance and ΔE is the potential step.

Similar to double layer capacitance decay, desorption that follows first order kinetics also follows exponential decay as follows:

$$C = C_{0_{1st}} e^{-k_{1st} t} \quad (4)$$

where k is the rate constant and C_0 is the initial concentration of the charged species.

Assuming that the desorption of adsorbed species is accompanied by an electron transfer, the amount of charge (Q) directly reflects the concentration of the adsorbed species (C).

The expected current response may then be modelled as follows.

$$-I = \frac{dQ}{dt} \propto \frac{dC}{dt} = -C_{0_{1st}} k_{1st} e^{-k_{1st} t} \quad (5)$$

Considering that a relatively slow sampling time of 4 ms is adopted in this work, the double layer capacitance is unlikely to contribute significantly to our transient anodic current profile.[35] Further we do not see clear linear relation on log current plot against time at t <0.5 s (**Figure S8.2b**). The log transient anodic decay profile only starts to follow a linear trend from around t >0.5 s, suggesting that a first order kinetic may be dominating at longer timescale.

Cottrell diffusion (planar) and spherical diffusion **(Figure S8.2c-d)**

Diffusion-limited current response is commonly modelled using the Cottrell equation for semi-infinite planar diffusion:

$$I = \frac{nFAC_0\sqrt{D}}{\sqrt{\pi t}} \quad (6)$$

where n is the number of electrons transferred, F is the Faraday's constant, A is the surface area of the working electrode, D is the diffusivity of the species and C_0 is the initial concentration of the species.

On particulate samples, where the diffusion plane is better represented by spherical diffusion, the current response for spherical diffusion for a small particle is given by:

$$I = \frac{2FAD\Delta C}{a} \sum_{n=1}^{\infty} \exp\left(-\frac{n^2\pi^2 Dt}{a^2}\right) \quad (7)$$

where F is the Faraday's constant, D is the diffusion coefficient, A is the geometric area of the working electrode, a is the radius of the particle and ΔC is the change in ion concentration in the host structure.

Plotting the transient anodic current response against $\sqrt{t}$ (**Figure 5.2c**) and $\sum_{n=1}^{\infty} \exp(-n^2 t)$ and (**Figure 5.2d**) displayed poor linearity at low t values, but some linearity was observed at t > 0.7 s.

Second order kinetics

Reactions with second order reaction kinetics may be described by the equation (8) below, where C is the concentration, C_0 is the initial concentration and k is the rate constant.

$$\frac{1}{C} - \frac{1}{C_{0_{2nd}}} = k_{2nd}t \qquad (8)$$

where k is the rate constant and C_0 is the initial concentration of the charged species.

Assuming that the desorption of adsorbed species is accompanied by an electron transfer, the amount of charge (Q) directly reflects the concentration of the adsorbed species (C).

Since the change in concentration ($\frac{dC}{dt}$) is parallel to $\frac{dQ}{dt}$, and $I = \frac{dQ}{dt}$, the current profile may be modelled with the equation (9) below.

$$\frac{dC}{dt} = \frac{d}{dt}\left(\frac{1}{C_{0_{2nd}}} + k_{2nd}t\right)^{-1} = \frac{k}{\left(C_{0_{2nd}} + k_{2nd}t\right)^{2}} \qquad (9)$$

8.2. Plots of All Fitted Anodic Current Decays with Combined Function

Based on findings on **SI section 5.1**, the best fit for our transient anodic pulse is achieved by adding a background 1st order decay to a second order kinetics decay. The overall function used to fit the anodic decay is as follows:

$$F(t) = \frac{k_{2nd}}{\left(C_{0_{2nd}} + k_{2nd}t\right)^2} + C_{0_{1st}} e^{-k_{1st}t} \quad (10)$$

For simplicity, $C_{0_{2nd}}$ is denoted as C_{2nd} henceforth (also in the main text). The first term represents a second order decay kinetic that dominates to t ≈ 0.4-0.5 s, and the second term is a first order decay kinetic that dominates from t >0.5 s (**Main Text Figure 4b, SI section 8.1**). The first order decay can be attributed to slower background processes under OCP, such as hydration or oxidation. The second order term should reflect the cumulative desorption of species on the catalyst surface.

The fittings suggest a Faradaic process following a 2nd order reaction kinetics dominating from t = 0 to t = 0.5 s, which accounts for most of the anodic decay. A background process with 1st order kinetics is also observed, dominating from t = 0.5 s, though it accounts for only a small fraction of the total current (**Figure S8.3**). This is consistent with the linearity of the graph after t = 1s for 1st order kinetics (**Figure S8.2b)**. Good fits across all samples are obtained when the anodic current decays are fitted with the combined function above (**Figure S8.4**)

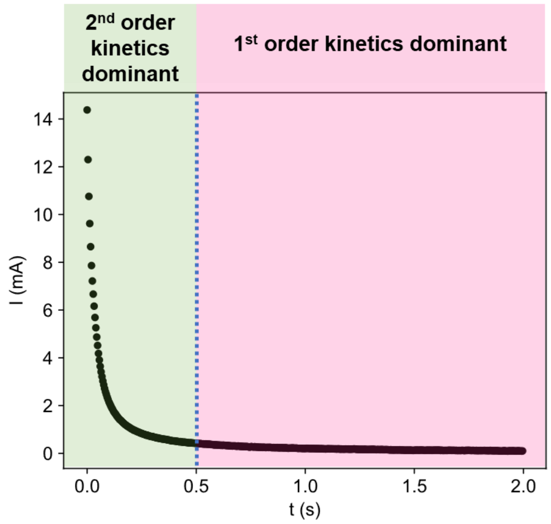

Figure S8.3: Rough breakdown of dominant processes accounting for the anodic decay.

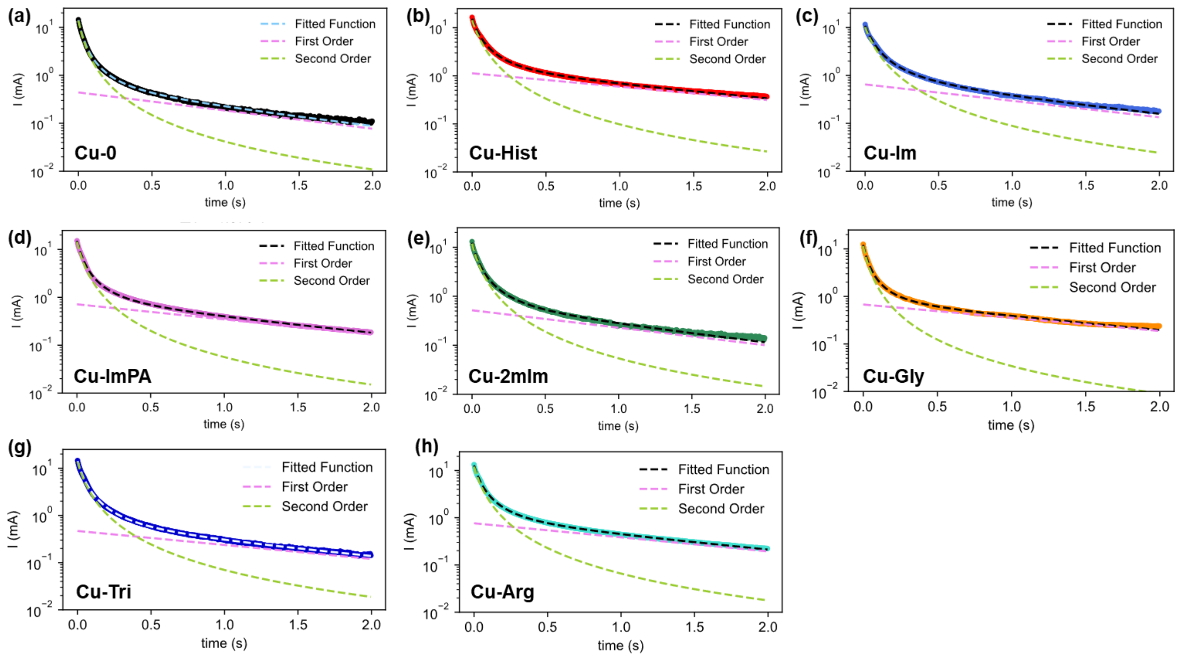

Figure S8.4: Example fittings for transient anodic mPV current profiles for all samples, at ΔV=1.875 V. Good fits were obtained for all samples and across all decays used.

We fit the mPV current decay of all samples from ΔV of 1.2 to 1.9 (equivalent to *approx.* -0.9 to -1.6 V cathodic potentials) and display the C_{2nd} and k_{2nd} coefficient term in **Figure S8.5**. C_{2nd} describes the initial current (at t = 0), while k_{2nd} parameter describes the current decay rate. As the current represents the electrons transferred as adsorbed species leave the catalyst surface, the parameter C_{2nd} can be interpreted as the initial adsorbed species concentration, and k_{2nd} how quickly the adsorbed intermediates on the catalysts' surface desorb upon switching the cathodic bias to the "off" (or OCP) state.

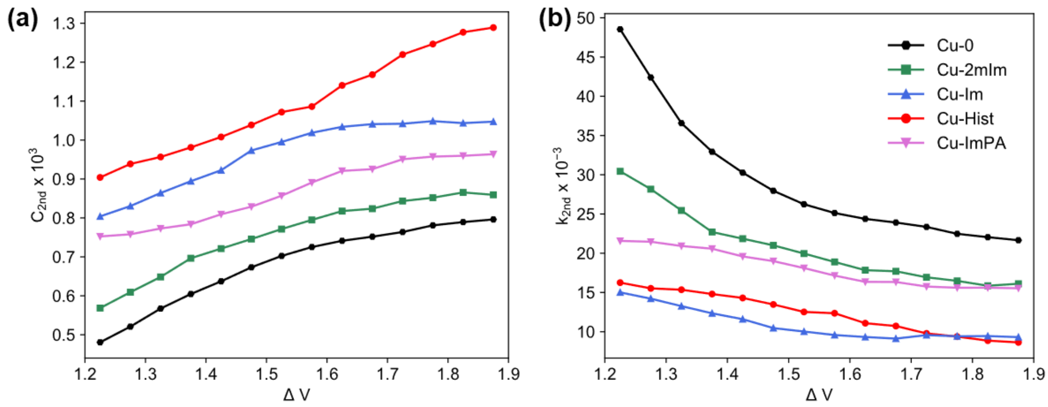

Figure S8.5: 2nd order decay fittings result of transient anodic current profiles measured during mPV experiments (a) C_{2nd}, and (b) k_{2nd} for Cu-0, Cu-2mIm, Cu-Im, Cu-Hist, and Cu-ImPA.

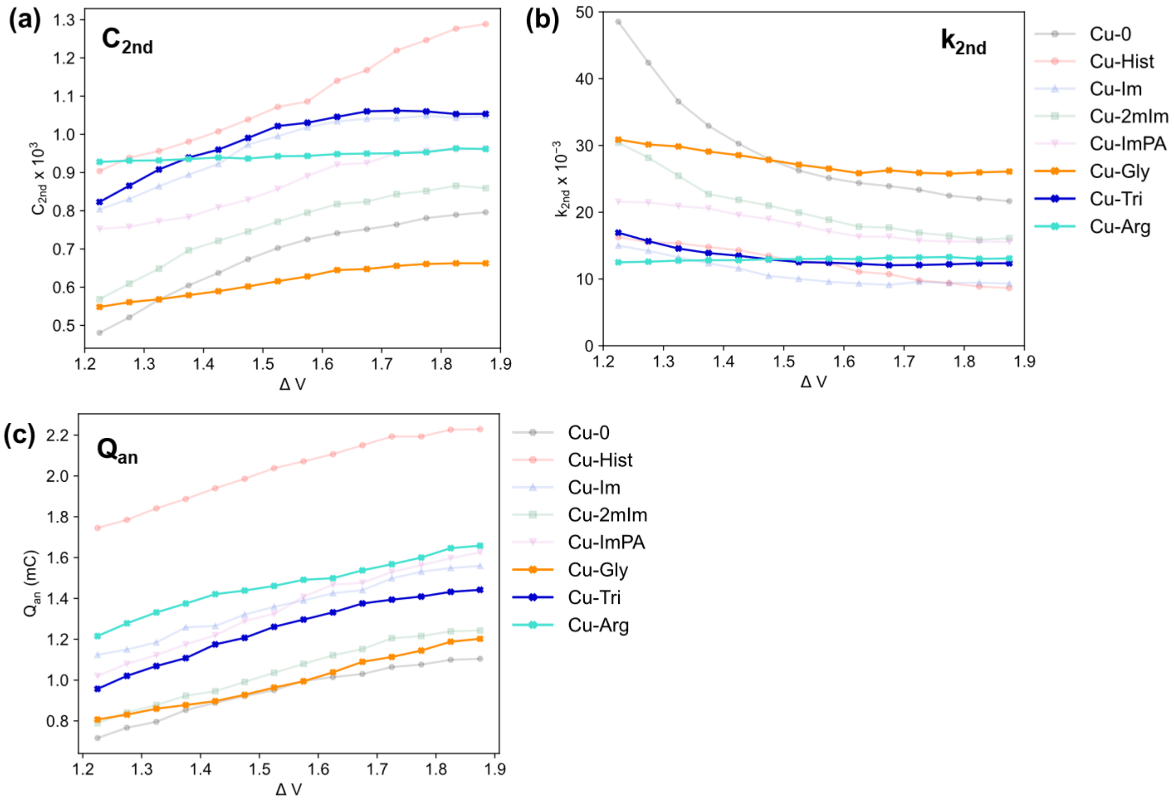

Figure S8.6: mPV derived parameters of validation molecules (Cu-Gly, Cu-Arg and Cu-Tri). (a) C_{2nd}, (b) k_{2nd}, and (c) Q_{an}. Data from the rest of the functionalisation was dimmed for clarity.

9. Parameter Correlations

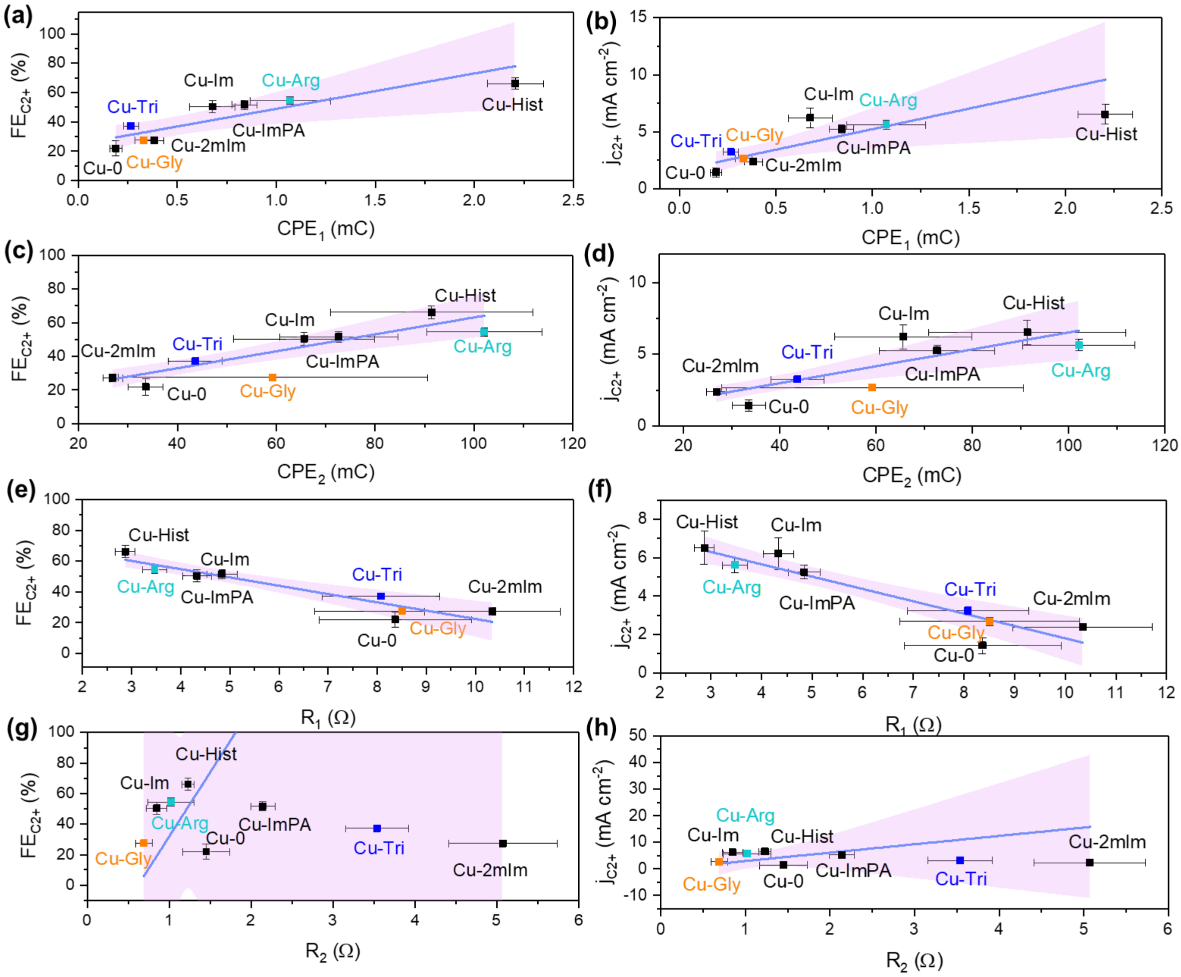

Figure S9.1: Correlation plot between EIS derived parameters: (a-b) CPE_1, (c-d) CPE_2, (e-f) R_1, (g-h) R_2, to FE_{C2+} and j_{C2+} at -1.6 V. Error bars represent standard deviation from 3 independent measurements. Purple lines are linear York fitting that consider both Y and X errors. Pink shaded area are 95% confidence band intervals of the linear fit.

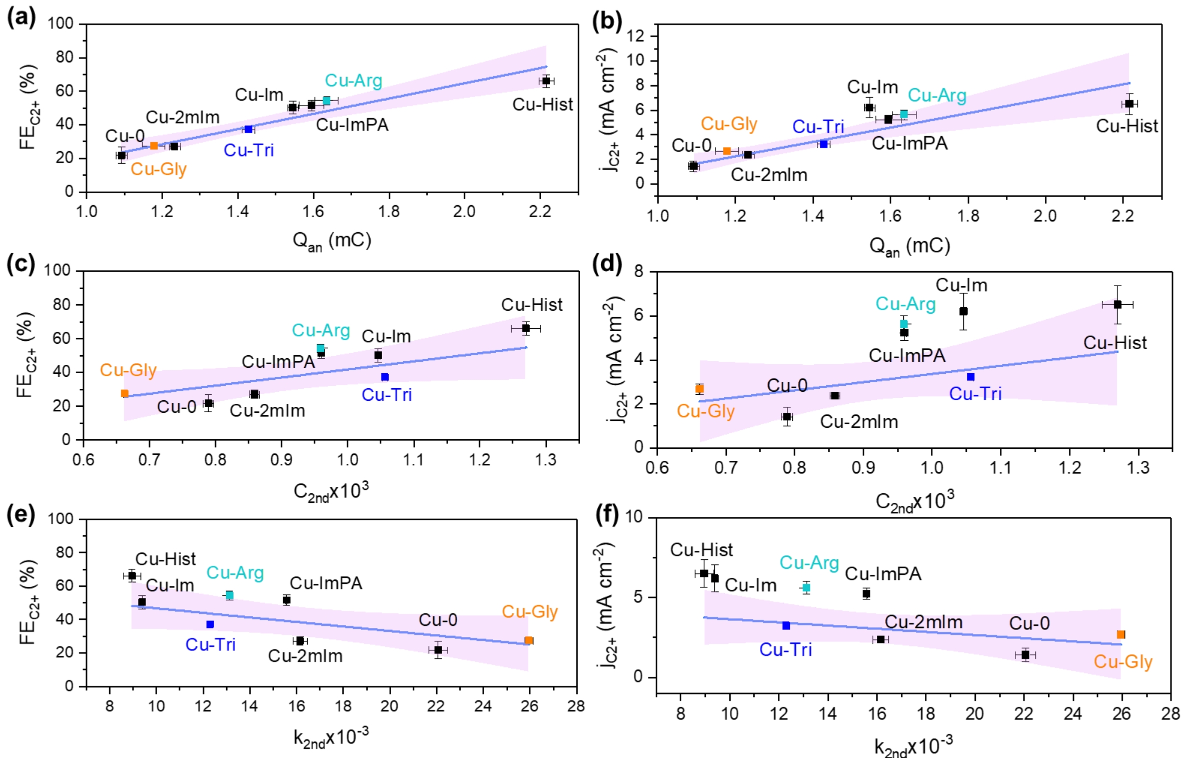

Figure S9.2: Correlation plot with mPV derived parameters (a-b) Q_{an}, (c-d) C_{2nd}, (e-f) k_{2nd} and FE_{C2+} (j_{C2+}). Error bars represent standard deviation from 3 independent measurements. Purple lines are linear York fitting that consider both Y and X errors. Pink shaded area are 95% confidence band intervals of the linear fit.

10. Performance Benchmarking

Table S10.1: Comparison of organic functionalised Cu catalysts for CO_2RR in the literature

Catalyst	FE_{C2+} (%)	V (*vs.* RHE)*	Maximum Stability Demonstrated (h)	Electrolyte	Total -j (mA cm^{-2})	Ref.
**Molecular-functionalized/modified catalysts**						
Histidine-functionalized Cu_2O-derived Cu(0)	76.64	- 2.0	48	0.1 M $KHCO_3$	15.41	This work
Glycine-functionalized Cu nanowires	33.8	~ -1.25	12	0.1 M $KHCO_3$	~ 14	[36]
Benzimidazole-functionalized Cu foils	76.68	-1.09	10	0.1 M $KHCO_3$	~ 25	[2]
1-octadecanethiol-treated Cu dendrites	74	~ -1.3	-	0.1 M $CsHCO_3$	30	[37]
Polyacrylamide-modified Cu foam	~32	-0.96	-	0.1 M $NaHCO_3$	60	[38]
Polyaniline-coated Cu nanoparticles	78.4	-1.13	20	0.1 M $KHCO_3$	34.7	[39]
CuO with fluorinated ethylene propylene binder	52	-1.1	-	0.1 M $KHCO_3$	37.4	[40]
**Other C_{2+}-selective catalysts**						
Cu_2O-derived Cu film	59.8	-0.98	-	0.1 M $KHCO_3$	31.2	[5]
Oxide derived Cu foam	55	-0.8	-	0.5 M $KHCO_3$	~ 11.5	[41]
Oxide-derived $Cu_{75}Zn_{25}$ nanocubes	59.4	-1.1	-	0.1 M $KHCO_3$	~ 15	[42]
Anodized Cu nanowire array	~60	-1.08	40	0.1 M $KHCO_3$	19.2	[43]
Iodine-modified nanostructured Cu	80	-0.9	22	0.1 M $KHCO_3$	~ 39	[44]
Electro-redeposited Cu	52	-1.2	-	0.1 M $KHCO_3$	~ 59.6	[45]
Plasma-activated Cu	~61.7	-0.9	-	0.1 M $KHCO_3$	~ 11	[46]

*For references that does not report the voltage in RHE, conversion was done based on information available on the reference.

11. References

1 Iijima, G. *et al.* CO_2 Reduction Promoted by Imidazole Supported on a Phosphonium-Type Ionic-Liquid-Modified Au Electrode at a Low Overpotential. *ACS Catal.* **8**, 1990-2000, doi:10.1021/acscatal.7b03274 (2018).

2 Zhong, S. *et al.* Efficient electrochemical transformation of CO_2 to C_2/C_3 chemicals on benzimidazole-functionalized copper surfaces. *Chem. Commun.* **54**, 11324-11327, doi:10.1039/C8CC04735A (2018).

3 Lau, G. P. S. *et al.* New Insights Into the Role of Imidazolium-Based Promoters for the Electroreduction of CO_2 on a Silver Electrode. *J. Am. Chem. Soc.* **138**, 7820-7823, doi:10.1021/jacs.6b03366 (2016).

4 Göttle, A. J. & Koper, M. T. M. Proton-coupled electron transfer in the electrocatalysis of CO_2 reduction: prediction of sequential vs. concerted pathways using DFT. *Chem. Sci.* **8**, 458-465, doi:10.1039/c6sc02984a (2017).

5 Handoko, A. D. *et al.* Mechanistic Insights into the Selective Electroreduction of Carbon Dioxide to Ethylene on Cu_2O-Derived Copper Catalysts. *J. Phys. Chem. C* **120**, 20058-20067, doi:10.1021/acs.jpcc.6b07128 (2016).

6 Feyer, V. *et al.* The Electronic Structure and Adsorption Geometry of l-Histidine on Cu(110). *J. Phys. Chem. B* **112**, 13655-13660, doi:10.1021/jp805671h (2008).

7 Anantharaj, S., Karthik, P. E. & Noda, S. The Significance of Properly Reporting Turnover Frequency in Electrocatalysis Research. *Angew. Chem. Int. Ed.* **60**, 23051-23067, doi:10.1002/anie.202110352 (2021).

8 Morris, G. A. & Freeman, R. Selective excitation in Fourier transform nuclear magnetic resonance. *J. Magn. Reson.* **29**, 433-462, doi:10.1016/0022-2364(78)90003-3 (1978).

9 Deng, Y., Handoko, A. D., Du, Y., Xi, S. & Yeo, B. S. In Situ Raman Spectroscopy of Copper and Copper Oxide Surfaces during Electrochemical Oxygen Evolution Reaction: Identification of Cu^{III} Oxides as Catalytically Active Species. *ACS Catal.* **6**, 2473-2481, doi:10.1021/acscatal.6b00205 (2016).

10 Mesu, J. G., Visser, T., Soulimani, F. & Weckhuysen, B. M. Infrared and Raman spectroscopic study of pH-induced structural changes of l-histidine in aqueous environment. *Vib. Spectrosc* **39**, 114-125, doi:10.1016/j.vibspec.2005.01.003 (2005).

11 Hu, G., Smith, K. H., Liu, L., Kentish, S. E. & Stevens, G. W. Reaction kinetics and mechanism between histidine and carbon dioxide. *Chem. Eng. J.* **307**, 56-62, doi:10.1016/j.cej.2016.08.066 (2017).

12 Ringe, S. *et al.* Understanding cation effects in electrochemical CO_2 reduction. *Energy Environ. Sci.* **12**, 3001-3014, doi:10.1039/c9ee01341e (2019).

13 Martusevičius, S., Niaura, G., Talaikytė, Z. & Razumas, V. Adsorption of l-histidine on copper surface as evidenced by surface-enhanced Raman scattering spectroscopy. *Vib. Spectrosc* **10**, 271-280, doi:10.1016/0924-2031(95)00025-9 (1996).

14 Deschamps, P., Kulkarni, P. P., Gautam-Basak, M. & Sarkar, B. The saga of copper(II)–l-histidine. *Coord. Chem. Rev.* **249**, 895-909, doi:10.1016/j.ccr.2004.09.013 (2005).

15 Shan, W. *et al.* *In Situ* Surface-Enhanced Raman Spectroscopic Evidence on the Origin of Selectivity in CO_2 Electrocatalytic Reduction. *ACS Nano* **14**, 11363-11372, doi:10.1021/acsnano.0c03534 (2020).

16 Peterson, A. A., Abild-Pedersen, F., Studt, F., Rossmeisl, J. & Norskov, J. K. How copper catalyzes the electroreduction of carbon dioxide into hydrocarbon fuels. *Energy Environ. Sci.* **3**, 1311-1315, doi:10.1039/C0EE00071J (2010).

17 Nørskov, J. K. *et al.* Origin of the Overpotential for Oxygen Reduction at a Fuel-Cell Cathode. *J. Phys. Chem. B* **108**, 17886-17892, doi:10.1021/jp047349j (2004).

18 Riffet, V. & Bouchoux, G. Gas-phase structures and thermochemistry of neutral histidine and its conjugated acid and base. *Phys. Chem. Chem. Phys.* **15**, 6097-6106, doi:10.1039/C3CP00043E (2013).

19 Franklin, L. M., Walker, S. M. & Hill, G. A DFT study of isolated histidine interactions with metal ions (Ni^{2+}, Cu^{2+}, Zn^{2+}) in a six-coordinated octahedral complex. *J. Mol. Model.* **26**, 116, doi:10.1007/s00894-020-04389-2 (2020).

20 Pavelka, M., Šimánek, M., Šponer, J. & Burda, J. V. Copper Cation Interactions with Biologically Essential Types of Ligands: A Computational DFT Study. *J. Phys. Chem. A* **110**, 4795-4809, doi:10.1021/jp056868z (2006).

21 Arán-Ais, R. M. *et al.* Imaging electrochemically synthesized Cu_2O cubes and their morphological evolution under conditions relevant to CO_2 electroreduction. *Nat. Commun.* **11**, doi:10.1038/s41467-020-17220-6 (2020).

22 Bagger, A., Arnarson, L., Hansen, M. H., Spohr, E. & Rossmeisl, J. Electrochemical CO Reduction: A Property of the Electrochemical Interface. *J. Am. Chem. Soc.* **141**, 1506-1514, doi:10.1021/jacs.8b08839 (2019).

23 Zou, X. *et al.* How Nitrogen-Doped Graphene Quantum Dots Catalyze Electroreduction of CO_2 to Hydrocarbons and Oxygenates. *ACS Catal.* **7**, 6245-6250, doi:10.1021/acscatal.7b01839 (2017).

24 Back, S., Lim, J., Kim, N.-Y., Kim, Y.-H. & Jung, Y. Single-atom catalysts for CO_2 electroreduction with significant activity and selectivity improvements. *Chem. Sci.* **8**, 1090-1096, doi:10.1039/c6sc03911a (2017).

25 Liu, H., Liu, J. & Yang, B. Modeling the effect of surface CO coverage on the electrocatalytic reduction of CO2 to CO on Pd surfaces. *Phys. Chem. Chem. Phys.* **21**, 9876-9882, doi:10.1039/C8CP07427E (2019).

26 Hirunsit, P. Electroreduction of Carbon Dioxide to Methane on Copper, Copper–Silver, and Copper–Gold Catalysts: A DFT Study. *J. Phys. Chem. C* **117**, 8262-8268, doi:10.1021/jp400937e (2013).

27 Peng, H.-J., Tang, M. T., Halldin Stenlid, J., Liu, X. & Abild-Pedersen, F. Trends in oxygenate/hydrocarbon selectivity for electrochemical $CO_{(2)}$ reduction to C_2 products. *Nat. Commun.* **13**, 1399, doi:10.1038/s41467-022-29140-8 (2022).

28 Mathew, K., Kolluru, V. S. C., Mula, S., Steinmann, S. N. & Hennig, R. G. Implicit self-consistent electrolyte model in plane-wave density-functional theory. *J. Chem. Phys.* **151**, 234101, doi:10.1063/1.5132354 (2019).

29 Mathew, K., Sundararaman, R., Letchworth-Weaver, K., Arias, T. A. & Hennig, R. G. Implicit solvation model for density-functional study of nanocrystal surfaces and reaction pathways. *J. Chem. Phys.* **140**, 084106, doi:10.1063/1.4865107 (2014).

30 Cheng, T., Xiao, H. & Goddard, W. A. Free-Energy Barriers and Reaction Mechanisms for the Electrochemical Reduction of CO on the Cu(100) Surface, Including Multiple Layers of Explicit Solvent at pH 0. *J. Phys. Chem. Lett.* **6**, 4767-4773, doi:10.1021/acs.jpclett.5b02247 (2015).

31 Montoya, J. H., Shi, C., Chan, K. & Nørskov, J. K. Theoretical Insights into a CO Dimerization Mechanism in CO_2 Electroreduction. *J. Phys. Chem. Lett.* **6**, 2032-2037, doi:10.1021/acs.jpclett.5b00722 (2015).

32 Calle-Vallejo, F. & Koper, M. T. M. Theoretical Considerations on the Electroreduction of CO to C_2 Species on Cu(100) Electrodes. *Angew. Chem. Int. Ed.* **52**, 7282-7285, doi:10.1002/anie.201301470 (2013).

33 Goodpaster, J. D., Bell, A. T. & Head-Gordon, M. Identification of Possible Pathways for C–C Bond Formation during Electrochemical Reduction of CO_2: New Theoretical Insights from an Improved Electrochemical Model. *J. Phys. Chem. Lett.* **7**, 1471-1477, doi:10.1021/acs.jpclett.6b00358 (2016).

34 Dupont, M. F. & Donne, S. W. Charge storage mechanisms in electrochemical capacitors: Effects of electrode properties on performance. *J. Power Sources* **326**, 613-623, doi:10.1016/j.jpowsour.2016.03.073 (2016).

35 Wang, S. *et al.* Electrochemical impedance spectroscopy. *Nat. Rev. Methods Primers* **1**, 41, doi:10.1038/s43586-021-00039-w (2021).

36 Xie, M. S. *et al.* Amino acid modified copper electrodes for the enhanced selective electroreduction of carbon dioxide towards hydrocarbons. *Energy Environ. Sci.* **9**, 1687-1695, doi:10.1039/C5EE03694A (2016).

37 Wakerley, D. *et al.* Bio-inspired hydrophobicity promotes CO_2 reduction on a Cu surface. *Nat. Mater.* **18**, 1222-1227, doi:10.1038/s41563-019-0445-x (2019).

38 Ahn, S. *et al.* Poly-Amide Modified Copper Foam Electrodes for Enhanced Electrochemical Reduction of Carbon Dioxide. *ACS Catal.* **8**, 4132-4142, doi:10.1021/acscatal.7b04347 (2018).
39 Wei, X. *et al.* Highly Selective Reduction of CO_2 to C_{2+} Hydrocarbons at Copper/Polyaniline Interfaces. *ACS Catal.* **10**, 4103-4111, doi:10.1021/acscatal.0c00049 (2020).
40 Pham, T. H. M. *et al.* Enhanced Electrocatalytic CO_2 Reduction to C_{2+} Products by Adjusting the Local Reaction Environment with Polymer Binders. *Adv. Energy Mater.* **12**, 2103663, doi:10.1002/aenm.202103663 (2022).
41 Dutta, A., Rahaman, M., Luedi, N. C., Mohos, M. & Broekmann, P. Morphology Matters: Tuning the Product Distribution of CO_2 Electroreduction on Oxide-Derived Cu Foam Catalysts. *ACS Catal.*, 3804-3814, doi:10.1021/acscatal.6b00770 (2016).
42 da Silva, A. H. M. *et al.* Electrocatalytic CO_2 reduction to C_{2+} products on Cu and Cu_xZn_y electrodes: Effects of chemical composition and surface morphology. *J. Electroanal. Chem.* **880**, 114750, doi:10.1016/j.jelechem.2020.114750 (2021).
43 Lee, S. Y. *et al.* Mixed Copper States in Anodized Cu Electrocatalyst for Stable and Selective Ethylene Production from CO_2 Reduction. *J. Am. Chem. Soc.* **140**, 8681-8689, doi:10.1021/jacs.8b02173 (2018).
44 Gao, D. *et al.* Selective CO_2 Electroreduction to Ethylene and Multicarbon Alcohols via Electrolyte-Driven Nanostructuring. *Angew. Chem. Int. Ed.* **58**, 17047-17053, doi:10.1002/anie.201910155 (2019).
45 De Luna, P. *et al.* Catalyst electro-redeposition controls morphology and oxidation state for selective carbon dioxide reduction. *Nat. Catal.* **1**, 103-110, doi:10.1038/s41929-017-0018-9 (2018).
46 Mistry, H. *et al.* Highly selective plasma-activated copper catalysts for carbon dioxide reduction to ethylene. *Nat. Commun.* **7**, 12123, doi:10.1038/ncomms12123 (2016).
